# Synthesis, Structure, Reactivity, and Intramolecular Donor–Acceptor Interactions in a Phosphinoferrocene Stibine and Its Corresponding Phosphine Chalcogenides and Stiboranes

**DOI:** 10.1021/acs.inorgchem.3c02075

**Published:** 2023-08-10

**Authors:** Jiří Schulz, Jakub Antala, David Rezazgui, Ivana Císařová, Petr Štěpnička

**Affiliations:** Department of Inorganic Chemistry, Faculty of Science, Charles University, Hlavova 2030, 128 40 Prague, Czech Republic

## Abstract

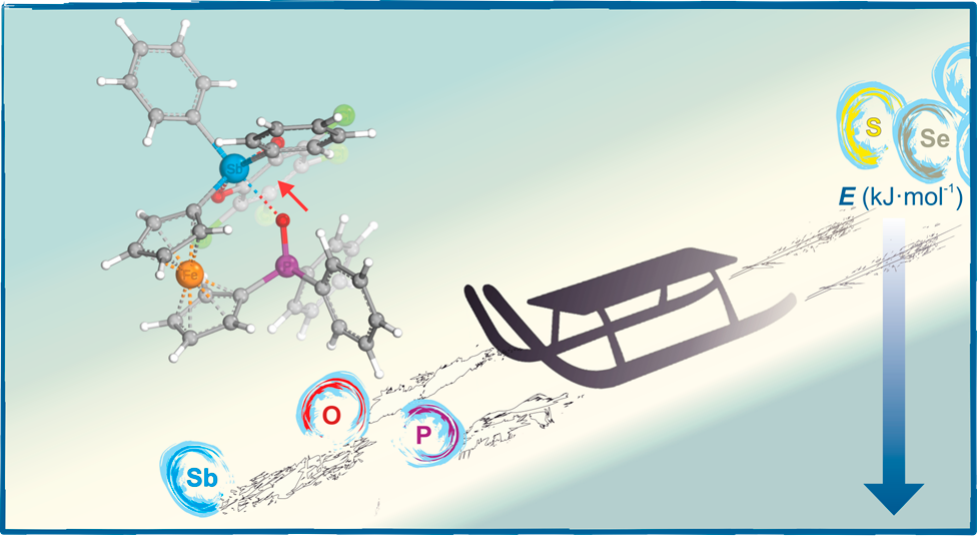

Ferrocene-based phosphines equipped with additional functional groups are versatile ligands for coordination chemistry and catalysis. This contribution describes a new compound of this type, combining phosphine and stibine groups at the ferrocene backbone, viz. 1-(diphenylphosphino)-1′-(diphenylstibino)ferrocene (**1**). Phosphinostibine **1** and the corresponding P-chalcogenide derivatives Ph_2_P(E)fcSbPh_2_ (**1E**, fc = ferrocene-1,1′-diyl, E = O, S, Se) were synthesized and further converted to the corresponding stiboranes Ph_2_P(E)fcSb(O_2_C_6_Cl_4_)Ph_2_ (**6** and **6E**) by oxidation with *o*-chloranil. All compounds were characterized by spectroscopic methods, X-ray diffraction analysis, cyclic voltammetry, and theoretical methods. Both NMR spectroscopy and DFT calculations confirmed the presence of P → Sb and P=O → Sb donor–acceptor interactions in **6** and **6O**, triggered by the oxidation of the stibine moiety into Lewis acidic stiborane. The corresponding interactions in **6S** and **6Se** were of the same type but significantly weaker. A coordination study with AuCl as the model metal fragment revealed that the phosphine group acts as the “primary” coordination site, in line with its higher basicity. The obtained Au(I) complexes were applied as catalysts in the Au-catalyzed cyclization of *N*-propargylbenzamide and in the oxidative [2 + 2 + 1] cyclization of ethynylbenzene with acetonitrile and pyridine *N*-oxides. The catalytic results showed that the stibine complexes had worse catalytic performance than their phosphine counterparts, most likely due to the formation of weaker coordination bonds and hence poorer stabilization of the active metal species. Nevertheless, the stibine moiety could be used to fine-tune the properties of the ligated metal center by changing the oxidation state or substituents at the “remote” Sb atom.

## Abstract 2

Manipulation of the functional substituents in 1-(diphenyphosphino)-1′-(diphenylstibino)ferrocene can be used to trigger intramolecular P···Sb and P=E···Sb (E = O, S, Sb) interactions, which have been described using a combination of experimental and theoretical approaches. Oxidation of the stibine moiety into a stiborane unit and the presence of the polarized P=O bond result in the strongest donor-acceptor interaction of this type. As a ligand, the title compound uses the phosphine moiety as a primary donor site.

## Introduction

**Scheme 1:**
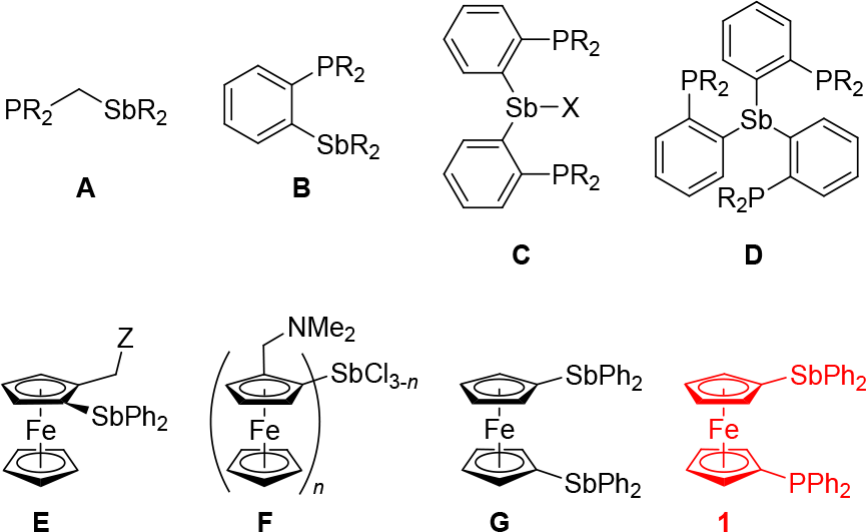
Examples of Phosphinostibine Donors (Top, for **A**–**D**: R = Various Alkyl and Aryl Groups; X = Cl or R) and Ferrocene Stibines (Bottom, for **E**: Z = NMe_2_, NHR, NMe_3_^+^I^–^, OH, OR, SR, etc.; for **F**: *n* = 2, 3) Studied to Date and the Structure of **1**

Hybrid ligands^1^ possessing distinct donor groups often exhibit coordination behavior and catalytic properties different from those of the corresponding monofunctional derivatives. In particular, phosphinoamine (P,N) ligands, combining the homologous donor moieties, stand out due to their structural versatility, the specific chemical properties and reactivity of the two donor groups, and the possibility of tuning their properties through substituents, resulting in wide catalytic applications.^2,3^ A generally similar situation is encountered in the case of phosphinostibine (P,Sb) ligands possessing heavier pnictogen atoms, which have been studied much less thus far. Even in this case, the donor groups significantly differ. Due to an inefficient mixing of the valence s and p orbitals and their more diffuse nature,^4^ stibines are worse σ-donors and π-acceptors than phosphines^5^ and can even behave as electron density acceptors.^6^ The Lewis acidity of stibines can be enhanced by introducing electron-withdrawing substituents and, alternatively, by their oxidation to Sb(V) compounds (stiboranes), which differentiates them from their phosphorus analogues. Compared to the corresponding phosphines, stibines are less sterically demanding due to longer C–Sb bonds and smaller angles between the substituents, which can result in different coordination preferences.^5,7^ When combined in one molecule, the phosphine moiety often behaves as the “primary” coordination site, while the stibine group remains uncoordinated or forms additional interactions with Lewis acids or bases.^8,9^ Prominent examples of phosphinostibine ligands (Scheme 1) include compounds whose functional groups are connected by methylene or phenylene spacers (**A**^10^ and **B**(11)) and the multidonor ligands **C** and **D**.^12,13^

In the chemistry of ferrocene ligands,^14^ stibine functional groups have only rarely been used. Until recently, ferrocene stibines were limited mainly to compounds **E** and **F** comprising a 1,2-disubstituted ferrocene backbone, which have been studied with a focus on the possible D → Sb interactions (D = adjacent donor moiety; for examples, see Scheme 1).^15^ Earlier this year, we reported the synthesis of ferrocene distibine **G** (Scheme 1),^16^ a congener of the iconic ferrocene ligand 1,1′-bis(diphenylphosphino)ferrocene (dppf).^17^ The facile conversion of this compound into isolable stiboranes and the differences in the reaction behavior of **G** and dppf led us to focus now on the mixed-donor analogue **1**, which represents the missing link between the two symmetrical ligands (Scheme 1). In this contribution, we describe the preparation of this compound and various oxidized derivatives, viz. phosphine-stiborane and phosphine chalcogenide-stiboranes. The resulting compounds are analyzed in view of the difference between the pnictogen donor groups and their possible interactions, which are studied through a combination of experimental and theoretical approaches. Also reported are the results of our preliminary coordination study employing Au(I) as a probe metal ion and the applications of the prepared complexes in gold-catalyzed reactions.

## Results and Discussion

### Synthesis of Phosphinostibine **1** and the Corresponding P-Chalcogenides and Stiboranes

**Scheme 2:**
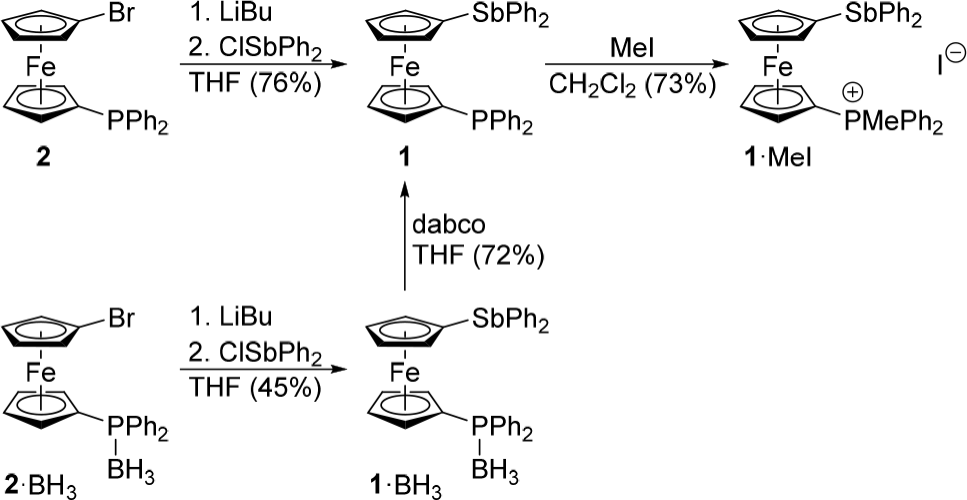
Synthesis of **1** and Its Reaction with MeI

Phosphinostibine **1** was prepared by lithiation of 1-bromo-1′-(diphenylphosphino)ferrocene (**2**) with *n-*butyllithium followed by the reaction of the *in situ* generated lithio intermediate with chlorodiphenylstibine (Scheme 2) and was isolated as an air-stable, orange crystalline solid in 76% yield after column chromatography and crystallization. A similar reaction employing 1-bromo-1′-(diphenylphosphino)ferrocene–borane (1:1) (**1**·BH_3_) produced the P-protected phosphinostibine **1**·BH_3_, which was smoothly deprotected^18^ with 1,4-diazabicyclo[2.2.2]octane (dabco)^19^ to give **1**.

**Figure 1:**
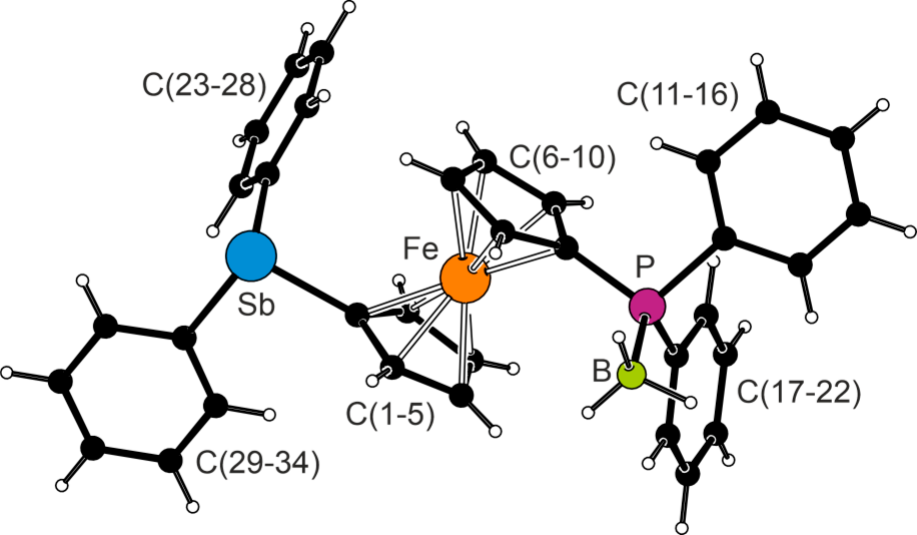
Molecular structure of **1**·BH_3_. Selected distances and angles (in Å and deg): Fe–C(1–10) (range) 2.031(3)–2.058(3), Sb–C1 2.131(3), Sb–C23 2.156(3), Sb–C29 2.165(3), C1–Sb–C23 95.6(1), C1–Sb–C29 94.2(1), C23–Sb–C29 96.4(1), P1–B 1.909(4), P–C6 1.789(2), P–C11 1.816(3), P–C17 1.807(3), C6–P–C11 104.5(1), C6–P–C17 107.4(1), C11–P–C17 104.8(1), B–P–C (range) 111.4(1)-114.6(1). A Displacement ellipsoid plot is available in the Supporting Information.

Compounds **1** and **1**·BH_3_ were characterized by NMR spectroscopy, ESI MS, and elemental analysis. The ^1^H and ^13^C{^1^H} NMR spectra displayed characteristic signals due to the asymmetrically 1,1′-disubstituted ferrocene units and the phenyl rings, whereas the ^31^P{^1^H} NMR spectra showed a sharp singlet for **1** (δ_P_ −16.4; cf. −16.2 for (diphenylphosphino)ferrocene^20^) and a broad doublet-like signal for **1**·BH_3_ (δ_P_ 16.4). Although compound **1** crystallized readily, its structure could not be determined with sufficient precision due to disorder. In the crystal, the P and Sb atoms alternated in their positions with only a minor effect on the overall arrangement, which controlled the crystal packing. Notably, this property was also observed for P-chalcogenides Ph_2_P(E)fcSbPh_2_ (fc = ferrocene-1,1′-diyl) with lighter chalcogen atoms (O and S), which formed shorter P=E bonds (*vide infra*). In contrast, the BH_3_ moiety in **1**·BH_3_ sufficiently “differentiated” the substituents and, thus, allowed the crystal structure to be determined (Figure 1). The structure of **1**·BH_3_ comprises a regular ferrocene unit with parallel cyclopentadienyl rings (dihedral angle 1.5(2)°) and substituents in approximately *anti* positions (the torsion angle τ = C1–Cg1–Cg2–C6, where Cg1 and Cg2 denote the centroids of the cyclopentadienyl rings C(1–5) and C(6–10), respectively, is 160.3(2)°; see Figure S17). The arrangement of the stibine substituent was similar to that in **G**(16) or 1-(diphenylstibino)-2-vinylferrocene,^15b^ i.e., with Sb–C(Ph) distances slightly longer than the Sb–C(ferrocenyl) bond and with C(Ph)–Sb–C(Ph) angles wider than the C(ferrocenyl)–Sb–C(Ph) angles. In turn, the geometry of the phosphine part compared well with that in dppf·2BH_3_^21^ or 1-(diphenylphosphino)-1-methylferrocene–borane (1:1).^22^

**Scheme 3:**
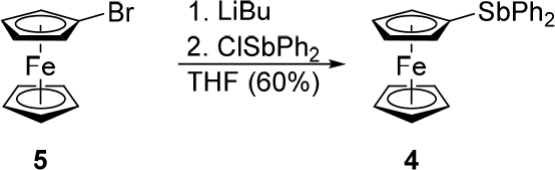
Synthesis of (Diphenylstibino)ferrocene (**4**)

To compare the donor groups present in **1** and to follow their possible interactions, we also prepared the corresponding monofunctional compounds, *viz*. (diphenylphosphino)ferrocene (**3**) and (diphenylstibino)ferrocene (**4**). The previously unreported stibine **4** was synthesized analogously to **1** (Scheme 3), i.e., by the lithiation of bromoferrocene (**5**) with *n*-butyllithium and subsequent reaction of chlorodiphenylstibine. The compound was isolated as an air-stable, orange crystalline solid in 60% yield by crystallization and was fully characterized, including structure determination (Supporting Information; Figure S13).

To quantify the differences between the phosphine and stibine donor groups, we calculated the methyl cation affinities (MCA)^23^ of **1**, **3**, and **4** (Table 1). Defined as the enthalpy of [LB–CH_3_]^+^ dissociation (LB = Lewis base), larger MCA values are obtained for stronger Lewis bases (LB). In the present case, the MCA values clearly differentiated the pnictogen donor groups in the model compounds, suggesting that the stibine derivative had lower basicity. The presence of the other substituent in the molecule of **1** had only a minor effect on the MCA values estimated for the individual pnictogen substituents (cf. the respective values for **1** and **3**/**4**). The inclusion of solvation phenomena significantly influenced the MCA values, but the general trend remained the same.

**Table 1:** Methyl Cation Affinities (MCAs in kJ mol^–1^ at 298.15 K) for **1**, **3**, and **4**^a^

compound	vacuum	chloroform
**1 (P)**	672	532
**1 (Sb)**	549	413
**3**	666	533
**4**	546	430

a Calculated at the PBE0(d3)/def2-TZVP:sdd(Fe,Sb) level of theory. Solvent effects have been approximated using the PCM model. For **1**, the site at which methylation occurred is specified.

These theoretical results corresponded with the outcome of a simple reaction test showing that methylation of **1** with methyl iodide proceeded selectively at the phosphorus atom to afford **1**·MeI in 73% isolated yield (Scheme 2), whereas the stibine group remained intact even when 2 equiv of MeI was used.^24^ Selective formation of **1**·MeI was manifested in the NMR spectra, which displayed only one signal attributable to the methyl group, split into a doublet because of interaction with ^31^P (δ_H_ 3.05, ^2^*J*_PH_ = 13.2 Hz; δ_C_ 11.64, ^1^*J*_PC_ = 60 Hz), while the ^31^P{^1^H} NMR signal was observed downfield relative to that of the parent compound (δ_P_ 24.2; cf. 22.6 for **3**·MeI in CD_3_CN^25^). An ultimate structure confirmation was provided by X-ray diffraction analysis (see the Supporting Information, Figures S9 and S10). Correspondingly, no borane scrambling between the phosphine and stibine moieties was observed for borane adduct **1**·BH_3_ in solution, consistent with the higher basicity of the phosphine group that renders the P–B adduct more stable.^26^

**Scheme 4:**
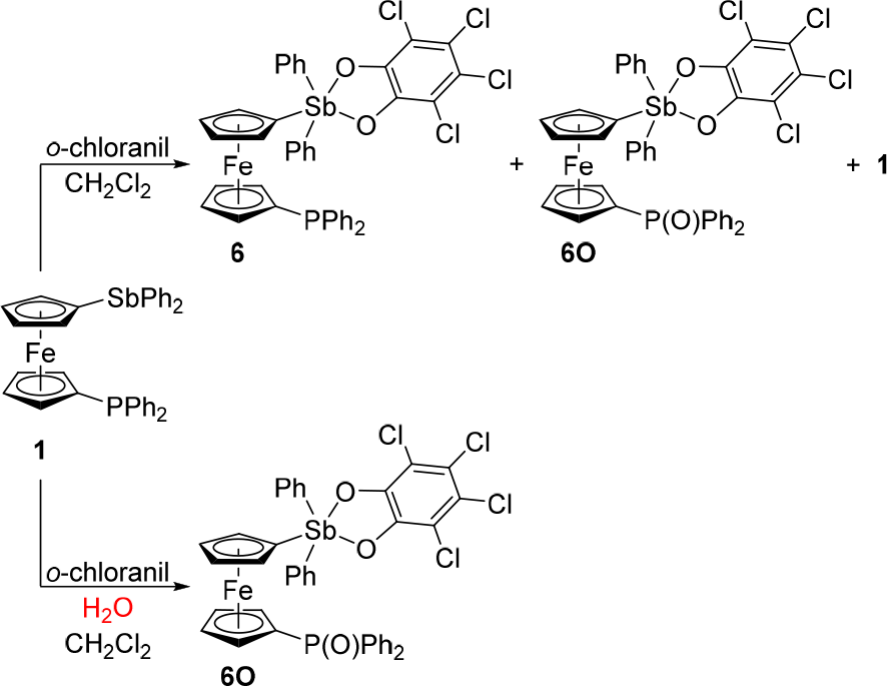
Oxidation of **1** with *o*-Chloranil

No evidence of a P → Sb donor interaction in **1** was observed. To increase the Lewis acidity of the Sb atom and thus make it amenable for the formation of P → Sb dative interactions, we converted phosphinostibine into phosphinostiborane **6** by oxidation with 3,4,5,6-tetrachloro-1,2-benzoquinone (*o*-chloranil)^27^ (Scheme 4). According to the results of the NMR analysis, the reaction of *o*-chloranil (1 equiv) with **1** in dichloromethane (1 h/room temperature) produced a mixture of the expected compound **6**, the corresponding phosphine oxide **6O**, and unreacted **1** (ratio of **6**:**6O**:**1** = 28%:35%:37%). The oxidation reaction lacked the selectivity observed in the similar oxidation of 1,2-Ph_2_PC_6_H_4_SbPh_2_, during which only the stibine moiety was oxidized.^28^ Nevertheless, our experiments suggested that the stibine moiety in **1** was oxidized preferentially because the compound Ph_2_P(O_2_C_6_Cl_4_)fcSbPh_2_ with an intact stibine moiety was not detected in the crude reaction mixture. The formation of **6O** can be explained by the 2-fold oxidation of **1** producing Ph_2_P(O_2_C_6_Cl_4_)fcSbPh_2_(O_2_C_6_Cl_4_) and subsequent (partial) hydrolysis by traces of water.^29^ Indeed, performing the reaction under dry conditions but with commercial *o*-chloranil improved the yield of **6** and decreased the amount of hydrolysis product **6O** (**6**:**6O**:**1** = 37%:29%:34%). The components of the reaction mixture were separated by chromatography. Despite the changes in the crude product composition, the isolated yield of **6** remained at approximately 20% due to reactions of this compound with the stationary phase used (silica gel) that resulted in irreversible binding, presumably after hydrolysis of the stiborane moiety. The facile hydrolysis of the presumed doubly oxidized intermediate Ph_2_P(O_2_C_6_Cl_4_)fcSbPh_2_(O_2_C_6_Cl_4_) was advantageously used to prepare **6O**, which was obtained as the main product upon adding 2 equiv of *o*-chloranil to a dichloromethane solution of **1** containing a few drops of water. Subsequent workup and crystallization afforded **6O** in 65% isolated yield (Scheme 4).

**Scheme 5:**
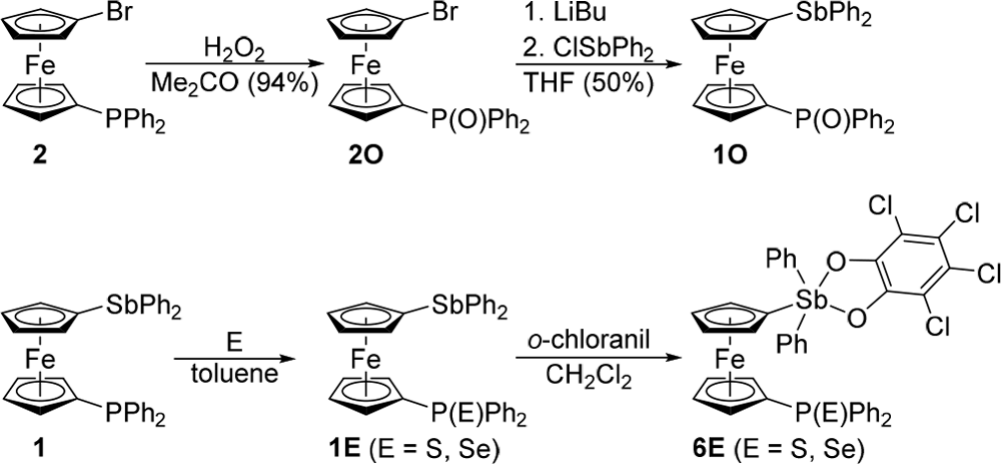
Synthesis of Phosphine Chalcogenides **1E** and the Corresponding Stiboranes

Next, the family of stibine and stiborane derivatives was expanded by compounds featuring heavier P-chalcogenides^30^ to establish the possible influence of the chalcogenide donor atom on the E → Sb interaction (Scheme 5). The oxidation changes not only the possible donor atom and the donor···Sb distance but also the electron density distribution in the system. Thus, phosphine oxide **1O** was obtained in two steps from phosphine-bromide **2**, which was oxidized by hydrogen peroxide to **2O** and subsequently lithiated and reacted with ClSbPh_2_ to produce **1O** (47% yield over the two steps after crystallization; N.B. the direct oxidation of **1** with hydrogen peroxide was not used due to side reactions at the stibine moiety). Phosphine sulfide **1S** and selenide **1Se** were obtained directly by reacting **1** with the corresponding chalcogens in refluxing toluene. The yields were 92% and 83% after crystallization, respectively. Compounds **1S** and **1Se** underwent clean oxidations with *o*-chloranil (1 equiv) to produce **6S** and **6Se**, respectively (∼95%; ∼5% of **1E** remained unreacted). Despite practically complete conversion, the sulfide was purified by column chromatography and isolated in only 67% yield because it remained partly adsorbed on the silica gel column (most likely after hydrolysis, *vide supra*). Selenide **1Se** could not be purified similarly due to decomposition and adsorption on the column. Alternatively, it was crystallized from hot heptane (66% yield). Increasing the amount of oxidant to 1.1 equiv resulted in complete conversion but also led to decomposition during isolation.

The oxidized phosphine moieties in stibines **1E** (E = O, S, and Se) showed characteristic, downfield-shifted ^31^P NMR signals (Table 2) and increased *J*_PC_ coupling constants^31^ compared to **1**. Notably, the chemical shifts were similar to the values reported for chalcogenides derived from **3** (FcP(E)Ph_2_; E = O, δ_P_ 30.3,^32^ E = S, 41.2 in C_6_D_6_,^33^ and E = Se, 32.7;^20b^ Fc = ferrocenyl), which indicated the absence of significant P=E···Sb interactions. The^1^*J*_SeP_ coupling constant determined for **1Se** (735 Hz) was higher than that for FcP(Se)Ph_2_ (731 Hz),^20b^ suggesting a lower basicity of the phosphine group^34^ in **1**, which can be ascribed to the presence of an electron-withdrawing stibine substituent that decreased electron density at the ferrocene unit and thus rendered the phosphine less basic.

**Table 2:** ^31^P NMR Shifts (δ_P_ in ppm) of Compounds **1** and **6**^a^

compound	δ_P_	compound	δ_P_	Δδ_P_^c^
**1**	–16.4	**6**	–9.7	+6.7
**1O**	29.3	**6O**	39.1	+9.8
**1S**	41.9	**6S**	41.4	–0.5
**1Se**	32.1 [735]^b^	**6Se**	31.8 [726]^b^	–0.3

a The spectra were recorded in CDCl_3_ at 25 °C.

b ^1^*J*_SeP_ coupling constant in Hz.

c Chemical shift difference between **6** and **1**.

**Figure 2:**
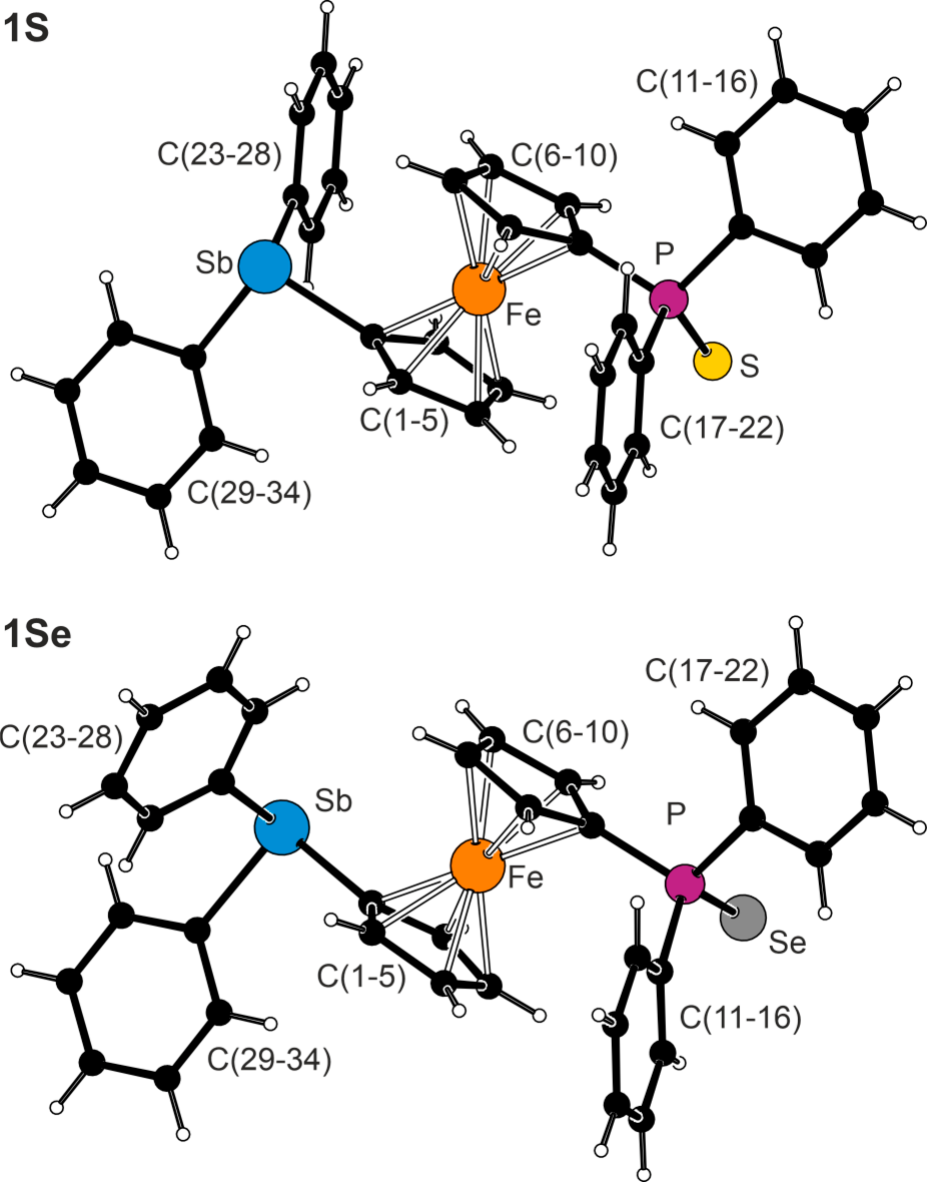
Molecular structures of **1S** and **1Se** (for the sake of clarity, only one position of the disordered substituents in **1S** is shown).

The structure determination of **1S** and **1Se** ruled out the presence of Sb···E interactions even in the solid state (the structure of **1O** was severely disordered and could not be satisfactorily refined). The sulfide **1S** crystallized as a racemic twin (monoclinic space group *Cc*) with positional disorder of the SbPh_2_ and P(S)Ph_2_ moieties, similar to **1** (*vide supra*). No such problems were encountered in the structure of **1Se**. The structures of **1S** and **1Se** were generally similar (Figure 2 and Table 3) with parallel cyclopentadienyl rings and substituents in approximately *anti* positions (see the τ angles in Table 3). A difference was observed in the mutual positioning of the substituents as the Ph_2_P(S) group was directed with its S atom away from the lone pair at the Sb atom, while the Se atom in **1Se** pointed in the same direction (Figure S17). The parameters of the stibine group were similar to those in **G** and **1**·BH_3_, while the geometry of the phosphorus substituents compared well with those observed in FcP(S)Ph_2_,^33^ dppfE_2_,^35^ and related compounds.^36^

**Table 3:** Selected Distances and Angles for **1S** and **1Se** (in Å and deg)

parameter^a^	**1S** (E = S)^c^	**1Se** (E = Se)
Sb–C1	2.125(3)	2.130(2)
Sb–C23/C29	2.160(4)/2.146(4)	2.156(2)/2.165(2)
C–Sb–C^b^	94.9(2)–97.2(2)	94.06(7)–96.74(7)
P=E	1.954(2)	2.1034(7)
P–C6	1.796(4)	1.784(2)
P–C11/C17	1.816(4)/1.806(4)	1.810(2)/1.811(2)
C–P–C^b^	104.9(2)–106.8(2)	104.72(8)–107.25(8)
Fe–C	2.036(4)–2.060(4)	2.034(2)–2.059(2)
tilt	2.0(2)	1.2(1)
τ	169.0(3)	–161.4(1)

a Fe–C is the range of the Fe–C(1–10) bond lengths, tilt stands for the dihedral angle of the least-squares cyclopentadienyl planes C(1–5) and C(6–10), and τ denotes the torsion angle C1–Cg1–Cg2–C6, where Cg1 and Cg2 are the centroids of the respective cyclopentadienyl rings.

b The range of the C1–Sb–C23/29 and C23–Sb–C29 angles.

c Data for the major orientation.

In contrast, the NMR spectra of stiboranes **6** and **6O** suggested possible P → Sb and P=O → Sb interactions, as the ^31^P NMR signals shifted downfield relative to those of respective stibines **1** and **1O** (Table 2). For phosphinostiborane **6**, the interaction was further indicated by the splitting of the ^13^C{^1^H} NMR signals due to CH and C^ipso^ carbons in the Sb-bound C_5_H_4_ ring and C^ipso^ of SbPh_2_ with ^31^P, while no such coupling was observed for **1**. Conversely, the ^1^H NMR spectra remained virtually unaffected, displaying only signals attributable to a conformationally unconstrained, P^III^-substituted ferrocene-1,1′-diyl unit, namely, three apparent triplets and one apparent quartet due to the C_5_H_4_ rings, albeit at a lower field compared to **1** because of the increased electron-withdrawing character of the stiborane substituent (this is consistent with the trend in the redox potentials of ferrocene oxidation, *vide infra*).

**Figure 3:**
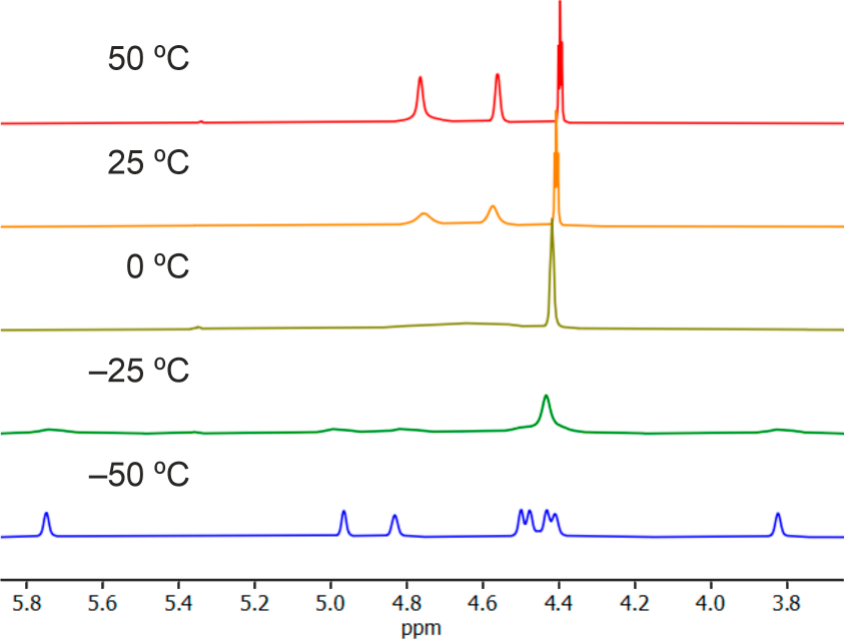
VT ^1^H NMR spectra (400 MHz, CDCl_3_) of **6O** showing the region of ferrocene protons (complete spectra are available in the Supporting Information).

In addition, the ^1^H and ^13^C{^1^H} NMR spectra of **6O** were broadened, suggesting a dynamic structure on the NMR time scale. This was confirmed by a VT ^1^H NMR study (Figure 3 and Figure S1): the spectrum recorded at −50 °C displayed eight separate signals for the ferrocene CH groups, which became diastereotopic due to a fixed conformation at a low temperature (the ferrocene moiety became axially chiral). Upon increasing the temperature, the signals broadened, and at 25 °C, only three signals were observed for the C_5_H_4_ protons due to time averaging.

**Figure 4:**
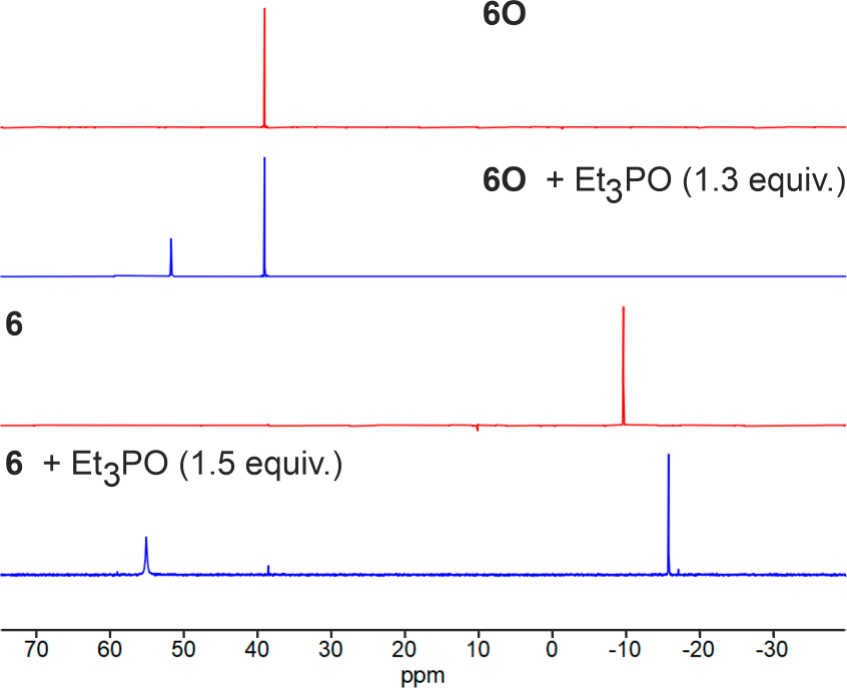
^31^P{^1^H} NMR spectra (162 MHz, CDCl_3_, 25 °C) of **6**, **6O**, and their mixtures with Et_3_PO.

Attempts to disrupt the intramolecular P=O → Sb interaction through the addition of competing Lewis bases failed. The NMR spectrum of **6O** remained unchanged upon addition of 4-(dimethylamino)pyridine (1.0 equiv), triethylphosphine oxide (1.3 or 10 equiv), or triphenylphosphine chalcogenides (1.5 equiv; Figure 4 and Figures S2–S4) in CDCl_3_, and even the spectrum recorded in DMSO-*d*_6_ as a strongly donating solvent suggested that an intramolecular interaction was present. Conversely, the addition of BF_3_·OEt_2_ (1 or 5 equiv) as a competing Lewis acid to **6O** cleaved the P=O → Sb dative bond, presumably with concomitant formation of the phosphine oxide–borane adduct Ph_2_P(O)fcSbPh_2_(O_2_C_6_Cl_4_)·BF_3_ (Figure S5). Analogous reaction with B(C_6_F_5_)_3_ resulted in decomposition. Similar competing experiments with **6** showed that the intramolecular P → Sb interaction was efficiently canceled by adding 1.5 equiv of Et_3_PO, very likely with concomitant formation of **6**·Et_3_PO (Figure 4).

**Figure 5:**
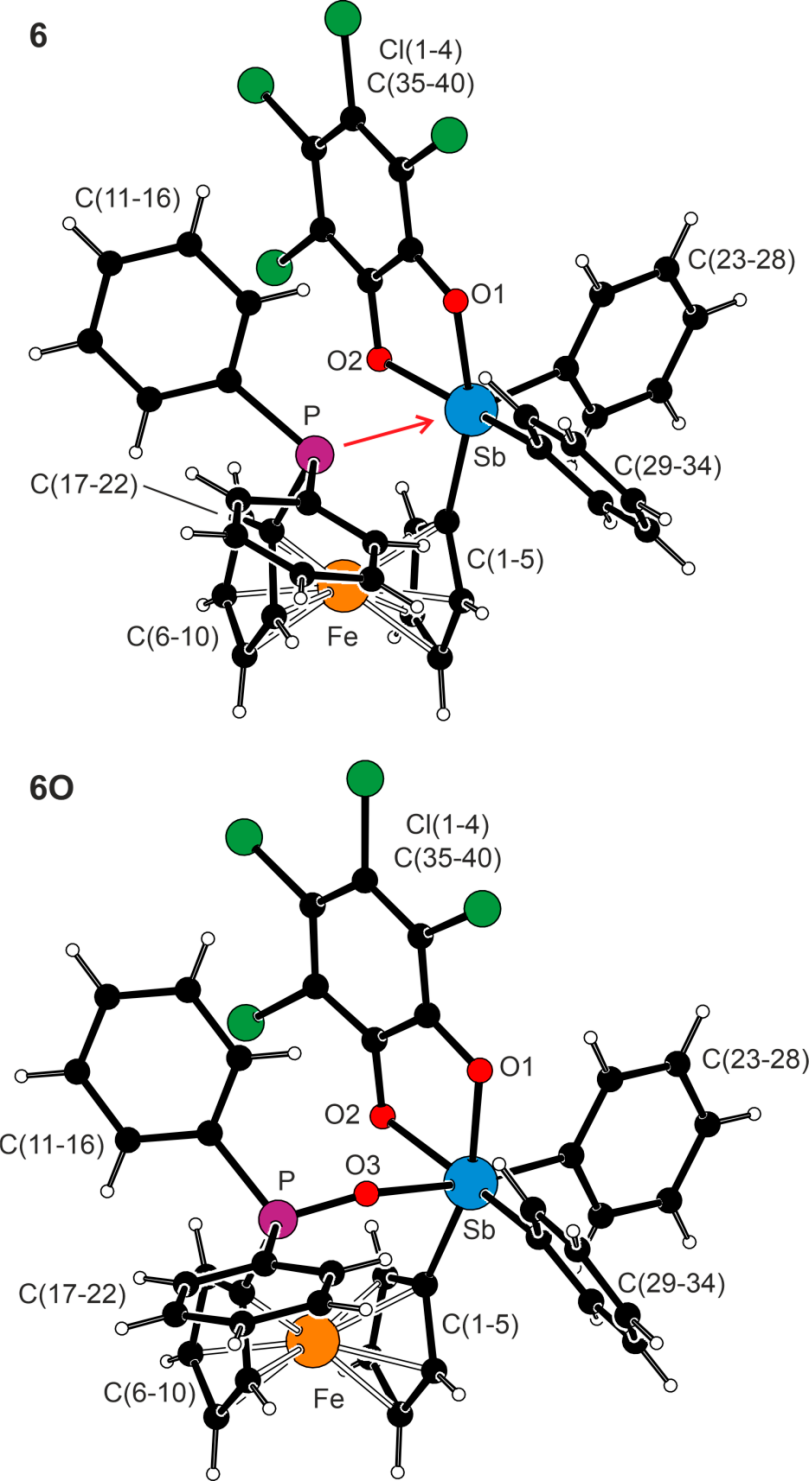
Molecular structures of **6**·C_6_H_14_ and **6O**·CHCl_3_ (the solvent molecules and the less populated orientation of phenyl ring C(17–22) in the molecule of **6** have been omitted for clarity). The P → Sb interaction in molecule **6** is indicated by a red arrow.

The intramolecular interactions were clearly detected in the crystal structures of **6**·C_6_H_14_ and **6O**·CHCl_3_ (Figure 5 and Table 4); compounds **6S** and **6Se** did not provide suitable crystals despite numerous attempts. The P → Sb interaction in the molecule of **6** was suggested by the short P···Sb distance (3.0987(6) Å), which is approximately halfway between the sum of the van der Waals radii (3.86 Å)^37^ and the sum of the covalent radii (2.46 Å) of these atoms.^38^ Compared to **1**·BH_3_ containing an intact SbPh_2_ group, the substituents at the Sb atom were moved apart to provide space for the phosphorus lone pair. For **6**·C_6_H_14_, this can be illustrated by wider C–Sb–C angles (∼99–102°) and, mainly, by the τ_5_ index of 0.03, which was close to the value expected for an ideal square pyramid (τ_5_ = 0; an ideal trigonal bipyramid yields τ_5_ = 1).^39^ The Sb atom was located 0.28 Å above the {O1, O2, C1, and C29} basal plane, whose minor distortion resulted from the narrower O1–Sb–O2 angle (78.93(5)°) associated with the chelating catecholate ligand (the remaining angles between the basal donor atoms were 86.84(6)–101.95(6)°).

**Table 4:** Selected Distances and Angles for **6** and **6O**·CHCl_3_ (in Å and deg)

parameter^a^	**6**·C_6_H_14_ (X = P)	**6O**·CHCl_3_ (X = O3)^d^
Sb···X	3.0987(6)	2.256(1)
Sb–O1/2	2.068(1)/2.082(1)	2.064(1)/2.076(1)
Sb–C1	2.127(2)	2.116(2)
Sb–C23/C29	2.124(2)/2.138(2)	2.147(2)/2.126(2)
C–Sb–O^b^	161.65(5)/159.94(5)	160.93(6)/162.59(6)
P–C6	1.811(2)	1.779(2)
P–C11/C17	1.839(2)	1.803(2)/1.796(2)
C6–P–C11/C17	101.90(8)/–c	107.07(7)/107.07(8)
C11–P–C17	101.3(1)	106.04(8)
Fe–C	2.022(2)–2.057(2)	2.034(2)–2.049(2)
tilt	4.0(1)	2.9(1)
τ	–14.1(1)	5.1(1)

a The parameters are defined as for compounds **1S** and **1Se**. See footnote to Table 3.

b The C1–Sb–O1/C29–Sb–O2 angles for **6**·C_6_H_14_, and C29–Sb–O1/C1–Sb–O2 angles for **6O**·CHCl_3_.

c Value uncertain due to disorder of phenyl ring C(17–22).

d Further data: P–O3 1.506(1) Å, P–O3–Sb 142.69(7)°.

Despite the vicinity of the phosphoryl oxygen atom O3, the geometry around the Sb atom in **6O**·CHCl_3_ remained square pyramidal (τ_5_ = 0.03) and was similarly distorted. The Sb atom was displaced by 0.23 Å from the basal plane, and the O3–Sb–C23 angle was nearly linear (172.05(5)°). Even in this case, the Sb···O3 separation (2.256(1) Å) was well below the sum of the van der Waals radii (3.58 Å) but longer than the sum of the covalent radii (2.05 Å). Notably, the O···Sb distance in **6O**·CHCl_3_ was significantly shorter than that in (2-Ph_2_P(O)C_6_H_4_SbPh_3_)[BF_4_] (2.432(2) Å), where the P–O···Sb fragment is bent (∼116°) due to the geometric constraints imposed by the *o*-phenylene backbone.^40^ Compared to **6**, compound **6O** showed shorter P–C bonds and wider C–P–C angles, which is a trend detectable also in the FcPPh_2_ (**3**)/FcP(O)Ph_2_ pair.^41^

### Analysis of the Bonding Situation

The nature of the P/P=E → Sb interactions was studied by DFT calculations. Initially, we analyzed the calculated electron densities using the quantum theory of atoms in molecules (QTAIM) approach.^42^ The key parameters are summarized in Table 5 and in the Supporting Information (Figure S24).

**Table 5:** Electron Densities (ρ_bcp_), Laplacians of the Electron Density (∇^2^ρ_bcp_), Total Electron Densities (*H*), Ratios of Potential and Kinetic Energy Density (|*V*|/*G*), and Ratios of Kinetic (*G*/ρ_bcp_) and Total Energy Density (*H*/ρ_bcp_) to Electron Density at the Bond Critical Point (bcp) Located between the Antimony Atom and a Donor Atom and the Experimental and Calculated Bond Distances

		bond length (Å)							
compd	bond	exp	calc^a^	ρ_bcp_ (e Å^–3^)	∇^2^ρ(r) (e Å^–5^)	*H* (au)	|*V*|/*G* (au)	*G*/ρ_bcp_ (au)	*H*/ρ_bcp_ (au)
**6**	P···Sb	3.0987(6)	2.975	0.037	0.028	–0.75 × 10^–2^	1.77	0.26	–0.20
**6O**	O···Sb	2.256(1)	2.293	0.055	0.173	–1.22 × 10^–2^	1.44	0.51	–0.22
**6S**	S···Sb	na^b^	2.915	0.034	0.044	–0.56 × 10^–2^	1.48	0.35	–0.17
**6Se**	Se···Sb	na^b^	2.996	0.034	0.034	–0.62 × 10^–2^	1.60	0.30	–0.18

a Calculated at the PBE0(d3)/def2-TZVP:sdd(Sb,Fe) level of theory.

b Not available.

The Laplacian of the electron density at the bond critical points (∇^2^ρ_bcp_) between antimony and the donor atom was positive for all stiboranes, indicating some type of closed-shell interaction (ionic, dative, or van der Waals). These weak noncovalent interactions can be distinguished from dative bonds by comparing the relative amounts of potential and kinetic energy at the bond critical point (bcp).^43,44^ Specifically, a covalent bonding interaction is indicated by a potential energy density (*V*_bcp_, always negative) greater than the kinetic energy density (*G*_bcp_, always positive), which yields a negative total energy density (*H* = *V* + *G* < 0) or, alternatively, |*V*|/*G* > 1. The pertinent values indicated that the bonding interactions in stiboranes **6** and **6E** were dative bonds (P → Sb or P=E → Sb). Further inspection of the ratios of the kinetic and total energy density to the electron density (ρ_bcp_), viz. *G*/ρ_bcp_ and *H*/ρ_bcp_, revealed a slightly higher electrostatic contribution for the P=E → Sb interactions (reflected by more positive *G*/ρ_bcp_ values) than for the P → Sb bond and a comparable degree of covalency (reflected by similarly negative *H*/ρ_bcp_ values). Both indices were the highest for stiborane **6O**, suggesting the strongest interaction in this compound, which corresponds with the experimental results.

This was consistent with the calculated energy difference between the “open” and “closed” forms of stiboranes **6** and **6O** (Table 6), which clearly favored the closed form, where the phosphorus groups and stiborane moieties are oriented toward each other and interact (for the structure diagrams, see the Supporting Information, Figure S25). The energy difference, which can be taken as a measure of the strength of the interaction (N.B. the closed form is expected to be destabilized sterically, which hampers the interaction), in absolute values, decreased from **6O** to **6** to **6S**/**6Se** for isolated species under a vacuum, and the same trend was noted even when considering the solvent effects. However, while the inclusion of solvation phenomena resulted in almost no change in the energy difference for **6**, the values for compounds featuring polar P=E bonds were more affected. In particular, the energy difference for the phosphine oxide **6O** decreased by 12 kJ mol^–1^ upon accounting for the solvation effects, which was attributed to the strong polarization of the P=O bond toward P^+^–O^–^.^45^ Notably, the energy differences determined for **6** and **6O** were significantly higher than the energy barrier for the rotation of the cyclopentadienyl rings in ferrocene itself, which was estimated to be approximately 4 kJ mol^–1^ in the gas phase;^46^ those in **6S** and **6Se** were of the same order. The P → Sb interaction in **6** was also manifested by a decreased fluoride ion affinity^47^ (305 kJ mol^–1^) compared to the model compound FcSbPh_2_(O_2_C_6_Cl_4_) (341 kJ mol^–1^), indicating a decreased Lewis acidity of the stiborane moiety in **6** as the result of P → Sb donation.^48^

**Table 6:** Free Energy Differences (Δ*G* in kJ mol^–1^ at 298.15 K) between the Open and Closed Isomers of Stiboranes **6**, **6O**, **6S**, and **6Se**^a^

compound	vacuum	chloroform
**6**	–26	–25
**6O**	–49	–37
**6S**	–10	–5
**6Se**	–10	–5

a Defined as Δ*G* = *G*(closed) – *G*(open) and calculated at the PBE0(d3)/def2-TZVP:sdd(Fe,Sb) level of theory. Solvent effects have been approximated using the PCM model.

**Figure 6:**
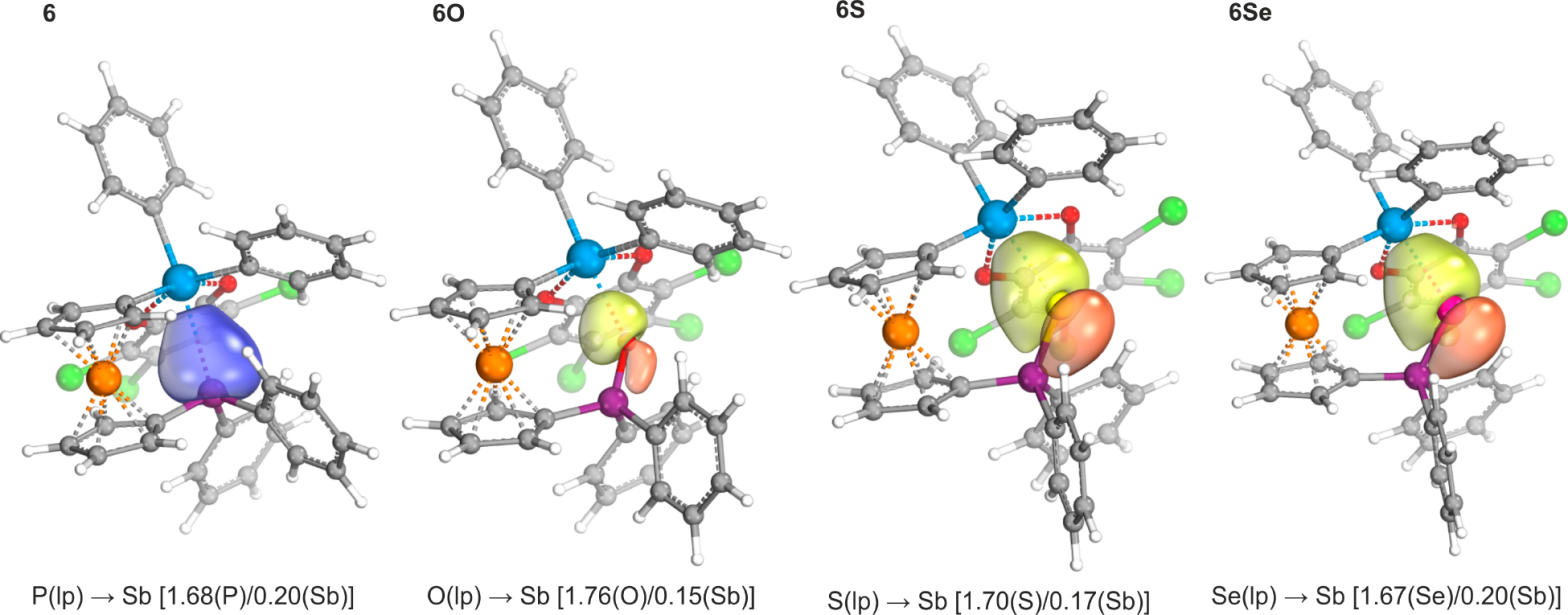
Selected intrinsic bond orbitals (IBOs) of stiboranes **6**, **6O**, **6S**, and **6Se**. Values in parentheses indicate the fraction of bonding electrons assigned to the individual atoms (lp = lone electron pair).

The presence of dative interactions was further indicated by the calculated Mayer bond orders (MBOs) and Wiberg bond indices (WBIs) (Table 7), which represent the number of electrons shared between two interacting atoms. The lowest values found for stiborane **6O** seemingly did not correspond to the experimental and theoretical results but indicated a more pronounced role for the electrostatic contribution to bonding. The dative interactions between the stiborane and phosphine/phosphine-chalcogenide moieties were visualized using intrinsic bond orbital (IBO) analysis.^49^ The identified IBOs corroborated the donation of electron density from lone electron pairs located on either the phosphorus or chalcogenide atom to antimony (Figure 6).

**Table 7:** Selected Mayer Bond Orders (MBOs) and Wiberg Bond Indices (WBIs) for **6** and **6E**^a^

	MBO		WBI	
compound	P/E···Sb	P=E	P/E···Sb	P=E
**6**	0.49	na	0.22	na
**6O**	0.13	1.44	0.19	1.92
**6S**	0.55	1.44	0.21	1.65
**6Se**	0.57	1.35	0.25	1.49

a Calculated at the PBE0(d3)/def2-TZVP:sdd(Fe,Sb) level of theory. na = not applicable.

The IBO analysis further revealed bonding differences between the phosphine chalcogenide moieties (see the Supporting Information, Figures S26–S28), although the overall description complied well with the generally accepted bonding scheme.^50^ The IBO corresponding to the P–O σ-bond in **6O** was largely located at the oxygen atom, confirming the highly polarized nature of this bond. In contrast, the corresponding IBOs in **6S** and **6Se** showed an equal distribution of the bonding electron pairs between the two atoms (phosphorus and chalcogen). Similarly, the π-component of the P–O bond in **6O** differed from those of its heavier congeners, as all three oxygen lone electron pairs were involved in π-interactions with the phosphorus atom, although two of them were involved only to a limited extent. For **6S** and **6Se**, only two electron pairs were involved in the π-bonding interaction, leaving one electron pair essentially nonbonding at the chalcogen atom.^51^

### Electrochemistry

**Figure 7:**
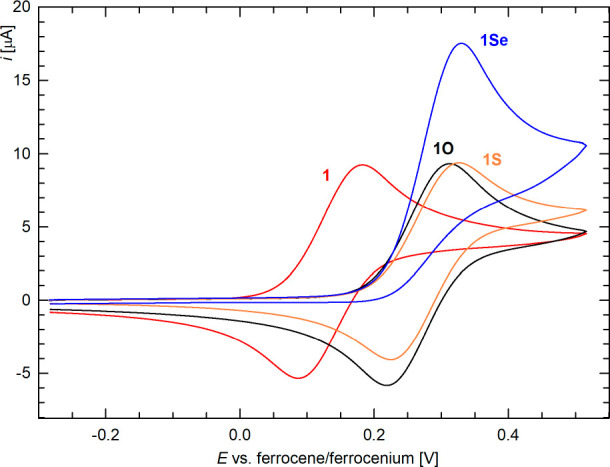
Cyclic voltammograms of **1** and **1E** (Pt-disk electrode, CH_2_Cl_2_, 0.1 M Bu_4_N[PF_6_], scan rate 0.1 V s^–1^).

The electrochemical behavior of compounds **1** and **6** was studied by cyclic voltammetry on a Pt-disk electrode in dichloromethane by using Bu_4_N[PF_6_] as the supporting electrolyte. In the accessible range (approximately −1.5 to 2 V vs the ferrocene/ferrocenium reference^52^), compound **1** showed a single redox transition (Figure 7), which was essentially reversible and diffusion-controlled, as indicated by *i*_pa_ ∝ ν^1/2^ (*i*_pa_ is the anodic peak current and ν the scan rate; Figures S18 and S19). Such behavior contrasted with the redox response of dppf^53^ and analogous compounds,^54^ whose electrochemical oxidation was typically associated with follow-up reactions that decreased the reversibility of the electrochemical oxidation. In line with the electron-withdrawing nature of the substituents in **1**, the oxidation occurred 0.14 V more positive than that of ferrocene itself (Table 8) but at a slightly lower potential than the oxidation of dppf under similar conditions (*E*°′ = 0.17 V vs ferrocene/ferrocenium),^53b^ reflecting the lower electronegativity of Sb. The redox responses of **1O** and **1S** were similar, except that the redox waves were shifted toward more positive potentials as a result of the increased electron-withdrawing character of the P(E)Ph_2_ substituents (Figure 7). The voltammogram of **1S** displayed an additional reductive wave when the scan range was extended toward more positive potentials (Figure S20). Conversely, the oxidation of **1Se** was irreversible and multielectron in nature.^55^ At more positive potentials, the selenide underwent another ill-defined oxidation (*E*_pa_ ≈ 0.89 V) and showed several weak reductive waves upon a reverse scan (Figure S20).

**Table 8:** Summary of the Electrochemical Data^a^

compound	*E*°′ (V)	compound	*E*°′ (V)
**1**	0.14	**6**	0.30
**1O**	0.27	**6O**	0.40
**1S**	0.29	**6S**	0.42
**1Se**	0.33^b^	**6Se**	0.48^b^

a The measurements were conducted at the Pt-disk electrode in dichloromethane containing 0.1 M Bu_4_N[PF_6_]. The potentials are given in volts relative to ferrocene/ferrocenium references (for details, see the Supporting Information). The potentials for reversible processes were determined as the average of anodic (*E*_pa_) and cathodic (*E*_pc_) peak potentials, *E°*′ = 1/2 (*E*_pa_ + *E*_pc_). The separation of the peaks in the cyclic voltammograms was approximately 90 mV due to a large resistance. The decamethylferrocene standard showed similar values.

b Irreversible wave. The anodic peak potential (*E*_pa_) at a scan rate of 100 mV s^–1^ is given.

**Figure 8:**
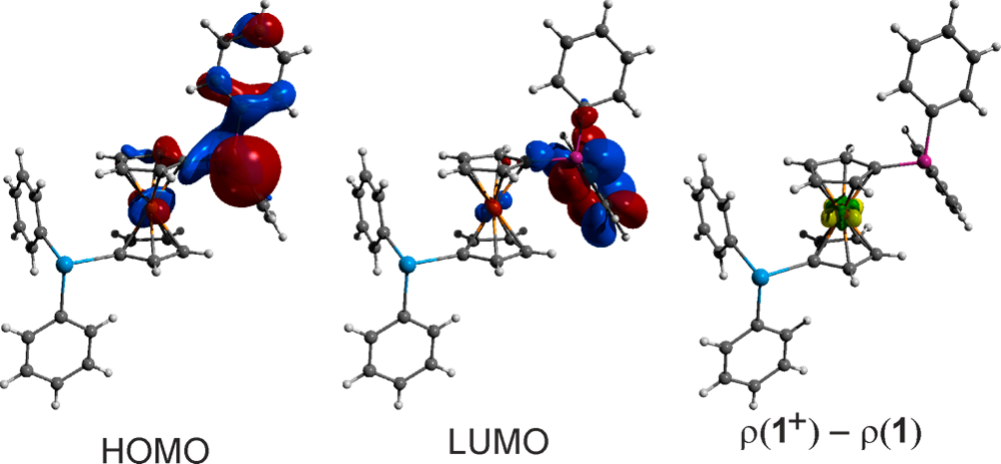
Frontier orbitals (isosurface at ±0.04 au) and the electron difference map ρ(**1**) – ρ(**1**^**+**^ mapped at the geometry of **1**) (isosurface at ±0.02 au) at the PBE0(d3)/def2TZVP level of theory.

Based on the results of DFT calculations, the primary oxidation of **1** was assigned to the ferrocene/ferrocenium transition. Although inspection of the frontier orbitals (Figure 8) using natural atomic orbitals (NAOs) showed that the HOMO of **1** corresponds mainly to the lone electron pair of the PPh_2_ group, being composed of the phosphorus 3s (∼11%) and 3p (∼47%) orbitals with a contribution from the 2p orbitals of the proximal carbon atoms (∼29%) and the iron 3d orbitals (∼5%), a change in the electron density from **1** to **1**^+^, ρ(**1**^+^) – ρ(**1**), mapped at the equilibrium geometry of **1**,^56^ occurred exclusively at the ferrocene iron, which supported the assignment of the electrochemical oxidation as ferrocene-based. Conversely, the LUMO of **1** was the antibonding combination of carbon 2p orbitals in the π-system of one phenyl ring at the PPh_2_ group.

**Figure 9:**
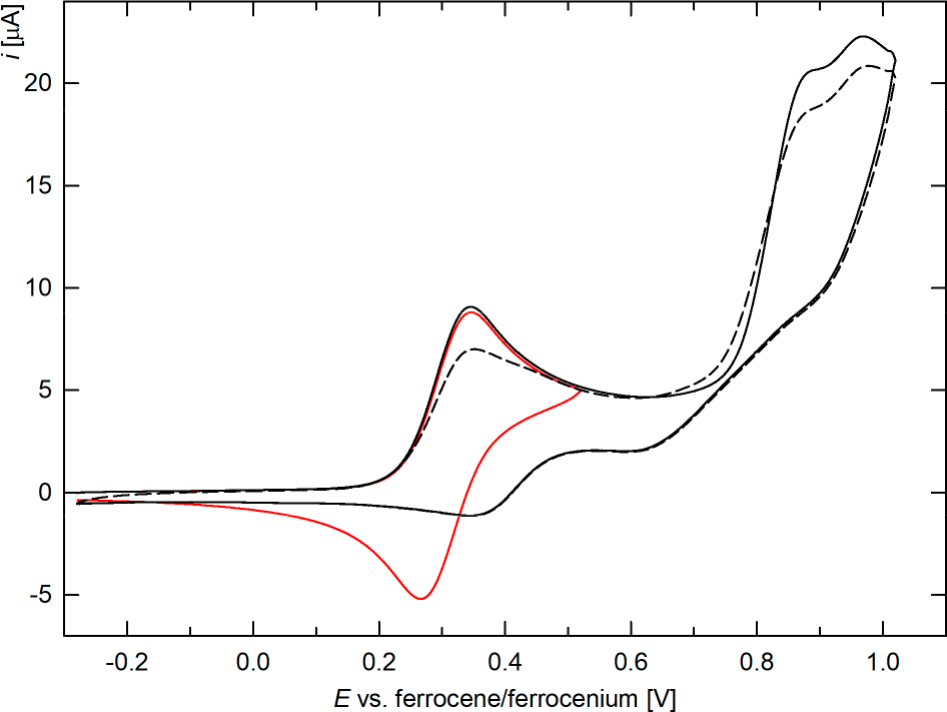
Cyclic voltammograms of **6** (Pt-disk electrode, CH_2_Cl_2_, 0.1 M Bu_4_N[PF_6_], scan rate of 0.1 V s^–1^). The second scan is shown by a dashed line. The peak potentials of the irreversible oxidations are approximately 0.87 and 0.97 V.

The first oxidation of catecholatostiboranes **6**, **6O**, and **6S** (Figure 9 and Figures S21–S23) was also reversible when they were scanned separately (i.e., when the switching potential was set just after the first redox wave). At higher potentials, however, the compounds displayed several irreversible oxidations, which affected the overall response (e.g., by decreasing the intensity of the first reductive wave and through additional broad reduction features) and resulted in weak reductive waves during the reverse scans. In the case of **6Se**, even the first oxidation was irreversible, and several weak reductive waves were observed, suggesting the instability of the electrochemically generated species. Compared to that of the corresponding stibines, the oxidation of **6** and **6E** was shifted by 0.13–0.16 V toward more positive potentials (Table 8), which indicated a decrease in the electron density at the ferrocene unit upon converting the stibine group into the strongly electron-withdrawing stiboranyl moiety.^57^

### Gold Complexes

**Scheme 6:**
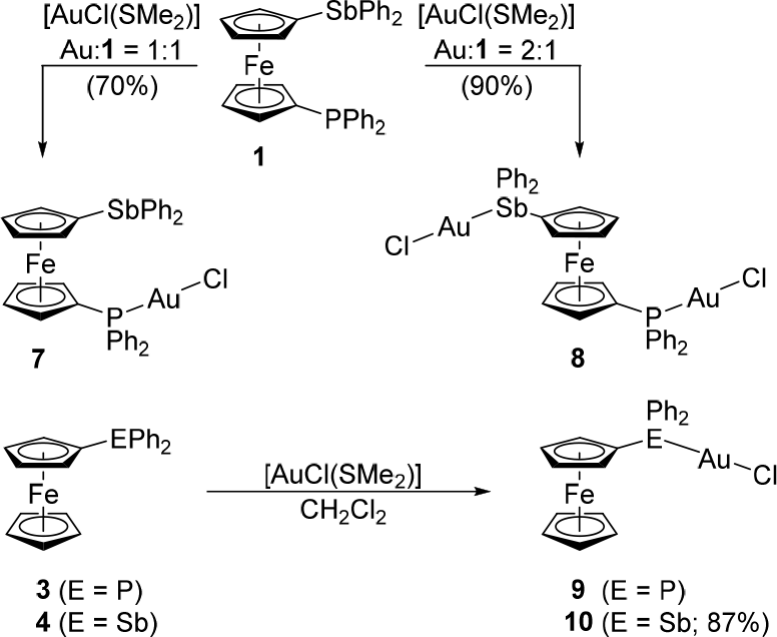
Synthesis of AuCl Complexes

In view of the intended catalytic testing, the coordination properties of **1** were investigated through reactions with Au(I) precursors. Thus, the reaction of **1** with [AuCl(SMe_2_)] at a 1:1 molar ratio in dichloromethane produced the phosphine complex [AuCl(**1**-κ*P*)] (**7**) as the sole product (Scheme 6). When the amount of the gold(I) precursors was increased to 2 equiv, a similar reaction afforded the digold(I) complex [(μ(P,Sb)-**1**)(AuCl)_2_] (**8**). Compared to **7**, however, digold complex **8** was *much* less stable, decomposing rapidly in solution and even when stored as a solid at low temperatures in the dark. Attempts to prepare P,Sb-bridged digold complexes via removal of chloride from **7** with Ag[SbF_6_] or by the reaction of **1** with [Au(tht)_2_][SbF_6_] (Au:**1** = 1:1) containing the easily dissociating tetrahydrothiophene ligands (tht) were unsuccessful. Reactions of model compounds **3** and **4** with [AuCl(SMe_2_)] (1 equiv) produced the respective chlorogold(I) complexes, [AuCl(FcPPh_2_-κ*P*)] (**9**)^58^ and [AuCl(FcSbPh_2_-κ*P*)] (**10**) (Scheme 6). Even in this pair, the stibine complex was considerably less stable than its phosphine analogue, decomposing in both solution and the solid state.

The coordination of the phosphine moiety was indicated by a downfield shift of the ^31^P{^1^H} NMR signal (δ_P_ 28.9 and 27.4 for **7** and **8**, respectively) and changes in the ^1^H and ^13^C{^1^H} NMR spectra (e.g., the ^13^C{^1^H} NMR signal due to C^ipso^-P in **7** was shifted to a higher field, and the ^1^*J*_PC_ coupling constant increased to 73 Hz from 7 Hz in the free ligand). The coordination of the stibine moiety had no distinct marker (such as the ^31^P chemical shift) but was indicated by changes in the ^1^H and ^13^C{^1^H} NMR spectra.^59^ For **9**, coordination increased the ^1^H NMR chemical shifts due to ferrocene protons, and the signal due to ferrocene C^ipso^ shifted upfield (signals due to CH groups experienced smaller changes). The ESI MS of **7** and **8** showed ions due to [M – Cl]^+^; the spectrum of **10** displayed a major peak due to [Au(**4**)_2_]^+^ resulting by ligand redistribution and a minor peak of [M – Cl + Me_2_CO]^+^.

**Scheme 7:**
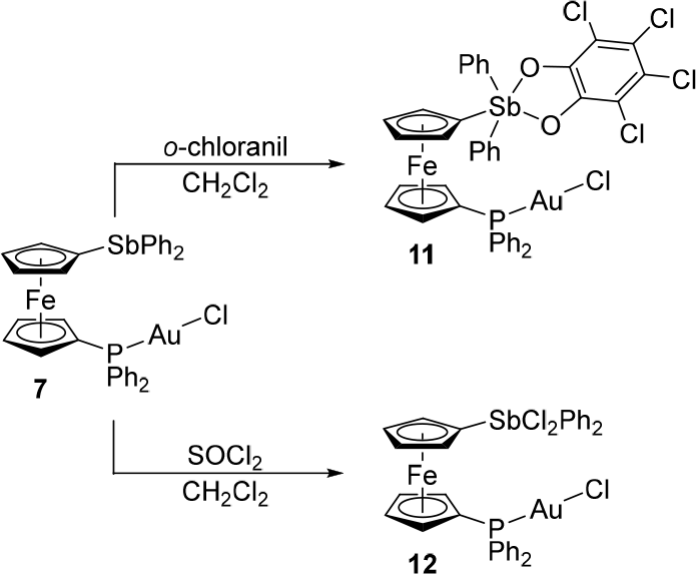
Oxidation of Complex **7**

Complex **7** reacted cleanly with *o*-chloranil and thionyl chloride to produce stable complexes **11** and **12**, respectively, which were isolated in 78% and 96% yields (Scheme 7). The NMR spectra of these compounds showed the expected signals, including those due to the tetrachlorocatecholate ligand for **11**. The ^31^P{^1^H} NMR resonances were only marginally affected (δ_P_ ≈ 28), and ESI MS revealed ions attributable to the sodiated species [M + Na]^+^.

**Figure 10:**
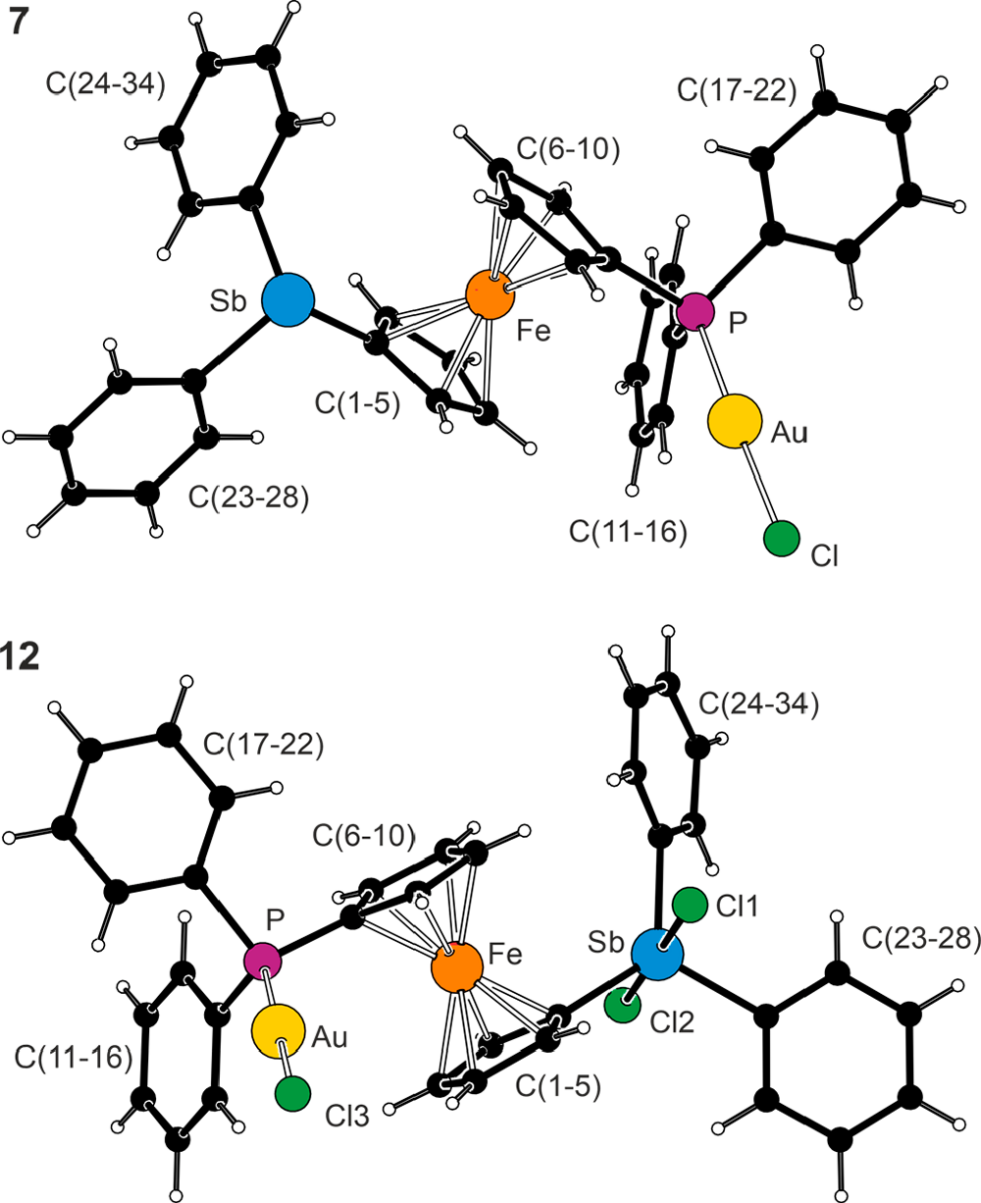
Views of the complex molecules in the structures of **7** and **12**·0.4CHCl_3_.

The structures of **7** and **12**·0.4CHCl_3_ (Figure 10 and Table 9) comprised linear P–Au–Cl moieties (∼175°) with Au–P and Au–Cl distances similar to those in [AuCl(PPh_3_)]^60^ and **9**.^58^ The oxidation changed the geometry at the antimony atom from ψ-tetrahedral to trigonal bipyramidal and shortened the Sb–C bonds (cf. the structures of **G** and the corresponding bis(stiborane), [Fe(η^5^-C_5_H_4_SbPh_2_Cl_2_)_2_]).^16^ The τ_5_ parameter for the stiborane moiety in **12** was 0.86, reflecting that although the Cl–Sb–Cl (axial) angle was close to the ideal 180°, the C–Sb–C angles varied (∼115–128°) for steric reasons. The ferrocene units assumed their regular geometry and were negligibly tilted, but the increased steric bulk of the Sb substituent in **12** was reflected by a more open conformation of the ferrocene unit.

**Table 9:** Selected Distances and Angles for **7** and **12**·0.4CHCl_3_ (in Å and deg)^a^

Parameter	**7** (X = void)	**12**·0.4CHCl_3_ (X = Cl)^b^
Au–Cl	2.2922(7)	2.2864(7)
Au–P	2.2268(7)	2.2286(6)
P–Au–Cl	174.47(3)	175.34(3)
Sb–X1/2	na	2.4356(6)/2.4875(6)
Sb–C1	2.130(2)	2.096(2)
Sb–C23/C29	2.157(3)/2.152(2)	2.107(2)/2.121(3)
C1–Sb–C23/C29	95.03(9)/95.16(9)	117.08(8)/128.10(9)
C23–Sb–C29	95.95(9)	114.71(9)
P–C6	1.787(2)	1.786(2)
P–C11/C17	1.841(2)/1.818(3)	1.811(2)/1.819(2)
C6–P–C11/17	106.2(1)/103.9(1)	108.1(1)/103.7(1)
C11–P–C17	105.4(1)	103.7(1)
Fe–C	2.028(3)–2.064(3)	2.034(2)–2.062(2)
tilt	2.1(2)	1.1(2)
τ	160.8(2)	174.0(2)

a Parameters are defined as for the other compounds discussed in this paper; see the footnote to Table 3. na = not applicable.

b Further data: Cl–Sb–Cl = 179.49(2)°, Cl–Sb–C (range) = 88.57(6)–91.38(6)°.

**Figure 11:**
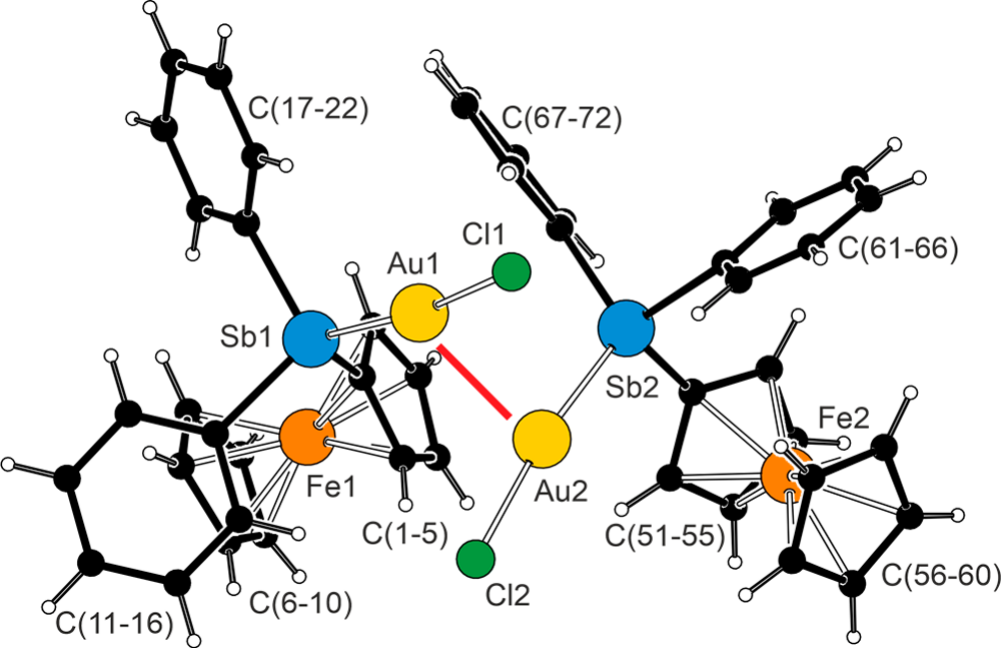
View of the two crystallographically independent molecules of **10**. The Au···Au interaction (Au1···Au2 = 2.9992(5) Å) is indicated by a red line. Selected distances and angles (in Å and deg) for molecule 1 [molecule 2]: Au–Sb 2.4939(4) [2.4945(5)], Au–Cl 2.288(1) [2.3034(9)], Sb–Au–Cl 172.76(3) [169.36(3)].

The molecular structure of stibine complex **10** (Figure 11) was unexceptional in view of the data determined for **7**, **12**·0.4CHCl_3_, and [(μ(Sb,Sb)-**G**)(AuCl)_2_]^16^ (*vide supra*). The prominent feature that differentiated **10** from the reference compounds was the presence of intermolecular aurophilic interactions^61^ between two independent molecules present in the structure (*Z*′ = 2). The Au1···Au2 distance was 2.9992(5) Å, and the interacting P–Au–Cl units were approximately perpendicular to each other (torsion angle Cl1–Au1···Au2–Cl2 was 102.89(4)°). In turn, this suggested that aurophilic interactions can also be responsible for the *multiplication* of the molecules in the asymmetric unit^62^ by linking them into supramolecular molecular arrays that behave as the real repeating unit. This hypothesis was verified through a search in the Cambridge Structural Database^63^ for structures with intramolecular Au···Au distances in the arbitrary 2.7–3.2 Å range *and* with *Z*′ > 1 (i.e., with two or more formula units *per* asymmetric unit), which resulted in approximately 120 hits (duplicate structures were excluded).^64^

### Catalytic Experiments

**Scheme 8:**
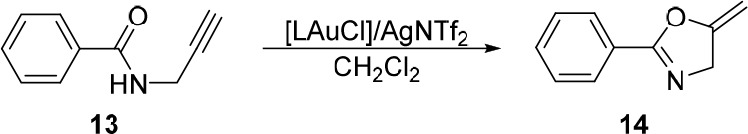
Gold-Catalyzed Cyclization of *N*-Propargylbenzamide (**13**) to Oxazole **14**

Gold(I) complexes **7**, **9**, **10**, and **12**,^65^ activated *in situ* with AgNTf_2_, were applied in Au-mediated cyclization of *N*-propargylbenzamide (**13**) into 4,5-dihydro-5-methylene-2-phenyloxazole (**14**) (Scheme 8).^66^ The reactions were performed with 1 mol % of the gold catalyst in CD_2_Cl_2_ at 25 °C and followed by ^1^H NMR spectroscopy.^67^

**Figure 12:**
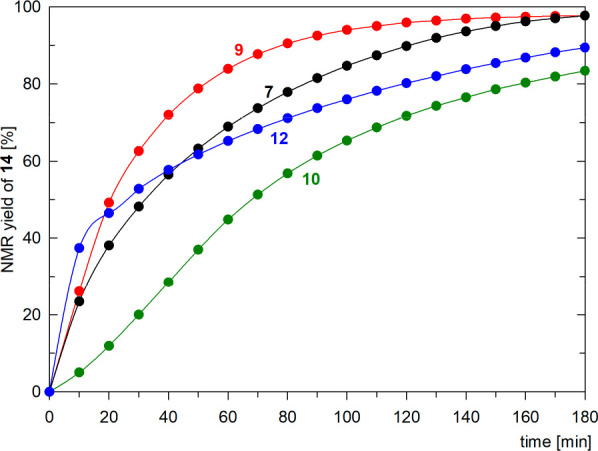
Kinetic profiles for Au-catalyzed cyclization of **13** into oxazole **14** using complexes **7**, **9**, **10**, and **12** as precatalysts. The solid lines are shown only as a guide for an eye.

The cyclization reactions proceeded selectively; no other products were detected in the spectra. The kinetic profiles shown in Figure 12 indicate a superior catalytic performance of complex **9** containing phosphine **3**, which maintained a relatively high activity, comparable to the prototypical catalyst [Au(MeCN)(PPh_3_)][SbF_6_],^67,68^ and resulted in a 97% NMR yield after 3 h (complete conversion was achieved after 6 h). The yield of **14** obtained with phosphine complex **7** was similar but was reached at a slower reaction rate. Notably, oxidation of the stibine moiety, such as in **12**, accelerated the reaction at the initial stages (indicated by a visual comparison of the reaction rates during the first 10–20 min of the reaction), but then, the reaction rate decreased, very likely due to catalyst decomposition. The catalyst with the slowest reaction rate was obtained from complex **10**, which presumably reflected the lower stability of this compound and, consequently, easier catalyst decomposition. Nevertheless, the yield of **14** was 82% after a 3 h reaction time. AgNTf_2_ itself did not catalyze the reaction (N.B.: a further analysis, e.g., an estimation of the initial reaction rates, was not performed, as it could be misleading due to catalyst activation and decomposition).

**Scheme 9:**
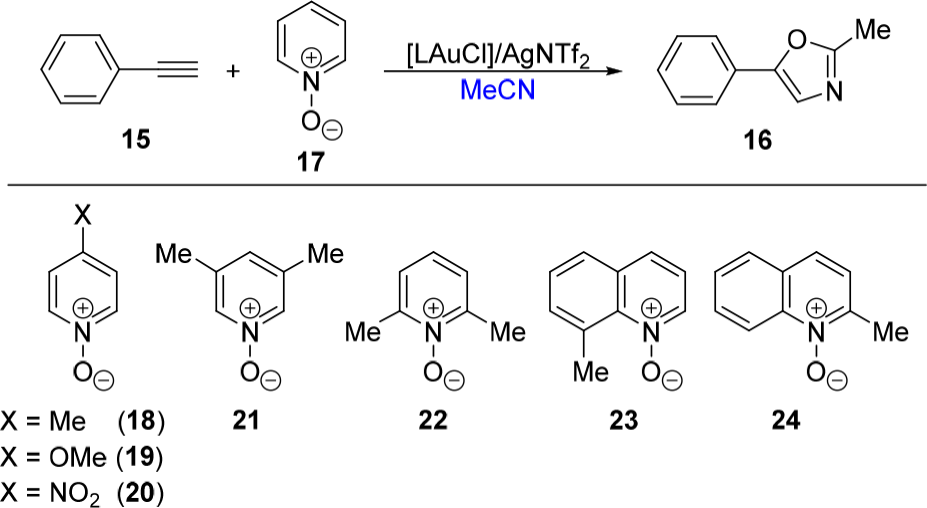
Gold-Catalyzed Oxidative [2 + 2 + 1] Annulation of Ethynylbenzene, Acetonitrile, and Pyridine *N*-Oxides

Next, we investigated the more challenging Au-catalyzed oxidative [2 + 2 + 1] cyclization of ethynylbenzene (**15**) with acetonitrile, used as a solvent, and pyridine *N*-oxides as the oxidants to afford 2-methyl-5-phenyloxazole (**16**; Scheme 9).^69^ The initial screening (Table 10, entries 1–8) using catalysts generated *in situ* from the defined Au(I) complexes (5 mol %), AgNTf_2_ (1 equiv), and pyridine *N*-oxide (**17**), performed at 60 °C for 24 h, showed that only phosphine complexes efficiently mediated this reaction. The yields of **16** achieved with complexes **7** and **9** were 37% and 51%, respectively. A lower yield, 27%, was obtained with compound **12**, whereas no appreciable reaction was observed when the stibine complex **10** was used as the precatalyst. Subsequent experiments focused on complex **7** showed that this compound alone (i.e., without the silver(I) salt) was also active but resulted in a significantly lower yield (4%). Adding 2 or 3 equiv of AgNTf_2_ to complex **12** improved the yield of the cyclization product to approximately 40%.

**Table 10:** Summary of the Catalytic Results for the Au-Catalyzed Formation of Oxazole **16**^a^

entry	Au complex	oxidant	yield of **16** (%)
1	**7**	**17**	37
2	**9**	**17**	51
3	**10**	**17**	0
4	**12**	**17**	27
5^b^	**12**	**17**	4
6	none	**17**	0
7^c^	**12**	**17**	40
8^d^	**12**	**17**	38
9	**7**	**18**	23
10	**7**	**19**	3
11	**7**	**20**	19
12	**7**	**21**	28
13	**7**	**22**	15
14	**7**	**23**	73 (61)^f^
15	**7**	**24**	11
16^e^	**7**	**23**	69 (56)^f^

a Conditions unless specified otherwise: alkyne **17** (*c* = 0.10 M) was added to a mixture of gold complex (5 mol %), AgNTf_2_ (5 mol %), and oxidant (1.3 equiv) in MeCN at 60 °C for 24 h. The yields were determined by ^1^H NMR spectroscopy using anisole (1 equiv) as an internal standard and are an average of two independent runs.

b No silver salt was used.

c 2 equiv of AgNTf_2_ was added.

d 3 equiv of AgNTf_2_ was added.

e Ag[SbF_6_] was used instead of AgNTf_2_.

f Isolated yield in parentheses.

Since the outcome of this catalytic reaction^69^ is known to depend on the *N*-oxide component, we screened several *N*-oxides (Table 10, entries 9–15). The yields achieved with substituted pyridine *N*-oxides **18**-**22** were substantially lower than those achieved with the parent compound **17**, irrespective of the electronic properties of the substituents and steric bulk. An improvement to a 73% yield of **16** was observed when using the sterically encumbered 8-methylquinoline *N*-oxide (**23**), while the reaction in the presence of the isomeric 2-methylquinoline *N*-oxide (**24**) gave only an 11% yield. When AgNTf_2_ was replaced with Ag[SbF_6_] in the reaction with **7** and the best-performing oxidant **23**, the yield of the cyclization product decreased slightly (to 68%), very likely for solubility reasons (AgNTf_2_ is more soluble in organic solvents).

## Conclusion

Reported here are the synthesis, detailed structural characterization, and reactivity studies of 1-(diphenyphosphino)-1′-(diphenylstibino)ferrocene (**1**), which is the first ferrocene-based phosphinostibine ligand, falling halfway between the widely studied 1,1′-bis(diphenylphosphino)ferrocene (dppf)^17^ and 1,1′-bis(diphenylstibino)ferrocene (**G**) reported only recently.^16^ Although compounds combining phosphine and stibine donor groups are not unprecedented, phosphinostibine **1** is unique thanks to the particular combination of steric and electronic properties of its central ferrocene backbone.^14f,70^ As a strong electron donor, the ferrocene moiety increases the electron density at the Sb atom and, thus, decreases its acceptor properties. In addition, the ferrocene scaffold allows mutual reorientation of the functional pnictogen groups attached in positions 1 and 1′ by acting as molecular ball bearing with only low energy barrier. This markedly differentiates compounds **1**, **1E**, **6**, and **6E** (E = O, S, Se) from the previously reported compounds, wherein the P and Sb substituents were typically brought into close proximity by a rigid backbone. Importantly, the functional substituents at the ferrocene unit can be manipulated independently, which allowed the selective synthesis of P(III)/Sb(V), P(V)/Sb(III), and P(V)/Sb(V) derivatives, which were subsequently investigated for possible interactions between the pnictogen groups. While stibines **1** and **1E** virtually lacked donor–acceptor interactions, the more Lewis acidic stiboranes **6** and **6O**, obtained by oxidation of **1** with *o*-chloranil, formed distinct intramolecular interactions of the P → Sb(V) and P=O → Sb(V) type, which were manifested in both the crystal structures and the spectroscopic properties. In contrast, the analogous P=S → Sb(V) and P=Se → Sb(V) interactions in phosphine chalcogenide-stiboranes **6S** and **6Se** were weaker and did not result in the significant stabilization of a particular conformation. DFT calculations were used to investigate the nature of these interactions, and the results showed lone pair donation from phosphine phosphorus (in **6**) or phosphoryl oxygen (in **6O**) to the stiborane Sb atom at the other cyclopentadienyl ring. Although similar in nature, these interactions differed in strength and electrostatic contribution, with that in **6O** being stronger and more ionic due to the strong polarization of the P=O bond.

From another viewpoint, compound **1** can be considered a typical example of a hybrid and potentially hemilabile ligand that forms coordination bonds of different strengths. This is reflected in the stability of the coordination compounds obtained from this ligand and affects the catalytic properties of the obtained complexes. The data collected for complexes containing the soft Au(I) metal ion suggest that stibine coordination does not sufficiently stabilize the active metal species, which is thus prone to decomposition. In turn, this leads to a shorter catalyst lifetime and poorer catalytic performance. A similar observation was previously made for the analogous Pd complexes featuring dppf and **G** as the ligands, among which the phosphine complexes showed better catalytic properties in Suzuki–Miyaura cross-coupling.^16^ From this perspective, compound **1** should be considered a functional phosphine whose P atom acts as the primary coordination site for soft metal ions, while the Sb moiety can be used as a secondary donor moiety or a reactive group whose chemical transformations can be used to control the overall electronic properties and an ability to form structure-directing intra- and intermolecular secondary interactions in the complexes.

## Supporting Information Available

Complete experimental and characterization data for the prepared compounds, a summary of crystallographic parameters, additional structure diagrams, and copies of the NMR spectra (PDF)
Coordinate files for the DFT-optimized structures (XYZ)

## Supplementary Material

ic3c02075_si_001.pdf

ic3c02075_si_002.xyz
