# Supplementary material for: Synthesis, Structure, Reactivity, and Intramolecular Donor–Acceptor Interactions in a Phosphinoferrocene Stibine and Its Corresponding Phosphine Chalcogenides and Stiboranes
Source: Inorg Chem. 2023 Aug 10;62(34):14028–43. doi: 10.1021/acs.inorgchem.3c02075 (PMC10466383; doi:10.1021/acs.inorgchem.3c02075)
Supplement: Supplementary file 1 — ic3c02075_si_001.pdf [file ic3c02075_si_001.pdf]

Supporting Information

Synthesis, Structure, Reactivity, and Intramolecular Donor-Acceptor Interactions in a Phosphinoferrocene Stibine and its Corresponding Phosphine Chalcogenides and Stiboranes

Jiří Schulz, Jakub Antala, David Rezazgui, Ivana Císařová, Petr Štěpnička*

Department of Inorganic Chemistry, Faculty of Science, Charles University, Hlavova 2030, 128 40 Prague, Czech Republic

Contents

Syntheses and characterization

Experimental	S-2
VT NMR and NMR spectra from the experiments with Lewis acids and bases	S-14
X-ray crystallography	S-18
Additional cyclic voltammograms	S-32
DFT calculations	S-35
Copies of the NMR spectra	S-41
References	S-70

Syntheses and characterization

Materials and methods

Unless stated otherwise, all syntheses were performed at ambient temperature under nitrogen using the standard Schlenk techniques.[1] 1-Bromo-1′-(diphenylphosphino)ferrocene (**2**),[2] 1-bromo-1′-(diphenylphosphino)ferrocene–borane (1:1) (**2**·BH_3)[3], chlorodiphenylstibine,[4] (diphenyl-phosphino)ferrocene ($FcPPh_2$ or **3**),[5] [AuCl($FcPPh_2$-κ*P*)] (**9**),[6] [$Au(tht)_2$][SbF_6],[7] and *N*-propargylbenzamide (**13**)[8] were prepared according to literature procedures. Other chemicals were purchased from commercial suppliers and used without additional purification (Sigma–Aldrich, TCI Chemicals, and Alfa–Aesar). Anhydrous tetrahydrofuran, dichloromethane, and diethyl ether were dried using an in-house PureSolv MD5 Solvent Purification System (Innovative Technology, Inc., USA). Acetonitrile was dried over CaH_2 and distilled. Toluene was dried over sodium and distilled. Solvents used during workup and for chromatography were used as received (Lach-Ner, Czech Republic, analytical grade).

NMR spectra were recorded at 25 °C on a Varian UNITY Inova 400 spectrometer (^{1}H, 399.95 MHz; ^{13}C, 100.58 MHz; ^{31}P, 161.90 MHz) unless noted otherwise. Chemical shifts (δ/ppm) are expressed relative to internal tetramethylsilane (^{1}H and ^{13}C NMR) and to external 85% H_3PO_4 (^{31}P). In addition to the standard notation of signal multiplicity,[9] vt and vq are used to denote virtual triplets and quartets arising from the AA′BB′ and AA′BB′X spin systems (A, B = ^{1}H, X = ^{31}P) of the cyclopentadienyl rings C_5H_4Sb and C_5H_4P, respectively (Fc = ferrocenyl, fc = ferrocene-1,1′-diyl). The mass spectra were recorded with a Compact QTOF-MS spectrometer (Bruker Daltonics). The identity of the observed ionic species was corroborated by comparing the theoretical and experimentally determined isotopic patterns. Elemental analyses were performed using a Perkin-Elmer PE 2400 CHN analyzer.

Electrochemical measurements were performed with a multipurpose instrument μAUTOLAB III (Eco Chemie, Netherland) at room temperature using a Metrohm three-electrode cell equipped with a platinum disc working electrode (2 mm diameter), platinum sheet auxiliary electrode, and an Ag/AgCl (3 M KCl) reference electrode. The compounds were dissolved in dry dichloromethane to give a solution containing ca. 1 mM of the analyzed sample and 0.1 M $Bu_4N[PF_6]$ (Sigma–Aldrich, p. a. for electrochemistry). The solutions were deaerated by argon and maintained under an argon blanket during the measurement. Decamethylferrocene (Alfa–Aesar) was added as the internal standard during the final scans, and the redox potentials were converted into the ferrocene/ferrocenium scale by subtracting 0.548 V.[10] Details of structure determination and DFT calculations are given below.

Syntheses

Preparation of 1-(diphenylstibino)-1′-(diphenylphosphino)ferrocene (1) from 2. A flame-dried, three-necked flask equipped with a magnetic stirring bar and an inert gas inlet was charged with 1-bromo-1′-(diphenylphosphino) ferrocene (**2**; 2.25 g, 5.0 mmol) and sealed with a rubber septum. An inert atmosphere was established by three vacuum/nitrogen cycles, and the starting material was dissolved in anhydrous tetrahydrofuran (40 mL). The resulting solution was cooled in a dry ice/ethanol bath to approximately −78 °C before *n*-butyllithium (2.0 mL of 2.5 M solution in hexanes, 5 mmol) was introduced dropwise with continuous cooling. The reaction mixture was stirred for 30 minutes at −78 °C, whereupon it deposited an orange precipitate. Next, a solution of chlorodiphenylstibine in THF (2.02 g, 6.5 mmol in 20 mL), cooled to −78 °C in a separate Schlenk flask, was transferred to the reaction mixture via a cannula, and the resulting mixture was allowed to warm to room temperature overnight. The reaction was terminated by adding saturated aqueous sodium hydrogencarbonate (30 mL) and ethyl acetate (30 mL), and the mixture was transferred into a separatory funnel. The organic phase was separated, washed with brine (50 mL), dried over magnesium sulfate, and evaporated. The crude product was purified by flash column chromatography over silica gel using ethyl acetate-hexane (1:10) as the eluent followed by crystallization from hot heptane. Yield of **1**: 2.44 g (76%), orange crystals.

^{1}H NMR ($CDCl_3$, 399.95 MHz): δ 3.97 (vt, $^3J_{HH}$ = 1.8 Hz, 2 H, CH of fc), 4.02 (vq, $^3J_{HH}$ = 1.9 Hz, 2 H, CH of fc), 4.20 (vt, $^3J_{HH}$ = 1.8 Hz, 2 H, CH of fc), 4.23 (vt, $^3J_{HH}$ = 1.7 Hz, 2 H, CH of fc), 7.23-7.35 (m, 12 H, Ph), 7.40-7.47 (m, 8 H, Ph). ^{13}C{^{1}H} NMR ($CDCl_3$, 100.58 MHz): δ 69.55 (C^{ipso} of C_5H_4Sb), 71.43 (d, J_{PC} = 4 Hz, CH of C_5H_4P), 72.66 (d, J_{PC} = 2 Hz, CH of C_5H_4Sb), 73.33 (d, J_{PC} = 15 Hz, CH of C_5H_4P), 75.34 (CH of C_5H_4Sb), 76.16 (d, $^1J_{PC}$ = 7 Hz, C^{ipso} of C_5H_4P), 128.11 (d, J_{PC} = 7 Hz, CH of PPh_2), 128.40 (CH^{para} of PPh_2/$SbPh_2$), 128.45 (CH^{para} of PPh_2/$SbPh_2$), 128.57 (CH of $SbPh_2$), 133.43 (d, J_{PC} = 20 Hz, CH of PPh_2), 136.12 (CH of $SbPh_2$), 138.54 (C^{ipso} of $SbPh_2$), 138.95 (d, $^1J_{PC}$ = 10 Hz, C^{ipso} of PPh_2). ^{31}P{^{1}H} NMR ($CDCl_3$, 161.90 MHz): δ −16.4 (s). ESI-MS: *m/z* 645.2 ([M + H]$^+$). Anal. Calc. for $C_{34}H_{28}FePSb$ (645.16): C 63.30, H 4.37%. Found: C 63.32, H 4.35%.

Preparation of 1·BH$_3$. The synthesis was performed similarly starting from 1-bromo-1′-(diphenylphosphino)ferrocene–borane (1:1) (**2**·BH_3; 2.31 g, 5.0 mmol). The crude product, obtained after aqueous workup and solvent removal under vacuum, was purified by flash column chromatography on silica gel using diethyl ether–hexane (1:9) as the eluent. The first, yellow band was discarded. The following major band due to the product was collected after gradually changing the eluent to ethyl acetate. The eluate was evaporated under vacuum and the obtained yellow solid

was crystallized from a boiling mixture of ethyl acetate and heptane (1:9, ca. 50 mL) by cooling down to −18 °C. The separated crystalline material was isolated by decantation, washed with cold pentane, and dried under vacuum. Yield of **1**·BH_3: 1.49 g (45%), orange crystals.

^{1}H NMR ($CDCl_3$, 399.95 MHz): δ 0.75-1.75 (very br m, 3 H, BH_3), 3.98 (vt, $^3J_{HH}$ = 1.8 Hz, 2 H, CH of fc), 4.29 (d of vt, J = 1.8, 1.1 Hz, 2 H, CH of fc), 4.32 (vt, $^3J_{HH}$ = 1.7 Hz, 2 H, CH of fc), 4.34 (vq, $^3J_{HH}$ = 1.9 Hz, 2 H, CH of fc), 7.25-7.33 (m, 6 H, Ph), 7.35-7.47 (m, 10 H, Ph), 7.51-7.58 (m, 4 H, Ph). $^{13}C\{^1H\}$ NMR ($CDCl_3$, 100.58 MHz): δ 69.16 (d, $^1J_{PC}$ = 68 Hz, C^{ipso} of C_5H_4P), 70.52 (s, C^{ipso} of C_5H_4Sb), 72.53 (d, J_{PC} = 8 Hz, CH of C_5H_4P), 73.24 (d, J_{PC} = 10 Hz, CH of C_5H_4P), 73.42 (CH of C_5H_4Sb), 75.99 (CH of C_5H_4Sb), 128.44 (d, J_{PC} = 10 Hz, CH of PPh_2), 128.51 (CH^{para} of $SbPh_2$), 128.64 (CH of $SbPh_2$), 130.88 (d, $^4J_{PC}$ = 2 Hz, CH^{para} of PPh_2), 131.27 (d, $^1J_{PC}$ = 59 Hz, C^{ipso} of PPh_2), 132.58 (d, J_{PC} = 10 Hz, CH of PPh_2), 136.08 (CH of $SbPh_2$), 138.41 (C^{ipso} of $SbPh_2$). $^{31}P\{^1H\}$ NMR ($CDCl_3$, 161.90 MHz): δ 16.4 (br d). ESI-MS: *m/z* 681.1 ([M + Na]$^+$). Anal. Calc. for $C_{34}H_{31}BFePSb$ (659.0): C 61.97, H 4.74%. Found: C 61.70, H 4.61%.

Preparation of 1 by deprotection of 1·BH_3. A Schenk flask was charged with 1-(diphenylstibino)-1′-(diphenylphoshino)ferrocene–borane (1:1) (**1**·BH_3; 330 mg, 0.5 mmol) and 1,4-diazabicyclo[2.2.2]octane (224 mg, 2.0 mmol). The reaction vessel was flushed with nitrogen by three vacuum/nitrogen cycles and anhydrous tetrahydrofuran (10 mL) was introduced. The resulting mixture was stirred overnight at room temperature and then evaporated to dryness. The solid residue was purified by flash chromatography over silica gel using ethyl acetate-hexane (1:3) as the eluent. A single orange band was collected and evaporated. The solid residue was crystallized from a diethyl ether solution overlayered with pentane. The obtained crystals were isolated by suction filtration, washed with pentane, and dried under vacuum. Yield of **1**: 232 mg (72%), orange crystals.

Alkylation of 1 with MeI. Compound **1** (645 mg, 1.0 mmol) was dissolved in dichloromethane (10 mL) and iodomethane (125 μL, 2.0 mmol) was added to the solution via a syringe. After the reaction mixture was stirred overnight, the solution was concentrated to ca. 2 mL using a rotary evaporator and mixed with diethyl ether (30 mL). The formed precipitate was allowed to settle overnight, isolated by decantation, and dried under vacuum. Yield of **1**·MeI: 750 mg (73%), yellow powder.

^{1}H NMR ($CDCl_3$, 399.95 MHz): δ 3.05 (d, $^2J_{PH}$ = 13.2 Hz, 3 H, Me), 4.11 (vt, $^3J_{HH}$ = 1.7 Hz, 2 H, CH of fc), 4.53 (vq, J = 1.9 Hz, 2 H, CH of fc), 4.57 (vt, $^3J_{HH}$ = 1.7 Hz, 2 H, CH of fc), 4.60 (vq, J = 1.9 Hz, 2 H, CH of fc), 7.27-7.40 (m, 10 H, Ph), 7.61-7.77 (m, 10 H, Ph). $^{13}C\{^1H\}$ NMR ($CDCl_3$, 100.58 MHz): δ 11.64 (d, $^1J_{PC}$ = 60 Hz, Me), 60.34 (d, $^1J_{PC}$ = 104 Hz, C^{ipso} of C_5H_4P), 72.46 (C^{ipso} of C_5H_4Sb), 73.02 (d, J_{PC} = 13 Hz, CH of C_5H_4P), 74.27 (CH of C_5H_4Sb), 75.17 (d, J_{PC} = 11 Hz, CH of C_5H_4P), 76.83 (CH of C_5H_4Sb), 121.35 (d, $^1J_{PC}$ = 90 Hz, C^{ipso} of PPh_2), 128.92 (CH of $SbPh_2$), 128.94 (CH^{para} of $SbPh_2$), 130.14 (d, J_{PC} = 13 Hz, CH

of PPh_2), 132.55 (d, J_{PC} = 11 Hz, CH of PPh_2), 134.75 (d, $^4J_{PC}$ = 3 Hz, CH^{para} of PPh_2), 136.03 (CH of $SbPh_2$), 137.50 (C^{ipso} of $SbPh_2$). $^{31}P\{^1H\}$ NMR ($CDCl_3$, 161.90 MHz): δ 24.2 (s). ESI-MS: *m/z* 659.3 ($[M - I]^+$). Anal. Calc. for $C_{35}H_{31}FeIPSb$ (660.2): C 53.41, H 3.97%. Found: C 53.28, H 3.86%.

Synthesis of (diphenylstibino)ferrocene (4). A flame-dried Schlenk flask was charged with bromoferrocene (**5**; 1.32 g, 5.0 mmol), flushed with nitrogen, and sealed with a rubber septum. The starting material was dissolved in anhydrous tetrahydrofuran (20 mL) and the solution was cooled in a dry ice-ethanol bath. *n*-Butyllithium (2.0 mL of 2.5 M solution in hexanes, 5 mmol) was added dropwise and the resulting mixture was stirred with continuous cooling for 30 minutes, depositing an orange precipitate. Next, a solution of chlorodiphenylstibine in tetrahydrofuran (2.02 g, 6.5 mmol in 20 mL) precooled to −78 °C was added via a cannula, the cooling bath was removed, and the reaction mixture was stirred for another 1 h. The reaction was terminated by adding saturated aqueous sodium hydrogencarbonate (20 mL) and ethyl acetate (20 mL). The organic phase was separated, washed with brine (20 mL), dried over magnesium sulfate, and evaporated under vacuum. The solid residue was purified by flash chromatography on silica gel using ethyl acetate-hexane (1:30) as the eluent. Subsequent evaporation and crystallization from hot heptane produced **4** as an orange crystalline solid. Yield: 1.38 g (60%), orange crystals.

^1H NMR ($CDCl_3$, 399.95 MHz): δ 4.05 (vt, $^3J_{HH}$ = 1.8 Hz, 2 H, C_5H_4), 4.07 (s, 5 H, C_5H_5), 4.35 (vt, $^3J_{HH}$ = 1.7 Hz, 2 H, C_5H_4), 7.29-7.34 (m, 6 H, $SbPh_2$), 7.46-7.52 (m, 4 H, $SbPh_2$). $^{13}C\{^1H\}$ NMR ($CDCl_3$, 100.58 MHz): δ 68.70 (C^{ipso} of C_5H_4Sb), 68.53 (C_5H_5), 71.12 (CH of C_5H_4), 74.39 (CH of C_5H_4), 128.40 (CH^{para} of $SbPh_2$), 128.59 (CH of $SbPh_2$), 136.17 (CH of $SbPh_2$), 138.66 (C^{ipso} of $SbPh_2$). ESI-MS: *m/z* 460.1 (M^+). Anal. Calc. for $C_{22}H_{19}FeSb$ (460.99): C 57.32, H 4.15%. Found: C 57.04, H 3.94%.

Preparation of 1-bromo-1′-(diphenylphosphinoyl)ferrocene (2O). In the air, a round bottom flask was charged with 1-bromo-1´-(diphenylphosphino)ferrocene (**2**; 450.3 mg, 1.0 mmol). The solid educt was dissolved in reagent-grade acetone (60 mL), and the resulting yellow solution was cooled to 0 °C in an ice bath before hydrogen peroxide (0.5 mL of 30% solution, ca. 4.9 mmol) was introduced. The reaction mixture was stirred at 0 °C for 10 min and then allowed to warm to room temperature while stirring for an additional 20 min. The reaction was quenched with saturated aqueous $Na_2S_2O_3$ (2 mL) and concentrated under reduced pressure. The residue was partitioned between ethyl acetate (50 mL) and deionized water (50 mL). The organic layer was separated, washed with brine (50 mL), dried over magnesium sulfate, and evaporated under vacuum. The yellow-orange solid residue was dissolved in dichloromethane (ca. 5 mL) and crystallized by adding boiling heptane (25 ml). The solution was heated further in a fume hood to boil off the most of dichloromethane. The solid, which separated after slow cooling to −18 °C, was decanted, washed with

pentane (3× 3 mL), and dried under vacuum. A second crop was obtained by concentrating the mother liquor under reduced pressure to ca. 5 mL and isolated similarly. Combined yield: 435.5 mg (94%), orange crystals.

^{1}H NMR ($CDCl_3$, 399.95 MHz): δ 4.23 (vt, *J*´ = 1.9 Hz, 2 H, CH of fc), 4.40-4.45 (m, 4 H, CH of fc), 4.51 (vq, *J*´ = 1.8 Hz, 2 H, CH of fc), 7.41-7.45 (m, 6 H, PPh_2), 7.63-7.72 (m, 4 H, PPh_2). ^{31}P{^{1}H} NMR ($CDCl_3$, 161.90 MHz): δ 28.0 (s). The data match those reported in the literature.[2]

Preparation of 1-(diphenylstibino)-1′-(diphenylphosphinoyl)ferrocene (1O). A two-necked, oven-dried flask (10 mL), equipped with a magnetic stirring bar, a septum, and a nitrogen inlet, was charged with **2O** (139.5 mg, 0.30 mmol) and thoroughly purged with nitrogen. Dry THF (2 mL) was introduced to dissolve the solid educt, and the solution was cooled to −78 °C in a dry ice/ethanol bath. A solution of *n*-BuLi in hexanes (0.13 mL of 2.5 M solution, 0.33 mmol) was introduced dropwise, causing the color of the mixture to turn red. After stirring for 30 min at −78 °C, a pre-cooled solution of chlorodiphenylstibine (112.1 mg, 0.36 mmol) in THF (1.5 mL) was added slowly to the reaction mixture, which was kept stirred at −78 °C for another 1 h and then at room temperature overnight. The reaction mixture was evaporated under reduced pressure, the resulting oil was dissolved in ethyl acetate (15 ml) and the solution was transferred into a separatory funnel. The organic phase was washed successively with deionized water (15 mL) and brine (15 mL), dried over magnesium sulfate, filtered, and evaporated under reduced pressure. The yellow-brown oily residue was purified by column chromatography over silica gel using ethyl acetate as the eluent. The first yellow band was collected and evaporated under reduced pressure. The residue was triturated with hexane to give an orange solid, which was isolated by decantation and dried under vacuum. Yield: 99.7 mg (50%), yellow powdery solid. Single crystals were obtained from a hot ethyl acetate/hexane mixture by slow cooling to room temperature.

^{1}H NMR ($CDCl_3$, 399.95 MHz): δ 4.05 (vt, *J*´ = 1.7 Hz, 2 H, CH of fc), 4.27 (vq, *J*´ = 1.8 Hz, 2 H, CH of fc), 4.30 (vq, *J*´ = 1.8 Hz, 2 H, CH of fc), 4.50 (vt, *J*´ = 1.7 Hz, 2 H, CH of fc), 7.24-7.33 (m, 6 H, Ph), 7.37-7.44 (m, 8 H, Ph), 7.45-7.52 (m, 2 H, Ph), 7.59-7.67 (m, 4 H, Ph). ^{13}C{^{1}H} NMR ($CDCl_3$, 100.58 MHz): δ 70.54 (C^{ipso} of C_5H_4Sb), 72.34 (d, J_{CP} = 10 Hz, CH of C_5H_4P), 72.67 (d, J_{CP} = 13 Hz, CH of C_5H_4P), 73.20 (d, $^{1}J_{CP}$ = 116 Hz, C^{ipso} of C_5H_4P; this signal partly overlaps with the signal at δ_C 72.67), 73.24 (CH of C_5H_4Sb), 75.82 (CH of C_5H_4Sb), 128.20 (d, $^{2}J_{CP}$ = 12 Hz, CH^{ortho} of PPh_2), 128.48 (CH of $SbPh_2$), 128.61 (CH of $SbPh_2$), 131.38 (d, $^{3}J_{CP}$ = 10 Hz, CH^{meta} of PPh_2), 131.50 (d, $^{4}J_{CP}$ = 3 Hz, CH^{para} of PPh_2), 134.19 (d, $^{1}J_{CP}$ = 106 Hz, C^{ipso} of PPh_2), 137.07 (CH of $SbPh_2$), 138.37 (C^{ipso} of $SbPh_2$). ^{31}P{^{1}H} NMR ($CDCl_3$, 161.90 MHz): δ 29.3 (s). ESI-MS: *m*/*z* 583.0 ($[M - Ph]^+$), 661.0 ($[M + H]^+$), 683.0 ($[M + Na]^+$). Anal. Calc. for $C_{34}H_{28}FeOPSb$ (661.2) C 61.76, H 4.27%. Found C 61.56, H 4.07%.

Synthesis of $Ph_2P(S)fcSbPh_2$ (1S). A two-necked, oven-dried flask (50 ml), equipped with a magnetic stirring bar, a septum, and a reflux condenser with a nitrogen inlet, was charged with **1** (162.4 mg, 0.25 mmol) and sulfur (8.07 mg, 0.25 mmol) and purged with nitrogen. Anhydrous toluene (8 mL) was introduced and the resulting mixture was heated in an oil bath under reflux for 1 h 40 min. After cooling, the orange solution was evaporated under reduced pressure, leaving an orange oil, which was purified by chromatography over silica gel, using dichloromethane-methanol (20:1) as the eluent. The first, major yellow band was collected and evaporated under reduced pressure. The product was crystallized from boiling heptane (4 mL) by slow cooling to room temperature. The separated solid was decanted, washed with pentane, and dried under vacuum. Yield: 157.1 mg (92%), orange crystals. A single crystal was selected from the reaction batch.

^{1}H NMR ($CDCl_3$, 399.95 MHz): δ 4.04 (vt, J' = 1.8 Hz, 2 H, CH of fc), 4.28 (vq, J' = 1.9 Hz, 2 H, CH of fc), 4.36 (vq, J' = 1.9 Hz, 2 H, CH of fc), 4.38 (vt, J' = 1.8 Hz, 2 H, CH of fc), 7.26-7.33 (m, 6 H, Ph), 7.36-7.49 (m, 10 H, Ph), 7.64-7.71 (m, 4 H, Ph). $^{13}C\{^1H\}$ NMR ($CDCl_3$, 100.58 MHz): δ 70.59 (C^{ipso} of C_5H_4Sb), 72.43 (d, $^3J_{CP}$ = 10 Hz, CH of C_5H_4P), 73.36 (d, $^2J_{CP}$ = 13 Hz, CH of C_5H_4P), 73.62 (CH of C_5H_4Sb), 75.32 (d, $^1J_{CP}$ = 98 Hz, C^{ipso} of C_5H_4P), 76.20 (CH of C_5H_4Sb), 128.20 (d, $^2J_{CP}$ = 13 Hz, CH^{ortho} of PPh_2), 128.46 (CH^{para} of $SbPh_2$), 128.59 (CH of $SbPh_2$), 131.18 (d, $^4J_{CP}$ = 3 Hz, CH^{para} of PPh_2), 131.57 (d, $^3J_{CP}$ = 11 Hz, CH^{meta} of PPh_2), 134.49 (d, $^1J_{CP}$ = 87 Hz, C^{ipso} of PPh_2), 136.10 (CH of $SbPh_2$), 138.52 (C^{ipso} of $SbPh_2$). $^{31}P\{^1H\}$ NMR ($CDCl_3$, 161.90 MHz): δ 41.9 (s). ESI-MS: m/z 677.0 ($[M + H]^+$), 699.0 ($[M + Na]^+$). Anal. Calc. for $C_{34}H_{28}FePSSb$ (677.2): C 60.30, H 4.17%. Found: C 60.54, H 4.41%.

Preparation of $Ph_2P(Se)fcSbPh_2$ (1Se). A dry, two-necked reaction flask (25 mL), equipped with a magnetic stirring bar, a septum, and a reflux condenser with a nitrogen inlet, was charged with **1** (64.4 mg, 0.10 mmol) and grey selenium (7.88 mg, 0.10 mmol), and thoroughly purged with nitrogen. Anhydrous toluene (3 mL) was introduced and the mixture was heated at reflux in an oil bath for 1 h. After cooling to ambient temperature, the orange solution was evaporated under reduced pressure, and the resulting orange oil was purified by column chromatography over silica gel, using dichloromethane-methanol (20:1) as the eluent. The first, major yellow band was collected and evaporated under reduced pressure. The product was further crystallized by dissolving in dichloromethane (1 mL) and adding boiling heptane (3 mL). The mixture was heated further to boil off the most of dichloromethane and cooled to room temperature, whereupon the product separated. The solid was decanted, washed with pentane, and dried under vacuum. Yield of **1Se**: 61.5 mg (83%), yellow microcrystalline solid. Single crystals were obtained from dichloromethane-hexane (liquid phase diffusion).

^1H NMR ($CDCl_3$, 399.95 MHz): δ 4.05 (vt, *J*′ = 1.7 Hz, 2 H, CH of fc), 4.29 (vq, *J*′ = 1.7 Hz, 2 H, CH of fc), 4.36-4.40 (m, 4 H, CH of fc), 7.26-7.31 (m, 6 H, Ph), 7.35-7.48 (m, 10 H, Ph), 7.63-7.71 (m, 4 H, Ph). $^{13}C\{^1H\}$ NMR ($CDCl_3$, 100.58 MHz): δ 70.69 (C^{ipso} of C_5H_4Sb), 72.55 (d, $^3J_{CP}$ = 10 Hz, CH of C_5H_4P), 73.79 (CH of C_5H_4Sb), 73.80 (d, $^2J_{CP}$ = 13 Hz, CH of C_5H_4P), 74.29 (d, $^1J_{CP}$ = 89 Hz, C^{ipso} of C_5H_4P), 76.38 (CH of C_5H_4Sb), 128.21 (d, $^2J_{CP}$ = 12 Hz, CH^{ortho} of PPh_2), 128.47 (CH^{para} of $SbPh_2$), 128.60 (CH of $SbPh_2$), 131.23 (d, $^4J_{CP}$ = 3 Hz, CH^{para} of PPh_2), 132.01 (d, $^3J_{CP}$ = 11 Hz, CH^{meta} of PPh_2), 133.39 (d, $^1J_{CP}$ = 78 Hz, C^{ipso} of PPh_2), 136.11 (CH of $SbPh_2$), 138.54 (C^{ipso} of $SbPh_2$). $^{31}P\{^1H\}$ NMR ($CDCl_3$, 161.90 MHz): δ 32.1 (s with ^{77}Se satellites, $^1J_{PSe}$ = 735 Hz). ESI-MS: *m*/*z* 724.9 ([M + H]$^+$), 746.9 ([M + Na]$^+$). Anal. Calc. for $C_{34}H_{28}FePSbSe$ (724.2) C 56.39, H 3.90%. Found C 56.32, H 3.66%.

Preparation of $Ph_2PfcSbPh_2(O_2C_6Cl_4)$ (6). A solution of *o*-chloranil (24.6 mg, 0.10 mmol) in dichloromethane (1 mL) was added dropwise to **1** (64.5 mg, 0.10 mmol) dissolved in the same solvent (2 mL), and the resulting orange solution was stirred for 1 h. The reaction mixture was diluted with dichloromethane (5 mL) and evaporated with silica gel. The pre-adsorbed product was loaded on a silica gel column (Interchim puriFlash, 30 µm, 25 g) using a solid loader and purified using a Büchi Reveleris X2 automatic chromatograph with UV detection and ethyl acetate-hexane (1:9) as the eluent (flow rate 25 mL min^{-1}). The second yellow band was collected, evaporated, and dried under vacuum. Yield of **6**: 16.7 mg (18%), pale yellow powder. Single crystals were obtained from ethyl acetate-hexane (liquid-phase diffusion).

^1H NMR ($CDCl_3$, 399.95 MHz): δ 4.25 (vq, *J*′ = 1.8 Hz, 2 H, CH of fc), 4.32 (vt, *J*′ = 1.8 Hz, 2 H, CH of fc), 4.40 (vt, *J*′ = 1.8 Hz, 2 H, CH of fc), 4.77 (vt, *J*′ = 1.8 Hz, 2 H, CH of fc), 7.08-7.15 (m, 4 H, Ph), 7.15-7.23 (m, 4 H, Ph), 7.24-7.30 (m, 2 H, Ph), 7.39-7.50 (m, 6 H, Ph), 7.77-7.84 (m, 4 H, Ph). $^{13}C\{^1H\}$ NMR ($CDCl_3$, 100.58 MHz): δ 71.91 (d, $^3J_{CP}$ = 4 Hz, CH of C_5H_4P), 72.24 (d, $^3J_{CP}$ = 2 Hz, CH of C_5H_4Sb), 73.94 (d, $^2J_{CP}$ = 8 Hz, C^{ipso} of C_5H_4Sb), 74.69 (d, $^2J_{CP}$ = 11 Hz, CH of C_5H_4P), 75.64 (CH of C_5H_4Sb), 85.74 (d, $^1J_{CP}$ = 21 Hz, C^{ipso} of C_5H_4P), 116.21 (CCl), 119.88 (CCl), 128.11 (d, $^3J_{CP}$ = 7 Hz, CH^{meta} of PPh_2), 129.04 (br s, CH^{para} of PPh_2), 129.27 (CH of $SbPh_2$), 130.99 (CH^{para} of $SbPh_2$), 133.00 (d, $^2J_{CP}$ = 17 Hz, CH^{ortho} of PPh_2), 134.39 (CH of $SbPh_2$), 135.38 (d, $^2J_{CP}$ = 2 Hz, C^{ipso} of $SbPh_2$), 139.90 (d, $^1J_{CP}$ = 11 Hz, C^{ipso} of PPh_2), 144.92 (CO). $^{31}P\{^1H\}$ NMR ($CDCl_3$, 161.90 MHz): δ −9.7 (s). ESI-MS: *m*/*z* 675.1 ([M – $O_2C_6Cl_4$ + OCH_3]$^+$), 707.0 ([M – $O_2C_6Cl_4$ + 2OCH_3 + H]$^+$), 890.9 ([M + H]$^+$). Anal. Calc. For $C_{40}H_{28}Cl_4FeO_2PSb·0.7C_6H_{14}$ (951.4): C 55.80, H 4.01%. Found: C 55.69, H 3.97% (the compound crystallizes as a hexane solvate, which was verified by structure determination).

Synthesis of $Ph_2P(O)fcSbPh_2(O_2C_6Cl_4)$ (6O). A solution of *o*-chloranil (24.6 mg, 0.10 mmol) in dichloromethane (1 mL) was added dropwise to a solution of **1** (32.3 mg, 0.05 mmol) in wet dichloromethane (1 mL + 2 drops of deionized water) and the resulting yellow-orange mixture was

rapidly stirred for 5 h. The solution was then dried over magnesium sulfate and filtered through a PTFE syringe filter (0.45 μm porosity). The filtrate was evaporated under reduced pressure. The solid residue was taken up with chloroform (ca. 3 mL), and the solution was concentrated to ca. 1 mL and layered with hexane (6 mL) in a test tube. Crystallization by liquid-phase diffusion over several days produced orange crystals, which were decanted, washed with hexane (3× 2 mL), and dried under vacuum. Yield of solvated **6O**: 34.1 mg (65%), orange crystals. Single crystals were grown from chloroform/hexane.

^{1}H NMR (CD_2Cl_2, 399.95 MHz): δ 4.45 (vt, *J*′ = 1.8 Hz, 2 H, CH of fc), 4.62 (br s, 2 H, CH of fc), 4.74 (br s, 2H, CH of fc), 7.21-7.29 (m, 4 H, Ph), 7.31-7.43 (m, 10 H, Ph), 7.43-7.49 (m, 2 H, Ph), 7.79 (br s, 4 H, Ph). One signal due to CH of fc was not observed due to extensive broadening. ^{13}C{^{1}H} NMR (CD_2Cl_2, 100.58 MHz): δ 69.34 (d, $^{1}J_{CP}$ = 110 Hz, C^{ipso} of C_5H_4P), 72.27 (CH of C_5H_4Sb), 73.77 (br s, CH of C_5H_4P), 76.01 (br s, CH of C_5H_4Sb), 76.73 (d, J_{CP} = 15 Hz, CH of C_5H_4P), 87.09 (C^{ipso} of C_5H_4Sb), 116.26 (CCl), 118.93 (CCl), 128.57 (d, $^{2}J_{CP}$ = 13 Hz, CH^{ortho} of PPh_2), 128.77 (CH of $SbPh_2$), 129.94 (CH^{para} of $SbPh_2$), 131.68 (d, $^{3}J_{CP}$ = 11 Hz, CH^{meta} of PPh_2), 132.63 (d, $^{4}J_{CP}$ = 3 Hz, CH^{para} of PPh_2), 134.39 (br s, CH of $SbPh_2$), 145.67 (C^{ipso} of $SbPh_2$), 146.62 (CO). The signal due to C^{ipso} of PPh_2 was not observed. ^{31}P{^{1}H} NMR (CD_2Cl_2, 161.90 MHz): δ 39.1 (s). ESI-MS: *m*/*z* 906.9 ([M + H]$^{+}$), 928.9 ([M + Na]$^{+}$). Anal. Calc. For $C_{40}H_{28}Cl_4FeO_3PSb·1.2CHCl_3$ (1050.3): C 47.12, H 2.80%. Found: C 47.28, H 2.82% (the compound crystallizes as a chloroform solvate).

Preparation of $Ph_2P(S)fcSbPh_2(O_2C_6Cl_4)$ (6S). A solution of *o*-chloranil (24.6 mg, 0.10 mmol) in dichloromethane (2 mL) was added dropwise to **1S** (67.7 mg, 0.10 mmol) dissolved in the same solvent (3 mL). The resulting orange mixture was stirred for 1 h and evaporated under reduced pressure. The orange solid was purified by column chromatography over silica gel, using dichloromethane-methanol (20:1) as the eluent. The major yellow band was collected and evaporated to give a glassy residue, which was redissolved in dichloromethane (2 mL) and diluted with hexane (4 mL). The solution was evaporated and the solid residue obtained was dried under vacuum. Yield of **6S**: 64.6 mg (67%), orange powdery solid.

^{1}H NMR ($CDCl_3$, 399.95 MHz): δ 4.37-4.43 (m, 6 H, CH of fc), 4.61 (vt, *J*′ = 1.8 Hz, 2 H, CH of fc), 7.32-7.40 (m, 4 H, Ph), 7.42-7.61 (m, 12 H, Ph), 7.76-7.82 (m, 4 H, Ph). ^{13}C{^{1}H} NMR ($CDCl_3$, 100.58 MHz): δ 72.73 (d, J_{CP} = 10 Hz, CH of C_5H_4P), 74.28 (d, J_{CP} = 13 Hz, CH of C_5H_4P), 74.50 (C^{ipso} of C_5H_4Sb), 75.05 (CH of C_5H_4Sb), 75.73 (CH of C_5H_4Sb), 76.25 (d, $^{1}J_{CP}$ = 96 Hz, C^{ipso} of C_5H_4P; the signal is partly obscured by the solvent resonance), 116.36 (CCl), 120.36 (CCl), 128.30 (d, $^{2}J_{CP}$ = 13 Hz, CH^{ortho} of PPh_2), 129.42 (CH of $SbPh_2$), 131.47 (d, $^{3}J_{CP}$ = 11 Hz, CH^{meta} of PPh_2), 131.48 (d, $^{4}J_{CP}$ = 3 Hz, CH^{para} of PPh_2), 131.61 (CH^{para} of $SbPh_2$), 133.50 (d, $^{1}J_{CP}$ = 87 Hz, C^{ipso} of PPh_2), 134.74 (CH of $SbPh_2$), 137.55 (C^{ipso}

of $SbPh_2$), 144.47 (CO). $^{31}P\{^1H\}$ NMR ($CDCl_3$, 161.90 MHz): δ 41.4 (s). ESI-MS: *m/z* 922.9 ($[M + H]^+$), 944.9 ($[M + Na]^+$). Anal. Calc. for $C_{40}H_{28}Cl_4FeO_2PSSb{\cdot}0.4C_6H_{14}$ (957.6): C 53.18, H 3.54%. Found: C 53.23, H 3.49%.

Preparation of $Ph_2P(Se)fcSbPh_2(O_2C_6Cl_4)$ (6Se). A solution of *o*-chloranil (12.8 mg, 0.052 mmol) in dichloromethane (1 mL) was added dropwise to a solution of **1Se** (37.0 mg, 0.050 mmol) in the same solvent (1 mL) and the solution was stirred at room temperature for 1 h. The reaction mixture was evaporated under reduced pressure and the yellow solid residue was dissolved in dichloromethane (1 mL) and mixed with boiling heptane (9 mL). The resulting solution was shortly heated to boil off the most dichloromethane and then slowly cooled to room temperature. The deposited product was decanted, washed with pentane (3× 2 mL), and dried under vacuum. Yield of **6Se**: 32.8 mg (66%), yellow powdery solid.

^1H NMR ($CDCl_3$, 399.95 MHz): δ 4.38-4.43 (m, 4 H, CH of fc), 4.45 (vq, J' = 1.7 Hz, 2 H, CH of fc), 4.61 (vt, J' = 1.7 Hz, 2 H, CH of fc), 7.31-7.40 (m, 4 H, Ph), 7.41-7.60 (m, 12 H, Ph), 7.76-7.82 (m, 4 H, Ph). $^{13}C\{^1H\}$ NMR ($CDCl_3$, 100.58 MHz): δ 72.90 (d, J_{CP} = 10 Hz, CH of C_5H_4P), 74.73 (d, J_{CP} = 12 Hz, CH of C_5H_4P), 75.11 (CH of C_5H_4Sb), 75.26 (C^{ipso} of C_5H_4Sb), 75.48 (d, $^1J_{CP}$ = 87 Hz, CH of C_5H_4P), 75.85 (CH of C_5H_4Sb), 116.43 (CCl), 120.37 (CCl), 128.35 (d, $^2J_{CP}$ = 12 Hz, CH^{ortho} of PPh_2), 129.41 (CH of $SbPh_2$), 131.57 (CH of $SbPh_2$), 131.58 (d, $^4J_{CP}$ = 3 Hz, CH^{para} of PPh_2; the resonance partly overlaps with the signal at δ_C 131.57), 131.94 (d, $^3J_{CP}$ = 11 Hz, CH^{meta} of PPh_2), 132.38 (d, $^1J_{CP}$ = 78 Hz, C^{ipso} of PPh_2; the signal partly overlaps with another signal at δ_C 131.94), 134.72 (CH of $SbPh_2$), 137.89 (C^{ipso} of $SbPh_2$), 144.49 (CO). $^{31}P\{^1H\}$ NMR ($CDCl_3$, 161.90 MHz): δ 31.8 (s with ^{77}Se satellites, $^1J_{PSe}$ = 726 Hz). ESI-MS: *m/z* 755.0 ($[M - O_2C_6Cl_4 + OCH_3]^+$). Anal. Calc. for $C_{40}H_{28}Cl_4FeO_2PSbSe{\cdot}0.2C_7H_{16}$ (990.1) C 50.23, H 3.18%. Found C 50.30, H 3.20%.

Preparation of [AuCl(1-*κP*)] (7). Solid [AuCl(SMe_2)] (88 mg, 0.30 mmol) was added to a solution of **1** (194 mg, 0.30 mmol) in dichloromethane (10 mL). The resulting mixture was stirred for 30 min and then evaporated to dryness. The solid residue was taken up with chloroform (2 mL) and filtered through a PTFE syringe filter (0.45 μm porosity). Cold diethyl ether (3 mL) and pentane (9 mL) were slowly added to the filtrate. Subsequent crystallization at 4 °C over several days afforded orange crystals, which were isolated by decantation, washed with diethyl ether, and dried under vacuum. Yield of **6**: 191 mg (73%), orange crystals.

^1H NMR ($CDCl_3$, 399.95 MHz): δ 4.07 (vt, $^3J_{HH}$ = 1.7 Hz, 2 H, CH of fc), 4.27 (d of vt, J = 3.0, 1.9 Hz, 2 H, CH of fc), 4.34 (d of vt, J = 1.2, 1.7 Hz, 2 H, CH of fc), 4.43 (vt, $^3J_{HH}$ = 1.8 Hz, 2 H, CH of fc), 7.26-7.34 (m, 7 H, Ph), 7.38-7.44 (m, 7 H, Ph), 7.45-7.57 (m, 6 H, Ph). $^{13}C\{^1H\}$ NMR ($CDCl_3$, 100.58 MHz): δ 69.27 (d, $^1J_{PC}$ = 73 Hz, C^{ipso} of C_5H_4P), 70.95 (C^{ipso} of C_5H_4Sb), 73.13 (d, J_{PC} = 9 Hz, CH of C_5H_4P), 73.63

(CH of C_5H_4Sb), 73.92 (d, J_{PC} = 14 Hz, CH of C_5H_4P), 76.42 (CH of C_5H_4Sb), 128.60 (CH^{para} of $SbPh_2$), 128.69 (CH of $SbPh_2$), 128.90 (d, J_{PC} = 12 Hz, CH of PPh_2), 130.79 (d, $^1J_{PC}$ = 64 Hz, C^{ipso} of PPh_2), 131.63 (d, $^4J_{PC}$ = 2 Hz, CH^{para} of PPh_2), 133.50 (d, J_{PC} = 14 Hz, CH of PPh_2), 136.09 (CH of $SbPh_2$), 138.25 (C^{ipso} of $SbPh_2$). $^{31}P\{^1H\}$ NMR($CDCl_3$, 161.90 MHz): δ 28.9 (s). ESI-MS: *m/z* 841.0 ($[M - Cl]^+$). Anal. Calc. for $C_{34}H_{28}AuClFePSb$ (877.6): C 46.53, H 3.22%. Found: C 46.32, H 3.00%.

Synthesis of [(μ(P,Sb)-1)(AuCl)$_2$] (8). Complex **8** was prepared similarly to its mononuclear analog **7**. Thus, solid [AuCl(SMe_2)] (59 mg, 0.20 mmol) was added to a solution of **1** (65 mg, 0.10 mmol) in dichloromethane (5 mL). The reaction mixture was stirred for 30 min in the dark and then evaporated to dryness. The solid residue was taken up with dichloromethane (2 mL) and the solution was mixed with a mixture of cold diethyl ether (10 mL) and pentane (20 mL). The precipitate formed was isolated by decantation, washed with pentane, and dried under vacuum. Yield: 100 mg (90 %), yellow powder. Note: the complex gradually darkens when stored at room temperature and decomposes in solution (especially in halogenated solvents).

^1H NMR (CD_2Cl_2, 400.13 MHz): δ 4.32 (vt, $^3J_{HH}$ = 1.8 Hz, 2 H, CH of fc), 4.38 (d of vt, J = 3.0, 1.9 Hz, 2 H, CH of fc), 4.54 (d of vt, J = 1.1, 1.9 Hz, 2 H, CH of fc), 4.59 (vt, $^3J_{HH}$ = 1.8 Hz, 2 H, CH of fc), 7.43–7.65 (m, 20 H, Ph). ^{13}C NMR could not be recorded due to sample decomposition. $^{31}P\{^1H\}$ NMR (CD_2Cl_2, 161.97 MHz): 27.4 (s). ESI+ MS: *m/z* 1132.9 ($[M + Na]^+$), 1075.0 ($[M - Cl]^+$). Anal. Calc. for $C_{34}H_{28}Au_2Cl_2FePSb$ (1110.0): C 36.79, H 2.54%. Found: C 37.12, H 2.56%.

Preparation of [AuCl(FcSbPh$_2$-κ*Sb*)] (10). Solid [AuCl(SMe_2)] (147 mg, 0.50 mmol) was added to a solution of **4** (231 mg, 0.50 mmol) in dichloromethane (10 mL) and the resulting mixture was stirred for 30 min and then evaporated to dryness. The solid residue was dissolved in dichloromethane (1 mL). The solution was filtered through a 0.45 μm PTFE syringe filter and the filtrate was added to diethyl ether (10 mL). Crystallization at 4 °C overnight afforded an orange microcrystalline solid, which was isolated by decantation, washed with diethyl ether, and dried under vacuum. Yield of **8**: 304 mg (87%), orange crystals. *Note:* the complex darkens when stored at ambient temperature.

^1H NMR ($CDCl_3$, 399.95 MHz): δ 4.15 (s, 5 H, C_5H_5), 4.30 (br s, 2 H, C_5H_4), 4.54 (br s, 2 H, C_5H_4), 7.44-7.54 (m, 6 H, Ph), 7.60-7.64 (m, 4 H, Ph). $^{13}C\{^1H\}$ NMR ($CDCl_3$, 100.58 MHz): δ 62.09 (C^{ipso} of C_5H_4), 69.53 (C_5H_5), 72.28 (CH of C_5H_4), 73.73 (CH of C_5H_4), 129.77 (CH of $SbPh_2$), 130.00 (C^{ipso} of $SbPh_2$), 131.25 (CH^{para} of $SbPh_2$), 135.40 (CH of PPh_2). ESI-MS: *m/z* 1118.9 ($[Au(\mathbf{4})_2]^+$), 717.0 ($[M - Cl + Me_2CO]^+$). Anal. Calc. for $C_{22}H_{19}AuClFeSb$ (693.4): C 38.11, H 2.76%. Found: C 38.18, H 2.46%.

Synthesis of [AuCl(6-κ*P*)] (11). A solution of *o*-chloranil (7.4 mg, 0.030 mmol) in dichloromethane (1 mL) was added dropwise to complex **7** (26.3 mg, 0.030 mmol) dissolved in the

same solvent (1 mL). The resulting yellow solution was stirred in the dark for 30 min and then evaporated under reduced pressure. The yellow solid residue was dissolved in dichloromethane (0.5 mL) and the solution was slowly added to hexane (10 mL) under stirring. The separated solid was filtered off, washed with hexane (3× 2 mL,) and dried under reduced pressure. Yield: 26.5 mg (78 %), pale yellow powdery solid, which retains residual hexane.

^{1}H NMR ($CDCl_3$, 399.95 MHz): δ 4.23 (m, 2 H, CH of fc), 4.37 (vt, 2 H, J'= 1.9 Hz, CH of fc), 4.46 (m, 2 H, CH of fc), 4.74 (vt, 2 H, J'= 1.9 Hz, CH of fc), 7.37-7.60 (m, 16 H, Ph), 7.73-7.80 (m, 4 H, Ph). ^{13}C{^{1}H} NMR ($CDCl_3$, 100.58 MHz): δ 71.50 (d, $^{1}J_{CP}$ = 72 Hz, C^{ipso} of C_5H_4P), 72.18 (C^{ipso} of C_5H_4Sb), 73.37 (d, J_{CP} = 9 Hz, CH of C_5H_4P), 74.35 (d, J_{CP} = 14 Hz, CH of C_5H_4P), 75.52 (CH of C_5H_4Sb), 76.03 (CH of C_5H_4Sb), 116.38 (CCl), 120.62 (CCl), 129.03 (d, $^{3}J_{CP}$ = 12 Hz, CH^{meta} of PPh_2), 129.66 (CH of $SbPh_2$), 129.88 (d, $^{1}J_{CP}$ = 64 Hz, C^{ipso} of PPh_2), 131.95 (d, $^{4}J_{CP}$ = 3 Hz, CH^{para} of PPh_2), 132.07 (CH of $SbPh_2$), 133.39 (d, $^{2}J_{CP}$ = 14 Hz, CH^{ortho} of PPh_2), 134.77 (CH of $SbPh_2$), 136.19 (C^{ipso} of $SbPh_2$), 144.26 (CO). ^{31}P{^{1}H} NMR ($CDCl_3$, 161.90 MHz): δ 28.3 (s). ESI-MS: *m/z* 907.4 ([M − $O_2C_6Cl_4$ + CH_3O]$^+$), 1145.4 ([M + Na]$^+$). Anal. Calc. for $C_{40}H_{28}AuCl_5FeO_2PSb·0.1C_6H_{14}$ (1134.7) C 43.07, H 2.62%. Found C 43.20, H 2.53%.

Synthesis of [AuCl($Ph_2PfcSbCl_2Ph_2$-κ*P*)] (12). A reaction flask equipped with a magnetic stirring bar was charged with **1** (207.8 mg, 0.32 mmol) and dry dichloromethane (10 mL). Solid [AuCl(Me_2S)] (94.9 mg, 0.32 mmol) was added to the ligand solution, using an additional 5 mL of dichloromethane to wash the solid educt. The reaction mixture was stirred for 30 minutes at room temperature before it was cooled on ice. Neat $SOCl_2$ (57 mg, 0.48 mmol) was added and the resulting mixture was stirred for 1 h at room temperature and evaporated under vacuum. The residue was dissolved in dichloromethane (2 mL) and added to pentane (10 mL). The separated solid was isolated by decantation and dried in a nitrogen stream. Yield of **12**: 291 mg, (96%), yellow-brown powder. Single crystals were obtained by diffusion of pentane into the chloroform solution of the compound.

^{1}H NMR ($CDCl_3$, 399.95 MHz): δ 4.40 (m, 2 H, CH of fc), 4.46 (m, 2 H, CH of fc), 4.74 (vt, J' = 2.0 Hz, 2 H, CH of fc), 5.04 (vt, J' = 2.0 Hz, 2 H, CH of fc), 7.58-7.42 (m, 16 H, Ph), 8.22-8.16 (m, 4 H, Ph). ^{13}C{^{1}H} NMR ($CDCl_3$, 100.58 MHz): δ 71.58 (d, $^{1}J_{CP}$ = 72 Hz, C^{ipso} of C_5H_4P), 74.69 (d, J_{CP} = 13 Hz, CH of C_5H_4P), 74.84 (d, J_{CP} = 9 Hz, CH of C_5H_4P), 75.18 (CH of C_5H_4Sb), 76.37 (CH of C_5H_4Sb), 80.20 (C^{ipso} of C_5H_4Sb), 129.07 (d, $^{3}J_{CP}$ = 12 Hz, CH^{ortho} of PPh_2), 129.47 (CH^{para} of $SbPh_2$), 130.15 (d, $^{1}J_{CP}$ = 64 Hz, C^{ipso} of PPh_2), 131.74 (CH^{ortho} of $SbPh_2$), 131.92 (d, $^{4}J_{CP}$ = 3 Hz, CH^{para} of PPh_2), 133.52 (d, $^{2}J_{CP}$ = 14 Hz, CH^{meta} of PPh_2), 133.94 (CH^{meta} of $SbPh_2$), 140.53 (C^{ipso} of $SbPh_2$). ^{31}P{^{1}H} NMR ($CDCl_3$, 161.90 MHz): δ 28.2 (s). ESI-MS: *m/z* 911.3 ([M − Cl]$^+$). Anal. Calc. for $C_{34}H_{28}AuCl_3FePSb·0.25CHCl_3$ (978.3): C 42.05, H 2.91%. Found C 42.21, H 2.62% (crystallized sample).

Gold-catalyzed cyclization of *N*-propargylbenzamide. A small glass vial was charged with the respective gold complex (2.0 μmol), *N*-propargylbenzamide (**13**; 31.8 mg, 0.20 mmol), and CD_2Cl_2 (0.50 mL). The mixture was transferred into an NMR tube and a stock solution of silver(I) bis(triflimide) in CD_2Cl_2 was added (0.336 mL of 5.98 mM $AgNTf_2$, 2.0 μmol) into the NMR tube. The sample was inserted into the NMR spectrometer and the ^{1}H NMR spectra were recorded every 10 minutes for the next 3 hours at 25 °C. The yield was determined by integration of the signals due to the CH_2 group of the starting material **13** (δ_H 4.25 ppm) and the CH_2 group of product **14** (δ_H 4.65 ppm).

Analytical data for **13**. ^{1}H NMR ($CDCl_3$, 300 MHz): δ 2.28 (t, *J* = 2.5 Hz, 1 H), 4.25 (dd, *J* = 2.5 Hz, 5.1 Hz, 2 H), 6.35-6.50 (bs, 1 H), 7.40-7.46 (m, 2 H), 7.48-7.53 (m, 1 H), 7.79 (d, *J* = 7.0 Hz, 2 H). Analytical data for **14**. ^{1}H NMR ($CDCl_3$, 300 MHz): δ 4.36 (q, *J* = 2.7 Hz, 1 H), 4.65 (t, *J* = 2.9 Hz, 2 H), 4.81 (q, *J* = 3.0 Hz, 1 H) 7.40-7.47 (m, 2 H) 7.47-7.54 (m, 1 H), 7.94-8.01 (m, 2 H). The data match those reported in the literature.[11]

Gold-catalyzed oxidative cyclization of ethynylbenzene (15) and MeCN. A Schlenk flask was loaded with a stirring bar, the respective gold complex (12.5 μmol), silver bis(triflimide) (4.9 mg, 12.5 μmol), and the appropriate N-oxide (0.325 mmol). The solid educts were dissolved in dry acetonitrile (2.5 mL), and phenylacetylene (25.5 mg, 0.25 mmol) was added. The flask was sealed and the reaction mixture was left stirring at 60 °C for 24 h. Next, anisole (27.0 mg, 0.25 mmol) was added as an internal standard, and an aliquot (0.1 mL) was withdrawn via a syringe and filtered through a PTFE filter into an NMR tube. The filtrate was diluted with $CDCl_3$ and analyzed by ^{1}H NMR spectroscopy. The yield was determined by integration of the signals due to the anisole CH_3O group (δ_H 3.75 ppm) and to the CH_3 group of the cyclization product **16** (δ_H 2.53 ppm). If appropriate, the reaction mixture was evaporated under vacuum and the residue was purified using column chromatography over silica gel with ethyl acetate-hexane (17:83) as the eluent.

Analytical data for 2-methyl-5-phenyloxazole (**16**). ^{1}H NMR ($CDCl_3$, 300 MHz): δ 2.53 (s, 3 H), 7.20 (m, 2 H), 7.30 (m, 1 H), 7.40 (m, 2 H), 7.60 (m, 2 H).[7]

Catalytic experiment with 1 and $AgNTf_2$. A Schlenk flask was charged with a stirring bar, **1** (8.1 mg, 12.5 μmol), silver bis(triflimide) (4.9 mg, 12.5 μmol), and pyridine-N-oxide (**17**; 30.9 mg, 0.325 mmol). The mixture was dissolved in dry acetonitrile, 2.5 ml, and phenylacetylene (0.25 mmol) was added. The flask was sealed and the reaction mixture was left stirring at 60 °C for 24 h and analyzed as described above.

VT NMR spectra for 6O and NMR from the experiments with 6 and 6O and Lewis acids and bases

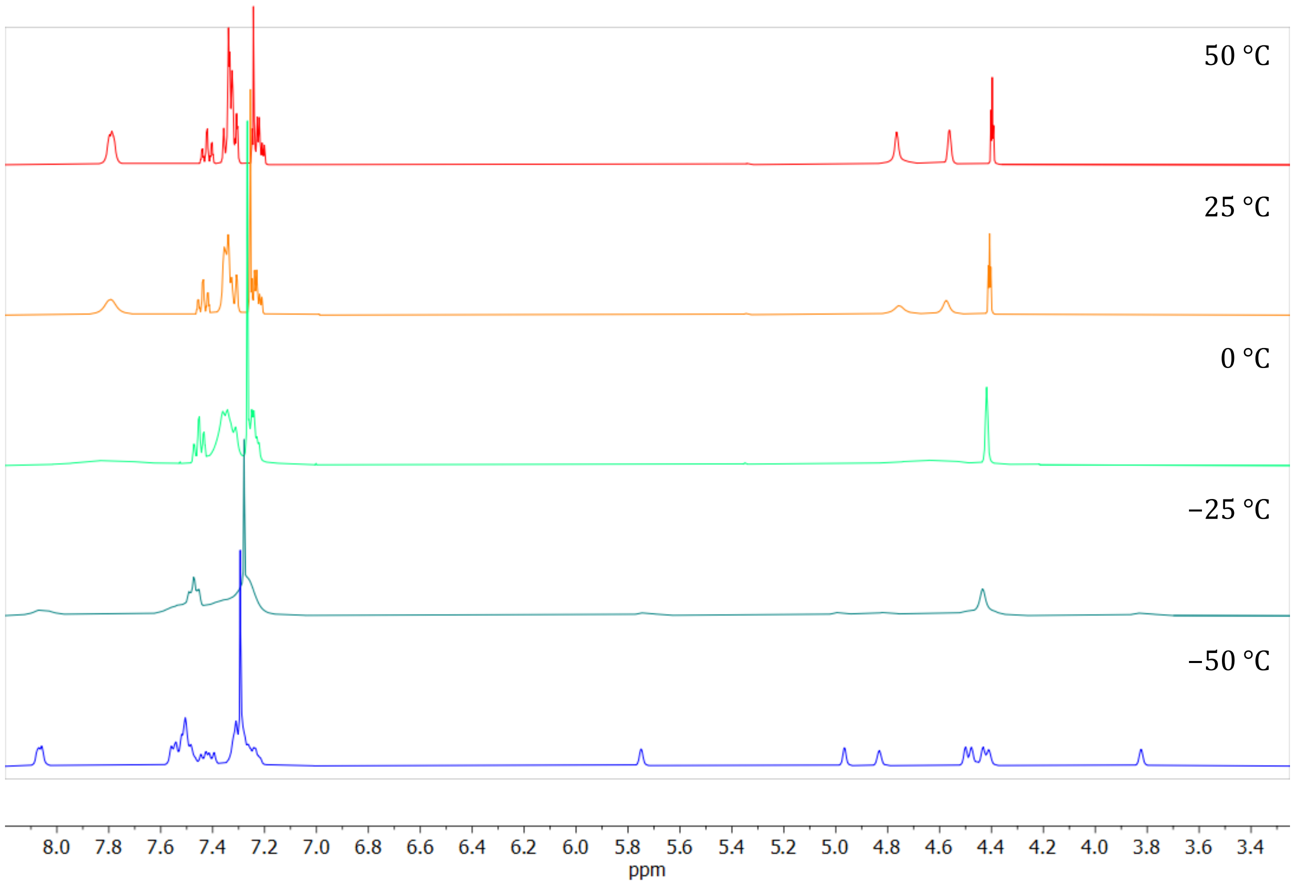

Figure S1. VT ^{1}H NMR (400 MHz, $CDCl_3$) spectra of **6O**.

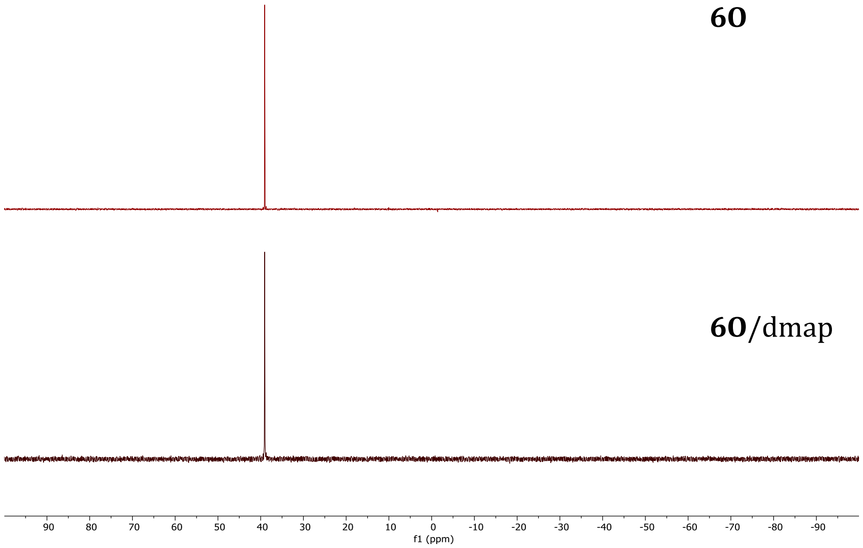

Figure S2. $^{31}P\{^{1}H\}$ NMR spectra (162 MHz, $CDCl_3$, 25 °C) of **6O** and a **6O**/4-(dimethylamino)pyridine (dmap) mixture (1:1).

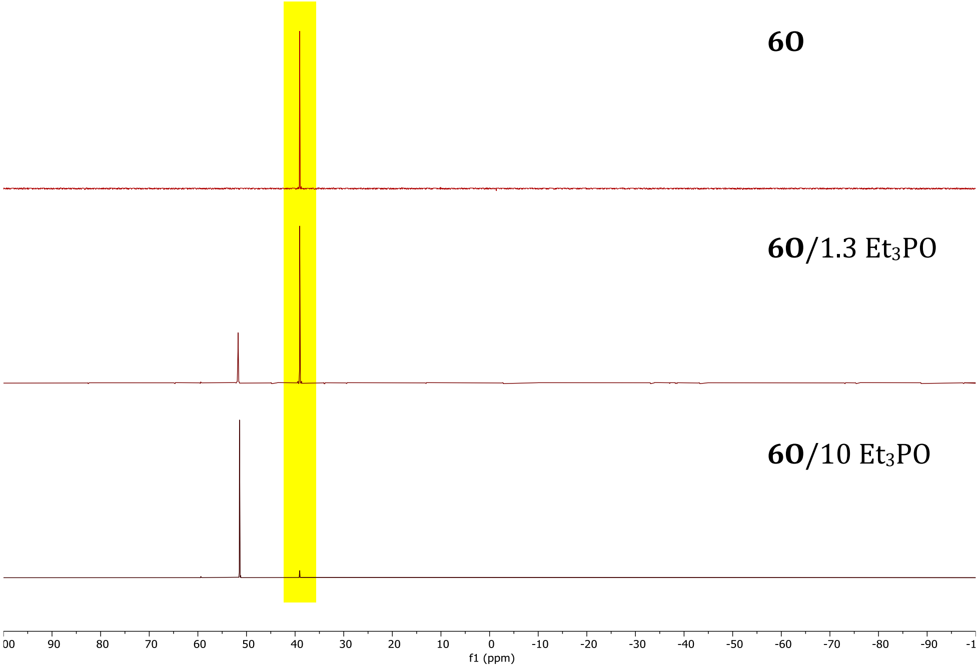

Figure S3. $^{31}P\{^{1}H\}$ NMR spectra (162 MHz, $CDCl_3$, 25 °C) of **6O** and a **6O**/Et_3PO mixtures. The signals due to **6O** are highlighted with a yellow box.

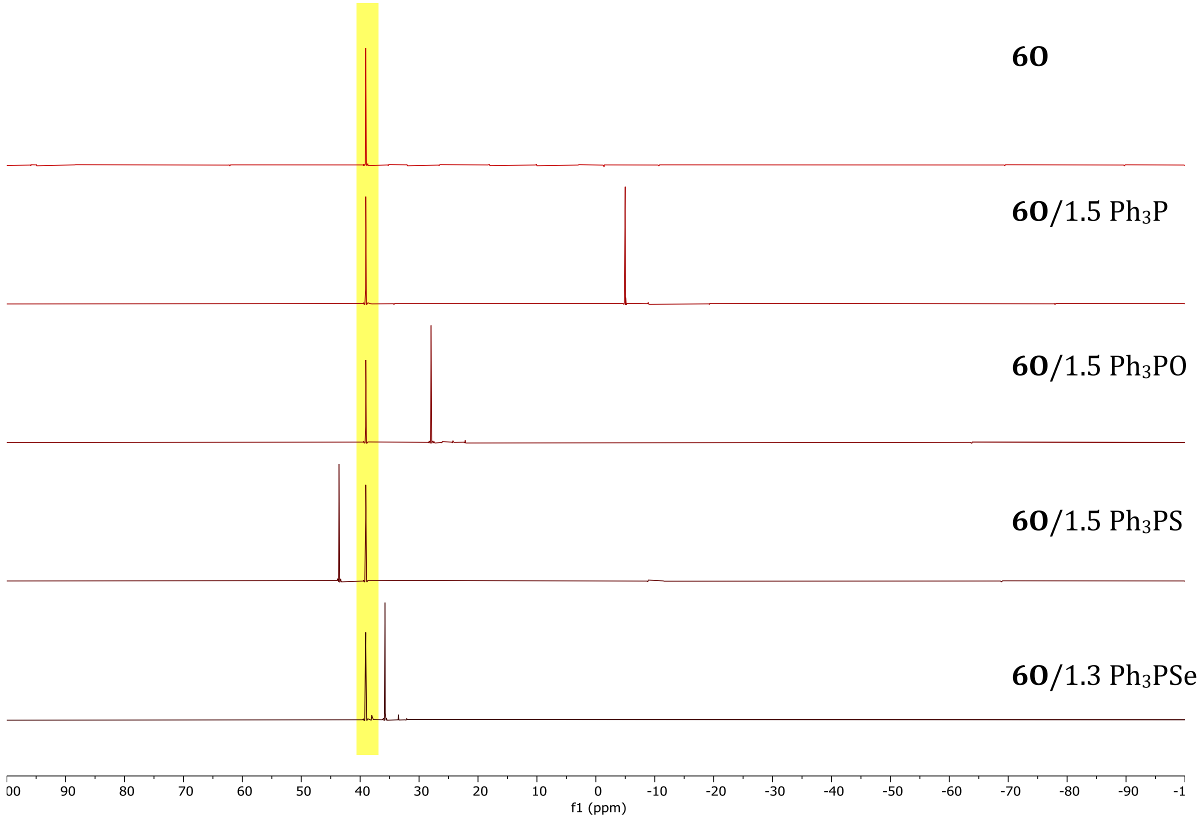

Figure S4. $^{31}P\{^{1}H\}$ NMR spectra (162 MHz, $CDCl_3$, 25 °C) of **6O** and **6O**/Ph_3PE (1:1.5; E = void, O, S, and Se) mixtures. The signals due to **6O** are highlighted with a yellow box.

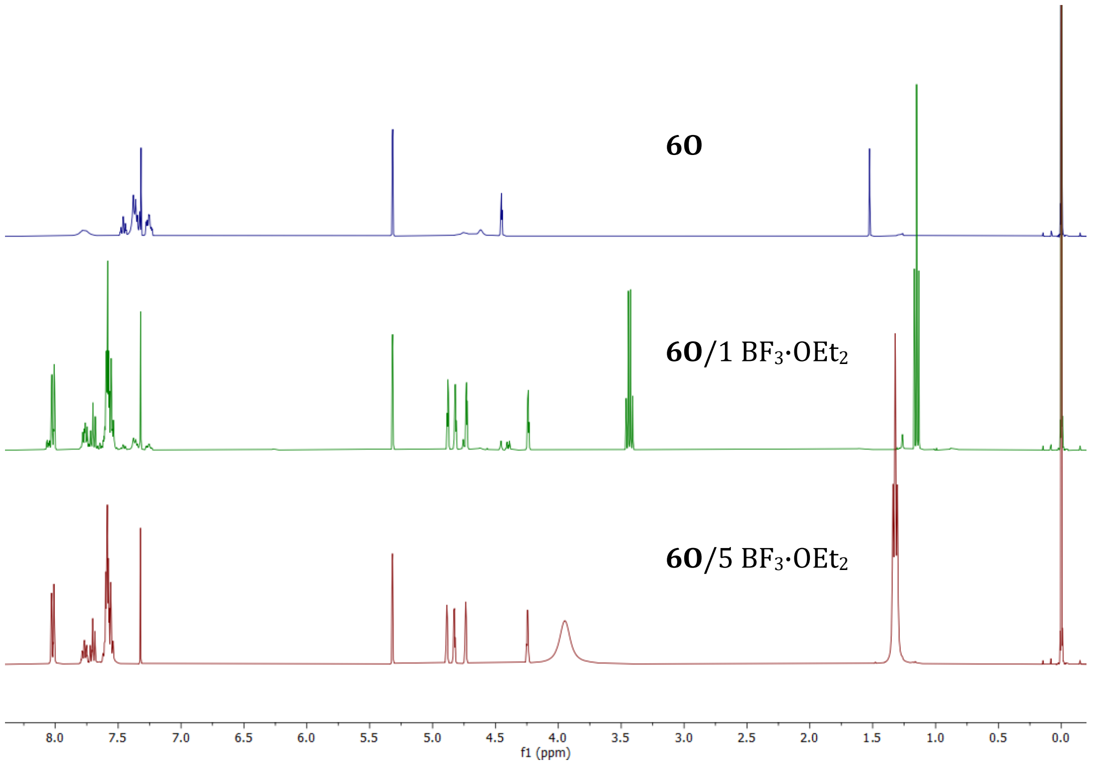

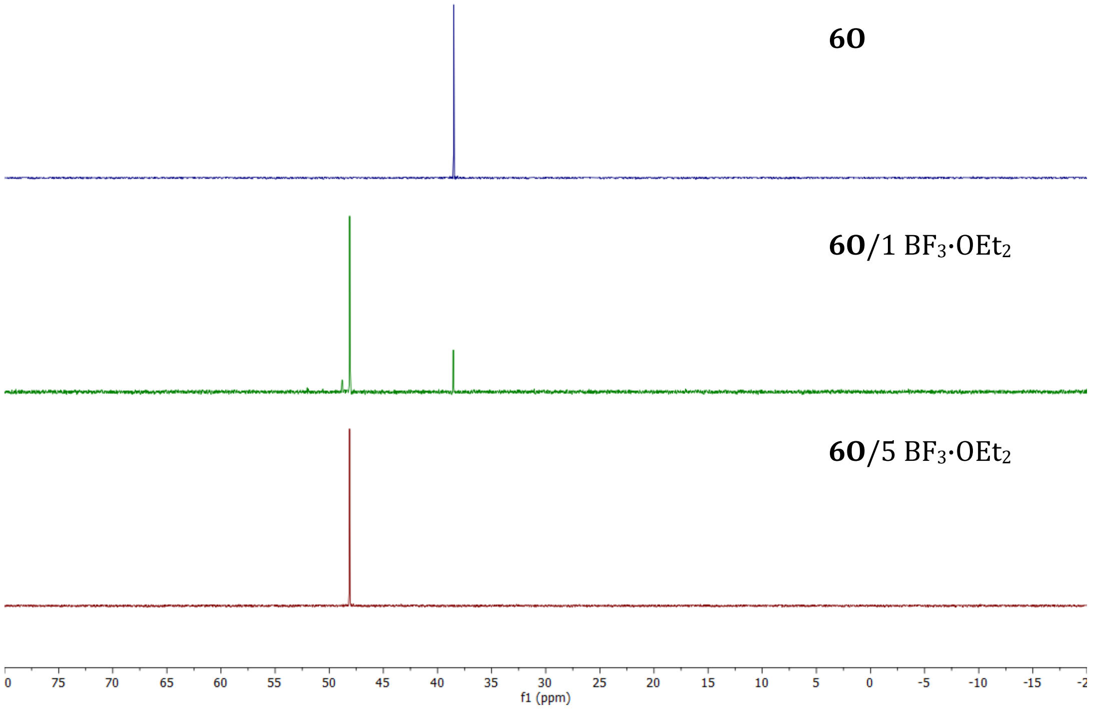

Figure S5. ^{1}H (400 MHz, $CDCl_3$, 25 °C) and $^{31}P\{^{1}H\}$ NMR spectra (162 MHz, $CDCl_3$, 25 °C) of **6O** and **6O**/$BF_3\cdot OEt_2$ mixtures.

X-ray crystallography

Full-set diffraction data (±*h*±*k*±*l*, 2θ ≤ 26° or 27.5°) were collected with Bruker D8 VENTURE Kappa Duo equipped with a Cryostream Cooler using Mo Kα radiation (λ = 0.71073 Å). The structures were solved by direct methods using SHELXT-2018[12] and subsequently refined with SHELXL-2017.[13] All nonhydrogen atoms were refined with anisotropic displacement parameters. Hydrogen atoms were included in their calculated positions and refined as riding atoms with U_{iso}(H) set to 1.5U_{eq}(C) (methyl groups) or 1.2U_{eq} (aromatic CH and CH_2 groups) and the standard parameters implemented in SHELXL. Structure-specific details are as follows.

Compound **1·MeI**·CH_2Cl_2 crystallized with two structurally independent moieties in the unit cell. One of the independent iodide anions in the structure was partly substituted by a chloride anion as a result of halide exchange during crystallization from a dichloromethane/diethyl ether mixture. The refined occupancies at the site were 29:71 (I:Cl).

The crystals of **1S** repeatedly obtained from hot heptane suffered from racemic twining (monoclinic space group *Cc*). The refined contributions of the two enantiomeric domains in the selected specimen were 84:16. In addition, the structure was disordered so that the Sb and P=S moieties alternated in their positions without affecting the overall shape determined by the position of the central ferrocene unit and the phenyl groups. The refined occupancies of the two contributing orientations were 88:12.

Compound **6** crystallized as a hexane solvate **6**·C_6H_{14} with the solvent molecules disordered around the crystallographic inversion centers. Their contribution to the overall scattering was numerically eliminated using PLATON SQUEEZE.[14] In total, 97 electrons were removed per unit cell, which matches the expected value (2× C_6H_{14} corresponds to 100 electrons). In addition, one of the phenyl substituents at the Sb atom, C(17-22), was disordered and had to be modeled over two positions (refined relative occupancies were 67:33). Complex **12** also crystallized with disordered chloroform molecules in the structure, which was also treated as a diffuse electron density. The number of removed electrons (49 per unit cell) corresponded to 0.4 $CHCl_3$ per the complex molecule

Selected crystallographic data and structure refinement parameters are presented in Table S1. All geometric data and structural diagrams were obtained using a recent version of the PLATON program.[15] The numerical values were rounded to one decimal place with respect to the estimated standard deviations. Complete crystallographic data were deposited with the Cambridge Crystallographic Data Centre (CCDC) and can be accessed via www.ccdc.cam.ac.uk/structures. The CCDC deposition numbers are given in Table S1.

Table S1. Selected crystallographic data and structure refinement parameters.[a]

Compound	**1·BH₃**	**1S**	**1Se**
Formula	$C_{34}H_{31}BFePSb$	$C_{34}H_{28}FePSSb$	$C_{34}H_{28}FePSbSe$
M	658.97	677.19	724.09
Crystal system	monoclinic	monoclinic	monoclinic
Space group	$P2_1/n$ (no. 14)	*Cc* (no. 9)[c]	$P2_1/n$ (no. 14)
T [K]	150(2)	150(2)	120(2)
a [Å]	8.8234(3)	23.7721(8)	8.775(1)
b [Å]	30.002(1)	10.9124(4)	29.728(4)
c [Å]	11.1603(4)	11.8031(5)	11.240(2)
α [°]	90	90	90
β [°]	101.691(1)	109.579(1)	101.282(4)
γ [°]	90	90	90
V [Å]^3	2893.1(2)	2884.8(2)	2875.4(7)
Z	4	4	4
μ(Mo Kα) [mm^{-1}]	1.512	1.589	2.788
F(000)	1328	1360	1432
Diffrns collected	58269	108166	41577
Independent diffrns	6932	6620	6586
Observed[a] diffrns	6136	6386	6318
R_{int}[b] [%]	3.56	4.22	2.43
No. of parameters	343	372	343
R[b] obsd diffrns [%]	3.77	2.02	2.05
R, *wR*[b] all data [%]	4.46, 8.29	2.30, 4.85	2.15, 4.86
Δρ [e Å^{-3}]	0.99, −1.32	0.29, −0.34	1.17, −0.75
CCDC no.	2268692	2268694	2268695

[a] Diffractions with $I > 2\sigma(I)$. [b] Definitions: $R_{int} = \Sigma|F_o^2 - F_o^2(\text{mean})|/\Sigma F_o^2$, where $F_o^2(\text{mean})$ is the average intensity of symmetry-equivalent diffractions. $R = \Sigma||F_o| - |F_c||/\Sigma|F_o|$, $wR = [\Sigma\{w(F_o^2 - F_c^2)^2\}/\Sigma w(F_o^2)^2]^{1/2}$. [c] The compound crystallized as a racemic twin.

Table S1 continued

Compound	**1·MeI**·CH_2Cl_2	**6**·C_6H_{14}	**6O**·$CHCl_3$
Formula	$C_{71}H_{64}Cl_{2.71}Fe_2I_{1.29}P_2Sb_2$	$C_{46}H_{42}Cl_4FeO_2PSb$	$C_{41}H_{29}Cl_7FeO_3PSb$
M	1594.13	977.16	1026.36
Crystal system	monoclinic	triclinic	monoclinic
Space group	$P2_1/c$ (no. 14)	P−1 (no. 2)	$P2_1/c$ (no. 14)
T [K]	150(2)	150(2)	150(2)
a [Å]	23.895(1)	9.7730(7)	10.0867(2)
b [Å]	17.8570(9)	14.913(1)	31.4423(8)
c [Å]	15.4622(7)	16.372(1)	12.7800(3)
α [°]	90	63.673(2)	90
β [°]	102.381(2)	74.297(2)	102.718(1)
γ [°]	90	86.290(2)	90
V [Å]^3	6444.3(5)	2054.5(3)	3953.7(2)
Z	4	2	4
μ(Mo Kα) [mm^{-1}]	2.092	1.348	1.604
F(000)	3154	988	2040
Diffrns collected	100164	131881	75662
Independent diffrns	12674	9451	9068
Observed[a] diffrns	10926	9106	8616
R_{int}[b] [%]	3.76	2.46	3.04
No. of parameters	736	467	487
R[b] obsd diffrns [%]	2.32	2.00	2.00
R, wR[b] all data [%]	3.17, 5.32	2.10, 4.99	2.17, 4.81
Δρ [e Å^{-3}]	0.58, −1.04	1.53, −0.66	0.75, −0.66
CCDC no.	2268693	2268697	2268698

Table S1 continued

Compound	**4**	**7**	**9**
Formula	$C_{22}H_{19}FeSb$	$C_{34}H_{28}AuClFePSb$	$C_{22}H_{19}AuClFeSb$
M	460.97	877.55	693.39
Crystal system	triclinic	triclinic	monoclinic
Space group	*P*−1 (no. 2)	*P*−1 (no. 2)	$P2_1/c$ (no. 14)
T [K]	150(2)	150(2)	150(2)
a [Å]	8.3066(4)	8.8680(4)	18.9350(5)
b [Å]	9.4414(5)	10.7447(5)	11.1542(4)
c [Å]	12.7308(6)	16.1903(7)	19.7459(6)
α [°]	97.003(2)	78.552(1)	90
β [°]	101.571(1)	85.228(1)	100.544(1)
γ [°]	110.927(1)	78.643(1)	90
V $[Å]^3$	892.94(8)	1480.8(1)	4100.0(2)
Z	2	2	8
μ(Mo Kα) $[mm^{-1}]$	2.324	6.497	9.279
F(000)	456	840	2592
Diffrns collected	29300	26048	65632
Independent diffrns	4101	6774	9444
Observed[a] diffrns	3871	6480	8521
R_{int}[b] [%]	2.25	3.19	3.33
No. of parameters	217	353	469
R[b] obsd diffrns [%]	1.69	1.80	1.90
R, *wR*[b] all data [%]	1.89, 4.18	1.95, 4.35	2.35, 4.13
Δρ [e $Å^{-3}$]	0.37, −0.55	1.26. −0.85	1.27, −1.80
CCDC no.	2268696	2268699	2268701

Table S1 continued

Compound	**12**·0.4$CHCl_3$
Formula	$C_{34.4}H_{28.4}AuCl_{4.2}FePSb$
M	996.20
Crystal system	triclinic
Space group	*P*−1 (no. 2)
T [K]	120(2)
a [Å]	9.0517(5)
b [Å]	13.8857(8)
c [Å]	14.8382(8)
α [°]	97.457(2)
β [°]	107.744(2)
γ [°]	104.096(2)
V [Å]^3	1680.3(2)
Z	2
μ(Mo Kα) [mm^{-1}]	5.985
F(000)	954
Diffrns collected	159717
Independent diffrns	7706
Observed[a] diffrns	7556
R_{int}[b] [%]	2.85
No. of parameters	370
R[b] obsd diffrns [%]	1.52
R, *wR*[b] all data [%]	1.57, 3.73
Δρ [e $Å^{-3}$]	2.03, −0.49
CCDC no.	2268700

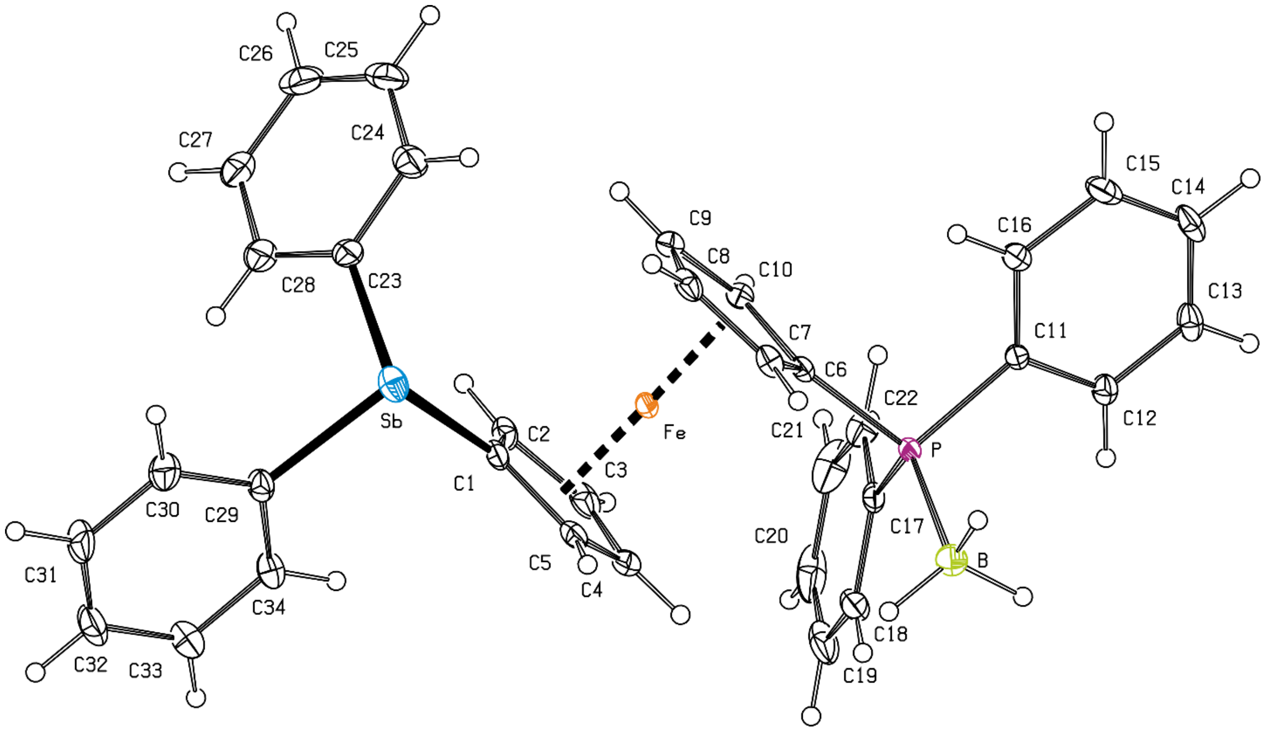

Figure S6. PLATON plot of the molecular structure of **1**·BH_3 showing 30% probability ellipsoids.

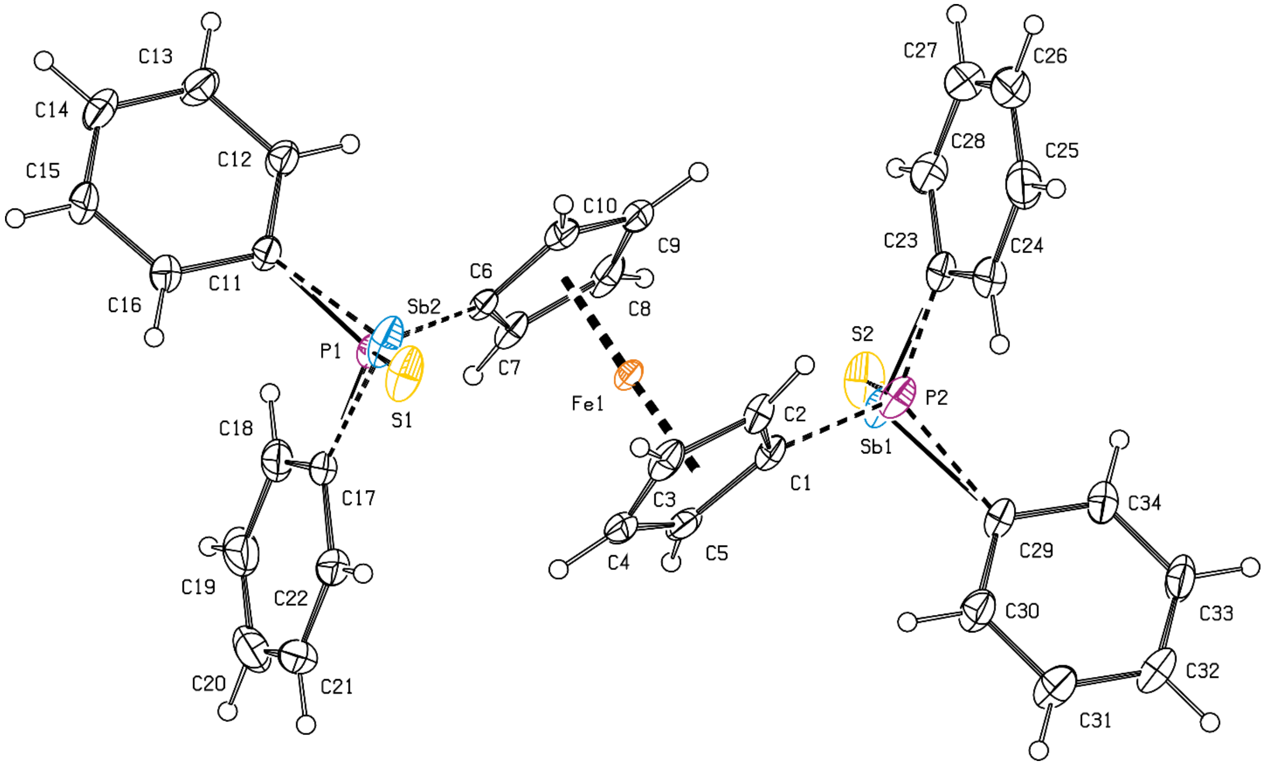

Figure S7. PLATON plot of the molecular structure of **1S** showing 30% probability ellipsoids and both contributing orientations of the P=S and Sb moieties.

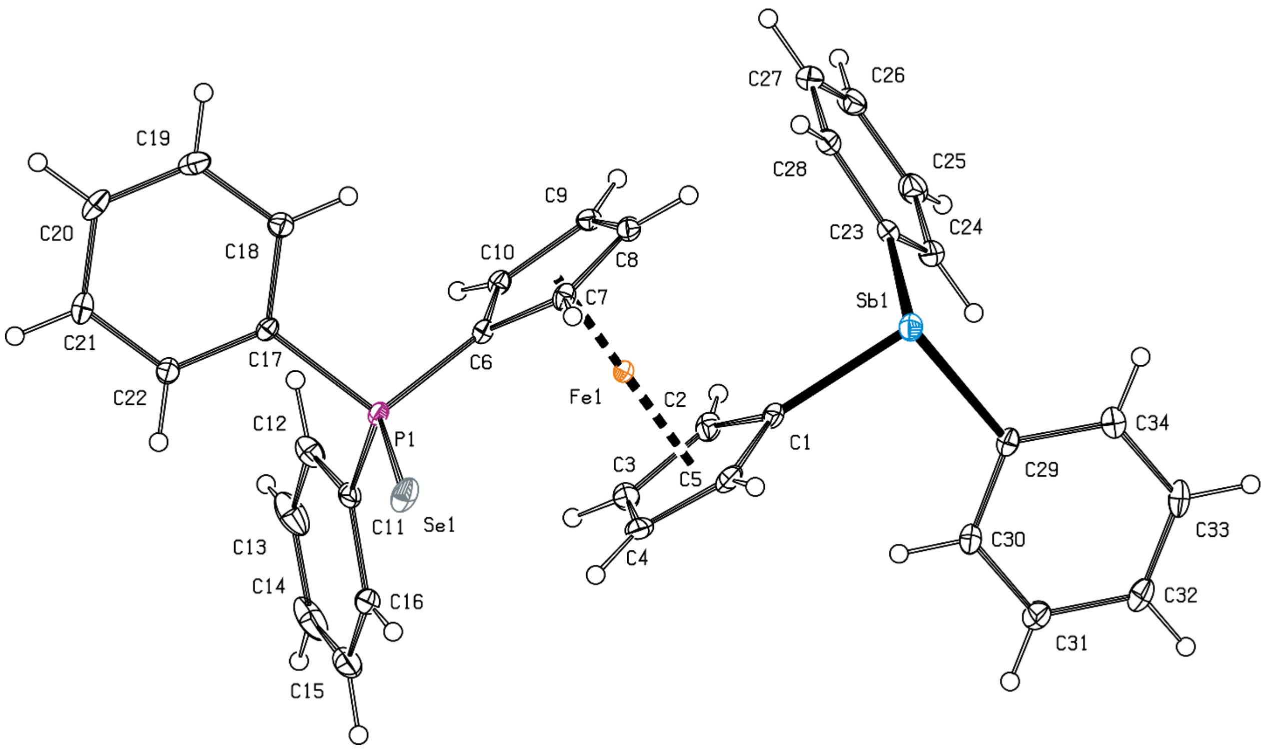

Figure S8. PLATON plot of the molecular structure of **1Se** showing 30% probability ellipsoids.

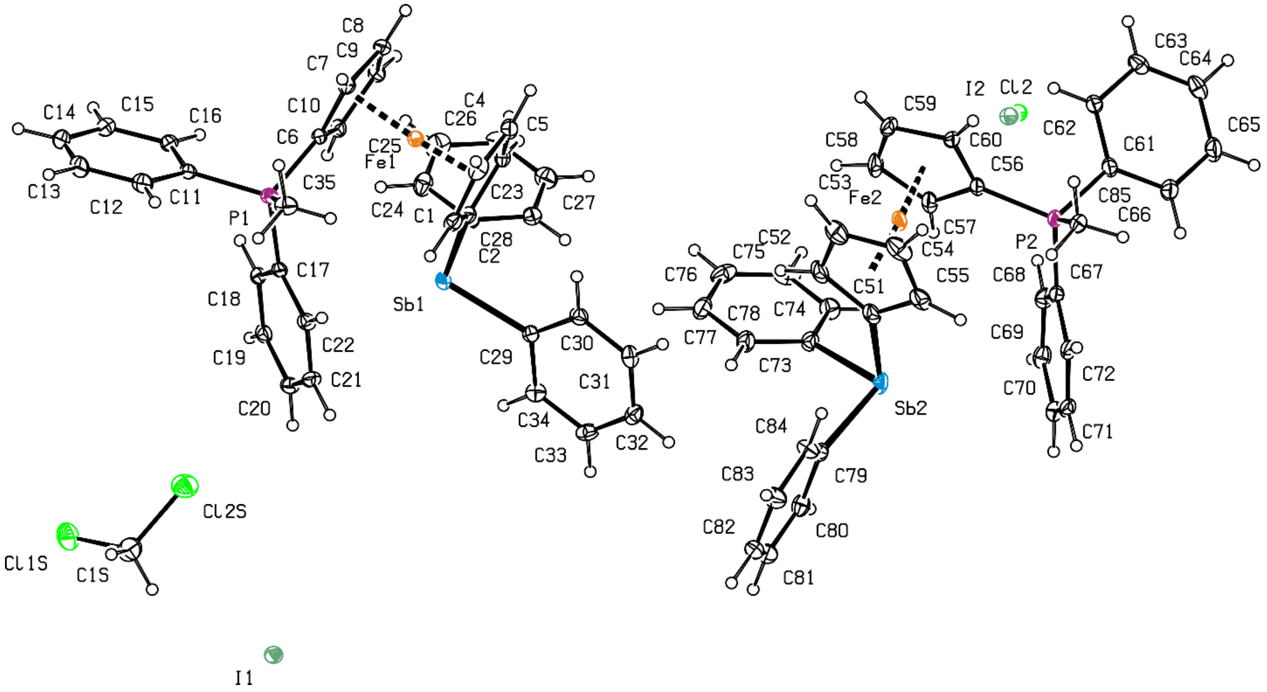

Figure S9. PLATON plot of the molecular structure of **1**·MeI·CH_2Cl_2 showing 30% probability ellipsoids.

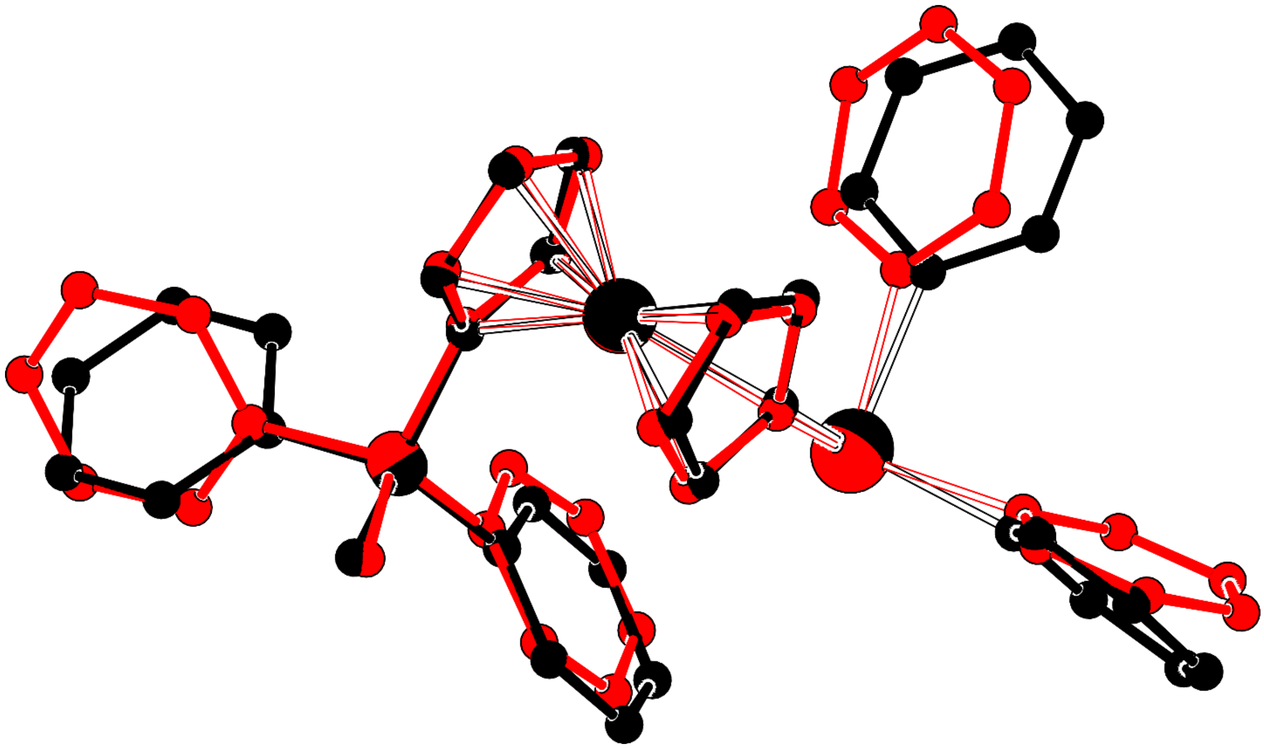

Figure S10. Least-squares overlap of the two structurally independent cations in the structure of **1**·MeI·CH_2Cl_2. Selected geometric data for molecule 1 [molecule 2] in Å and deg: Fe-C (range) 2.034(2)-2.060(2) [2.022(2)-2.057(3)], tilt 4.6(2) [4.3(2)], Sb-C(fc) 2.124(3) [2.135(3)], Sb-C(Ph) 2.156(3)/2.164(2) [2.148(3)/2.162(2)], P-C(fc) 1.765(3) [1.770(2)], P-C(Ph) 1.801(3)/1.801(3) [1.797(3)/1.788(3)], P-CH_3 1.779(3) [1.782(3)], C(fc)-Sb-C(Ph) 95.68(9)/93.80(9) [95.3(1)/94.3(1)], C(Ph)-Sb-C(Ph) 98.6(1) [98.3(1)], C(fc)-P-C(Ph) 109.1(1)/111.0(1) [109.5(1)/109.3(1)], C(Ph)-P-C(Ph) 107.8(1) [108.3(1)], CH_3-P-C(fc) 108.9(1) [110.0(1)], and CH_3-P-C(Ph) 109.4(1)/110.7(1) [107.9(1)/111.8(1)].

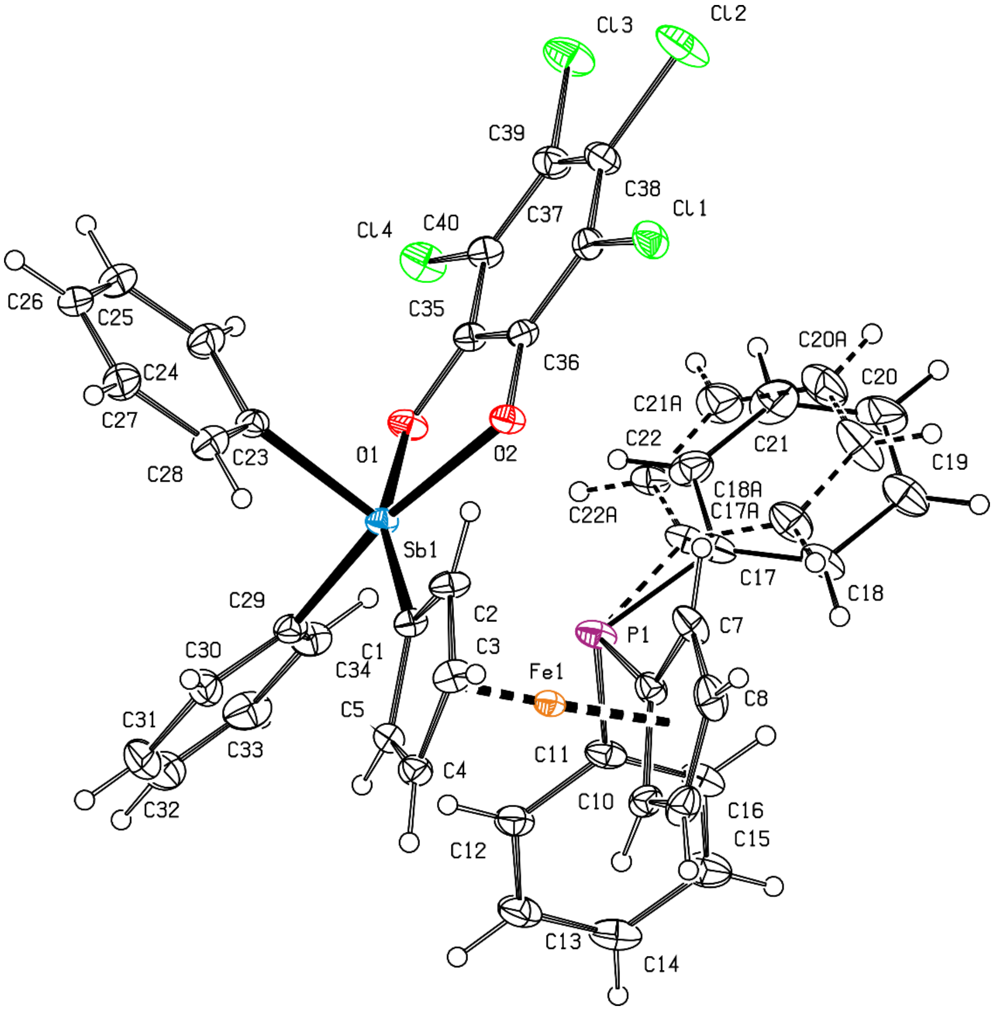

Figure S11. PLATON plot of the structure of **6**·C_6H_{14} showing 30% probability ellipsoids and both orientations of the disordered phenyl ring C(17-22). The solvent was removed by PLATON/SQUEEZE.

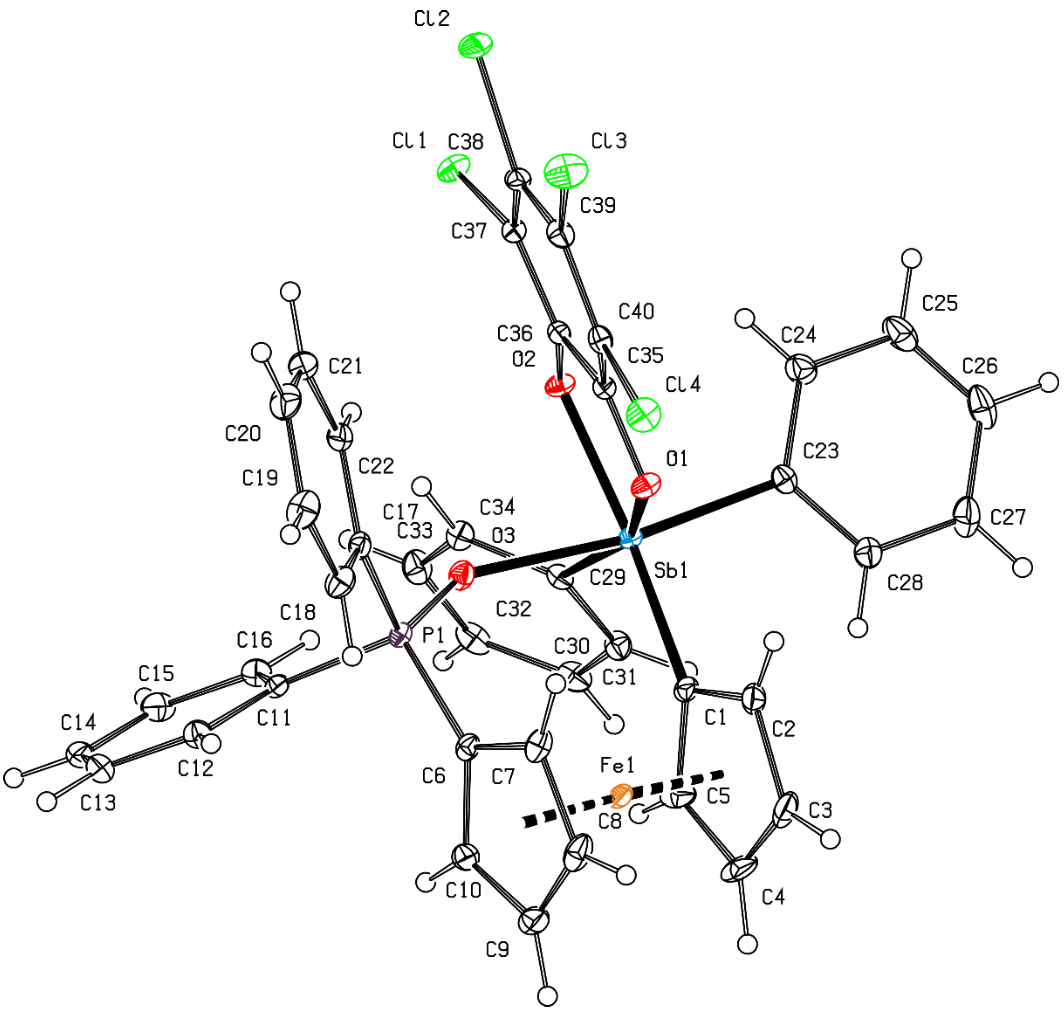

Figure S12. PLATON plot of the molecular structure of **6O**·$CHCl_3$ showing 30% probability ellipsoids. The solvent was omitted for clarity.

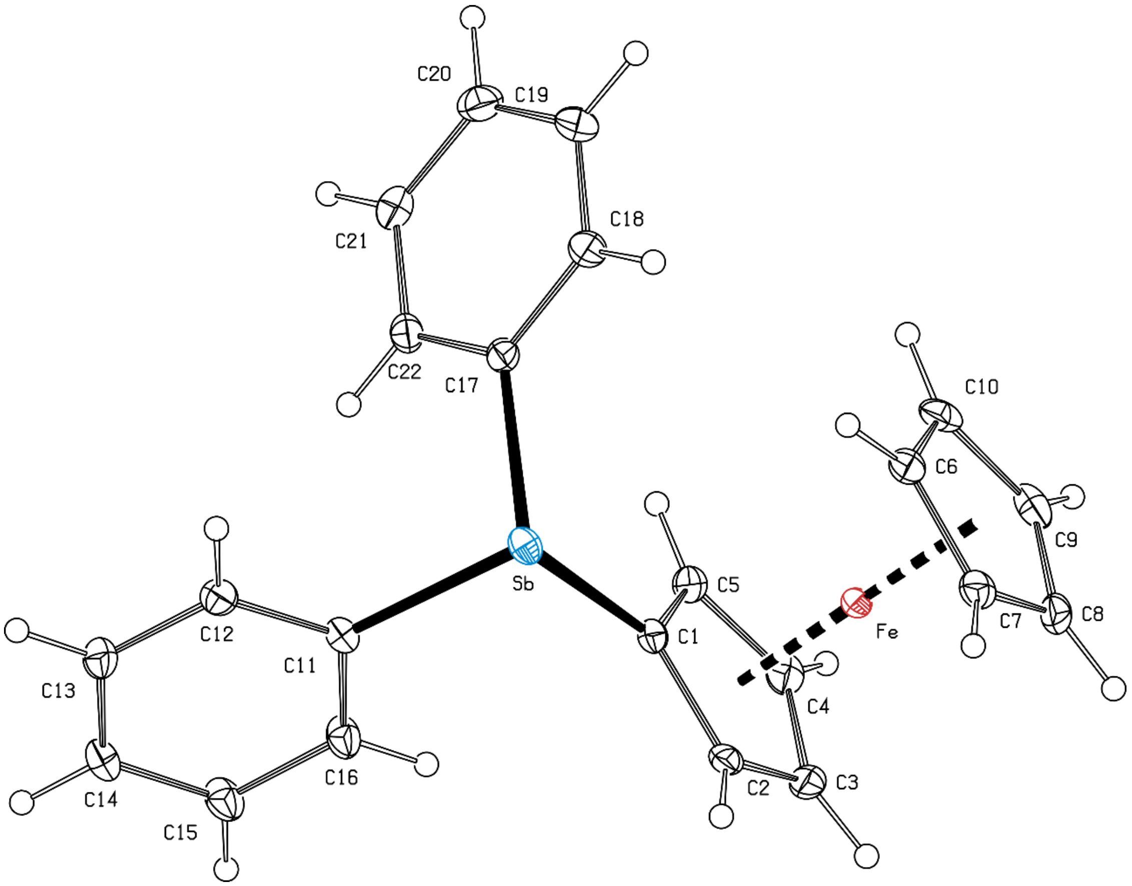

Figure S13. PLATON plot of the molecular structure of **4** showing 30% probability ellipsoids. Selected distances and angles (in Å and deg): Fe-C(1-10) range 2.039(2)-2.051(2), tilt 1.6(1), Sb-C1 2.119(2), Sb-C11 2.150(2), Sb-C17 2.157(2), C1-Sb-C11 95.53(6), C1-Sb-C17 96.38(7), C11-Sb-C17 96.57(7).

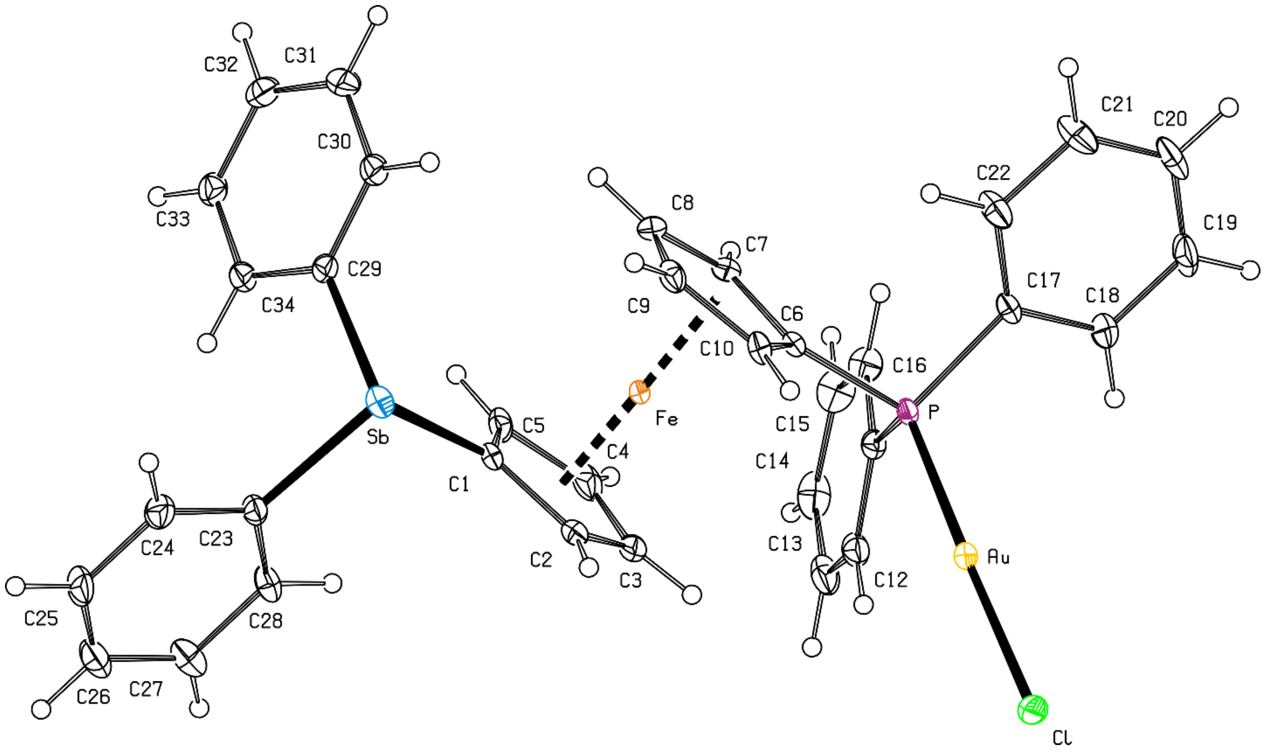

Figure S14. PLATON plot of the molecular structure of **7** showing 30% probability ellipsoids.

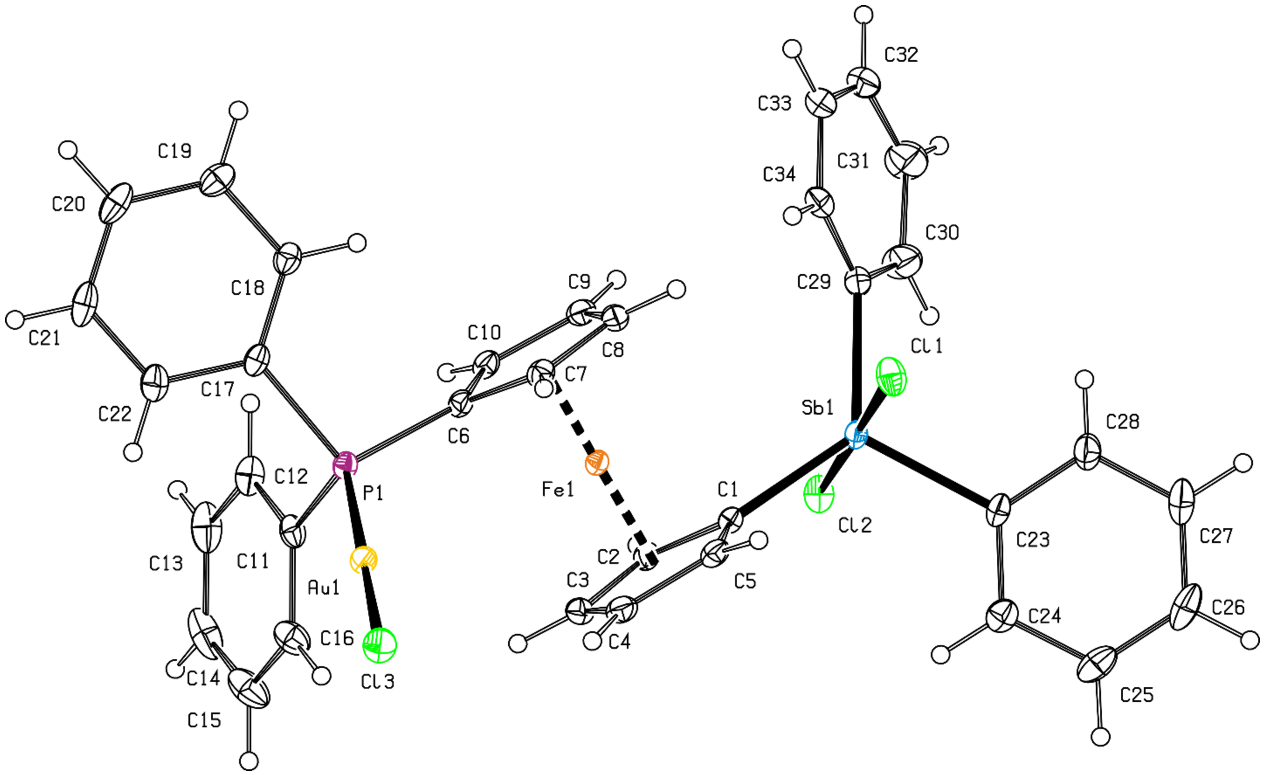

Figure S15. PLATON plot of the complex molecule in the structure of **12**·0.4$CHCl_3$ showing 30% probability ellipsoids.

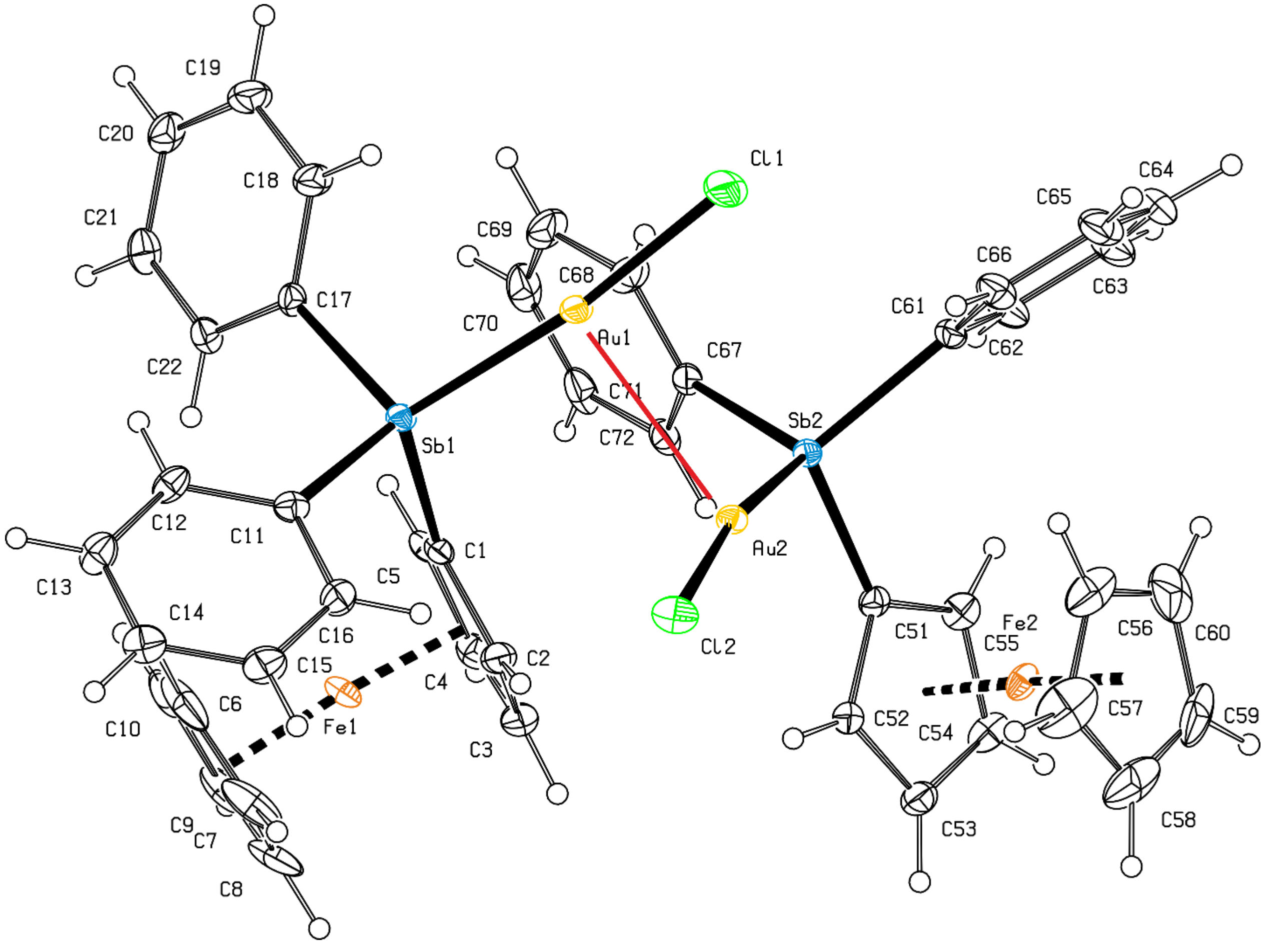

Figure S16. PLATON plot of the molecular structure of **9** showing 30% probability ellipsoids. The Au···Au contact between the crystallographically independent molecules is indicated by a red line.

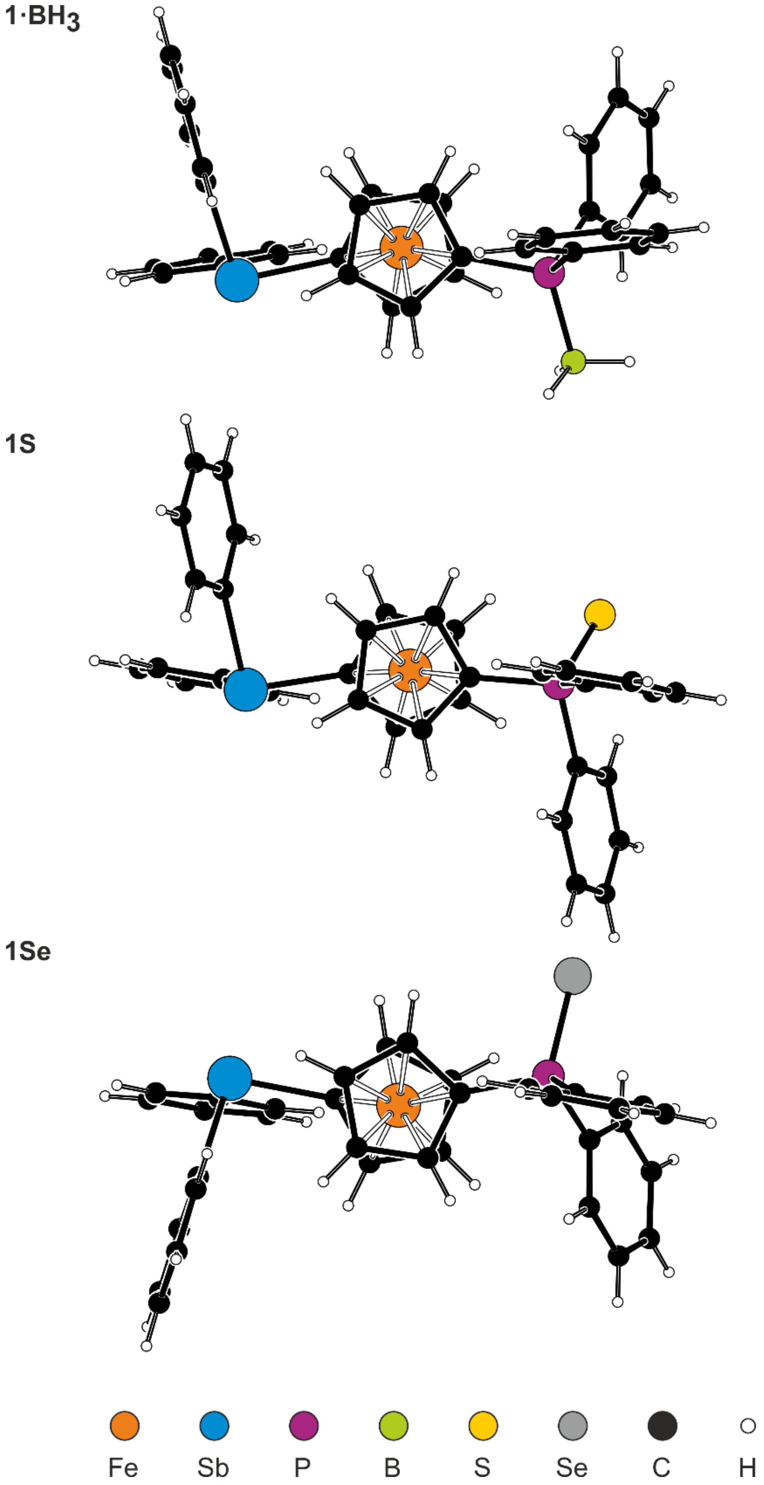

Figure S17. Views of the molecules of **1**·BH_3, **1S**, and **1Se** along the ferrocene axis.

Additional cyclic voltammograms

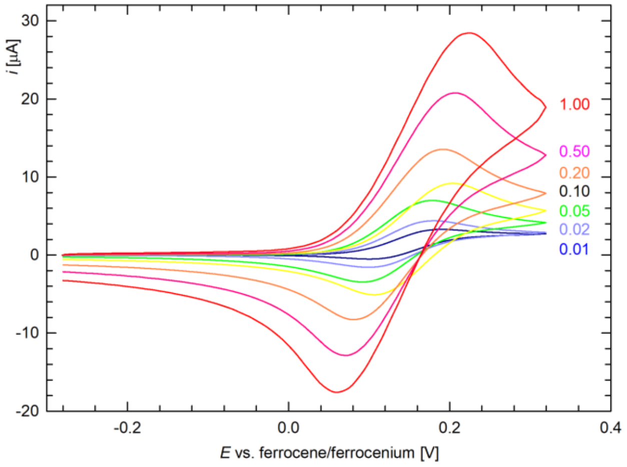

Figure S18. Cyclic voltammograms of **1** recorded at different scan rates (values in V s^{-1} are given in the Figure) at Pt disc electrode in dichloromethane containing 0.1 M $Bu_4N[PF_6]$.

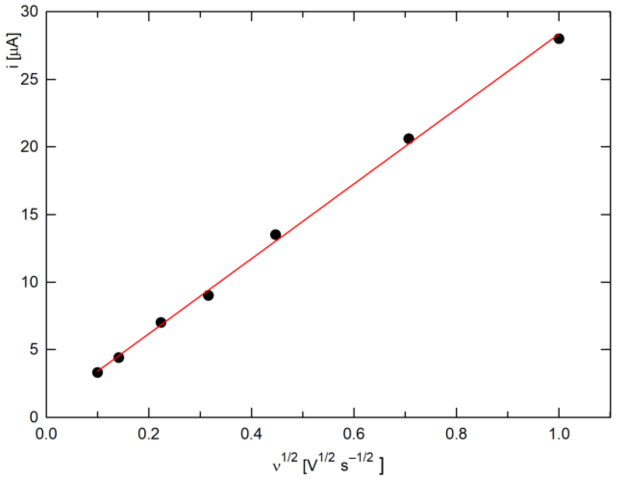

Figure S19. Dependence of the anodic peak current (i_{pa}) on the square-root of the scan rate (ν) for the primary electrochemical oxidation of compound **1**.

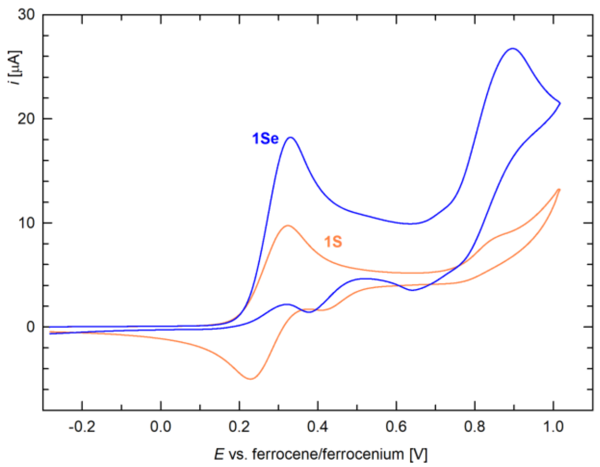

Figure S20. Cyclic voltammograms of **1S** and **1Se** (Pt disc electrode, CH_2Cl_2, 0.1 M $Bu_4N[PF_6]$, scan rate 0.1 V s^{-1}).

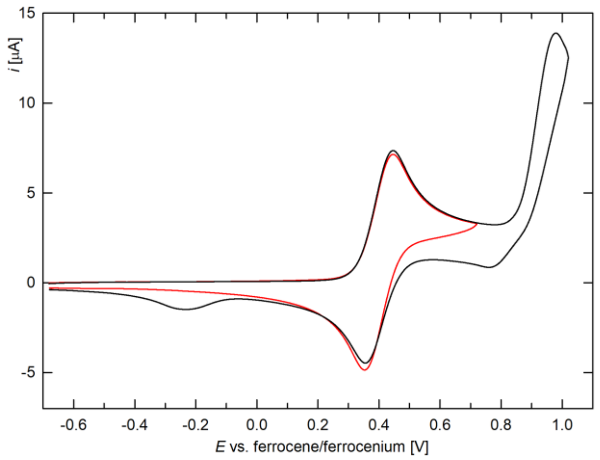

Figure S21. Cyclic voltammograms of **6O** (Pt disc electrode, CH_2Cl_2, 0.1 M $Bu_4N[PF_6]$, scan rate 0.1 V s^{-1}).

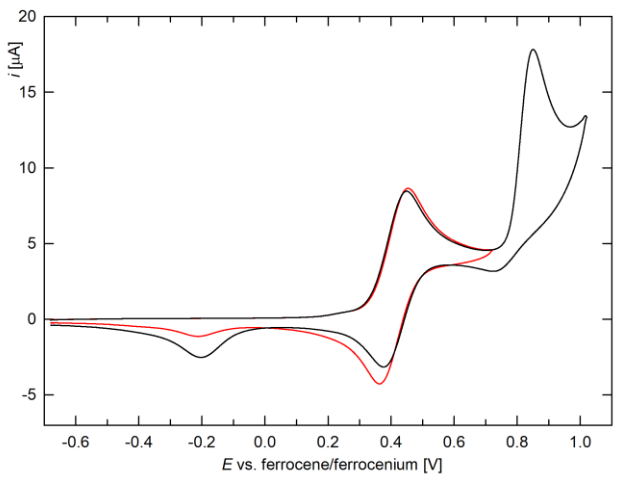

Figure S22. Cyclic voltammograms of **6S** (Pt disc electrode, CH_2Cl_2, 0.1 M $Bu_4N[PF_6]$, scan rate 0.1 V s^{-1}).

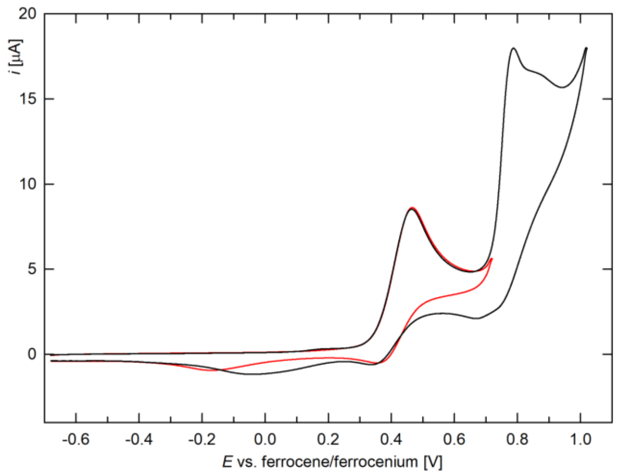

Figure S23. Cyclic voltammograms of **6Se** (Pt disc electrode, CH_2Cl_2, 0.1 M $Bu_4N[PF_6]$, scan rate 0.1 V s^{-1}).

DFT calculations

Theoretical calculations were performed using the Gaussian 16 program package.[16] The geometry optimizations were started from atomic coordinates determined by X-ray diffraction analysis if available and were performed using PBE0[17] density functional in conjunction with the Stuttgart effective core potential[18] for the metal atoms (Fe, Sb) and the def2-TZVP[19] basis set for the remaining elements (C, H, O, P, S, Cl, Se) with added Grimme's D3 dispersion correction.[20] Solvent effects (chloroform) were approximated using the PCM model.[21] The methyl cation (MCA) and fluoride ion affinities (FIA) were calculated according to literature procedures.[22,23] The fluoride ion affinities were calculated using the geometries and the thermal corrections obtained at the PBE0(d3)/def2-TZVP:sdd(Fe,Sb) level of theory combined with the PW6B95(d3bj)/def2-qzvpp single-point electronic energies.[24] Orbital composition analysis based on the Natural Atomic Orbitals (NAO)[25] (at the PBE0(d3)/def2-TZVP level of theory), as well as the analysis of calculated electron densities by the Atoms in Molecules approach (AIM), were performed using the Multiwfn software package (version 3.8).[26] Molecular orbitals were visualized using the Avogadro programme.[27] Intrinsic bond orbital (IBO) analysis and visualization of the obtained orbitals were performed using the IboView software.[28]

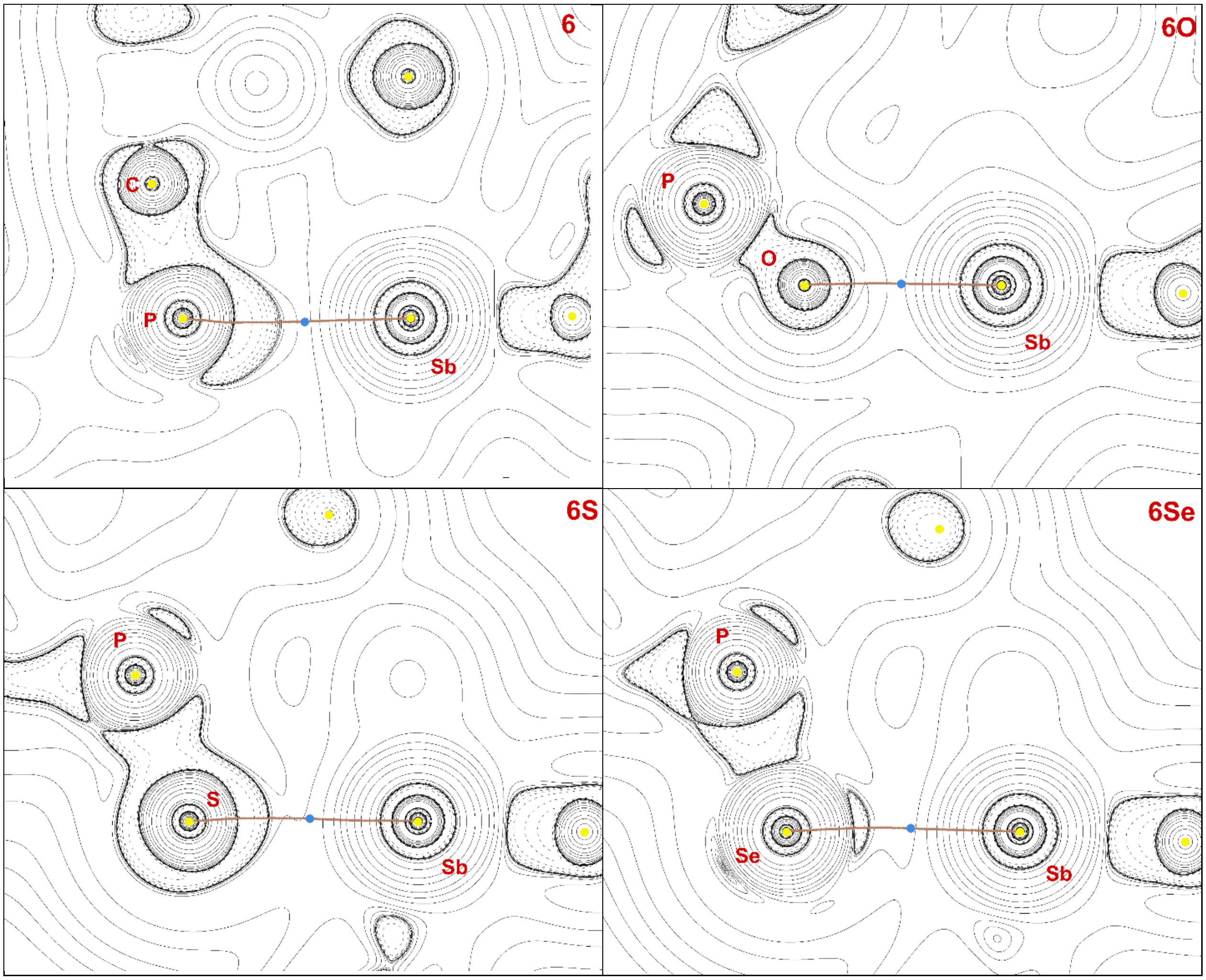

Figure S24. Laplacian contours (positive – full black lines; negative – dashed black lines], bond paths (brown lines), atomic critical points (yellow circles), and bond critical points (blues circles) in the plane defined by the P, Sb and C^{ipso} of C_5H_4P atoms for **6**, and by the P, Sb and chalcogenide atoms (E = O, S, and Se) for **6O**, **6S**, and **6Se**.

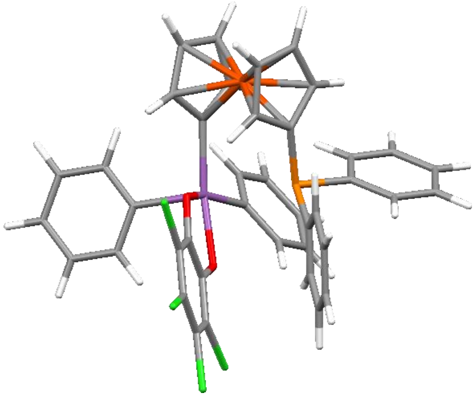

6 (closed)

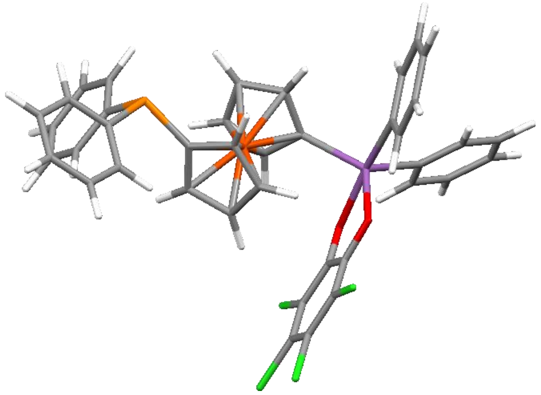

6 (open)

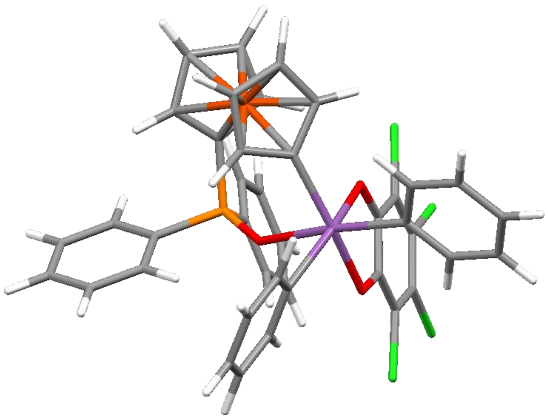

6O (closed)

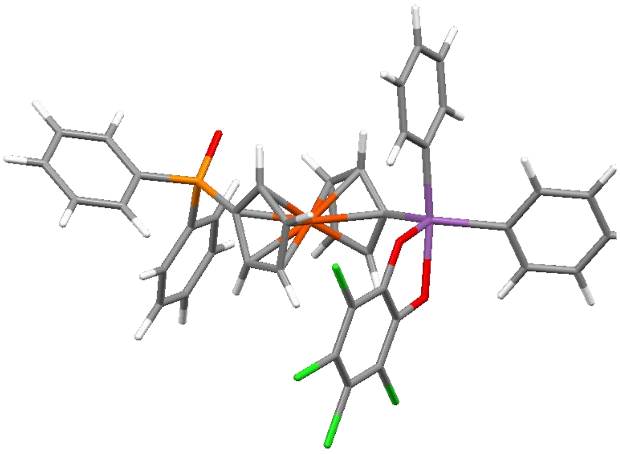

6O (open)

Figure S25. Diagrams of the DFT-optimized closed (left) and open (right) forms of **6** and **6O**.

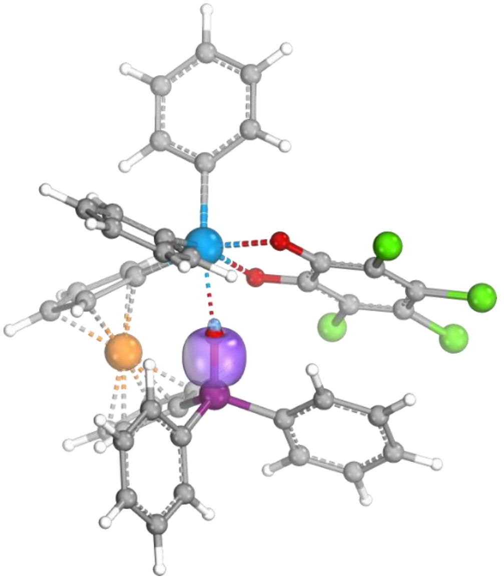

σ (P–O) [1.39(O)/0.59(P)]

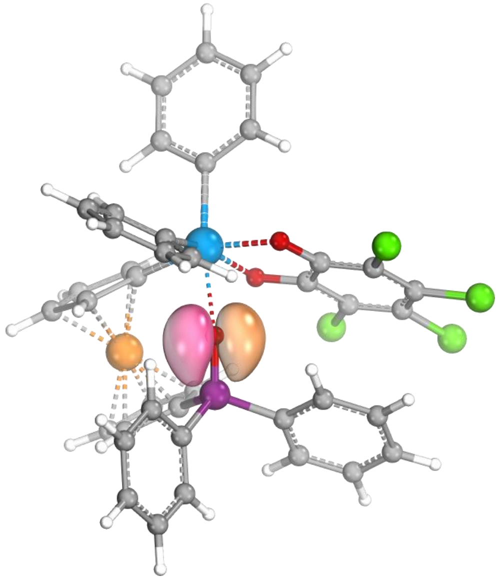

lp(O) → π*(P–C) [1.85(O)/0.09(P)]

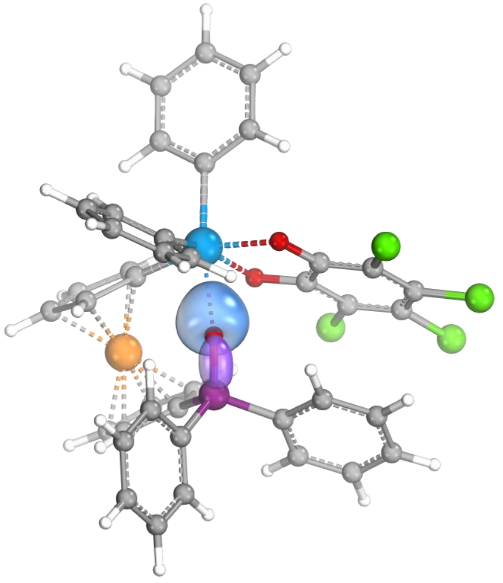

lp(O) → π*(P–C) [1.76(O)/0.15(Sb)/0.04(P)]

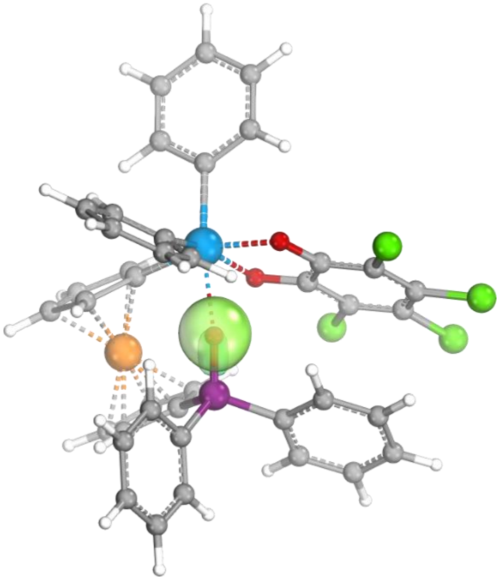

lp(O) → π*(P–C) [1.90(O)/0.05(P)]

Figure S26. Selected intrinsic bond orbitals (IBOs) of stiborane **6O**. Values in parentheses indicate the fraction of bonding electrons assigned to the individual atoms (lp = lone electron pair).

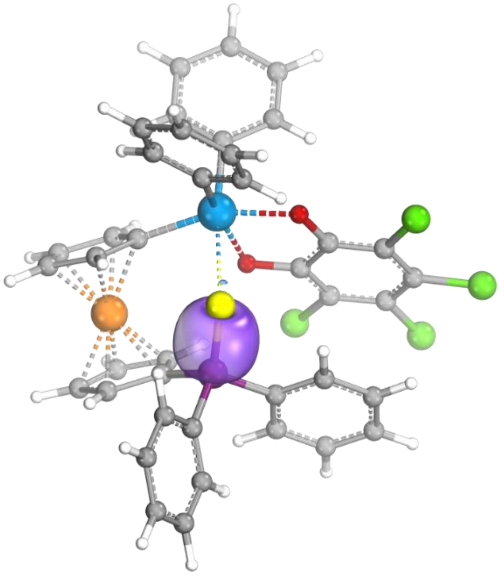

σ (P–S) [1.01(S)/0.98(P)]

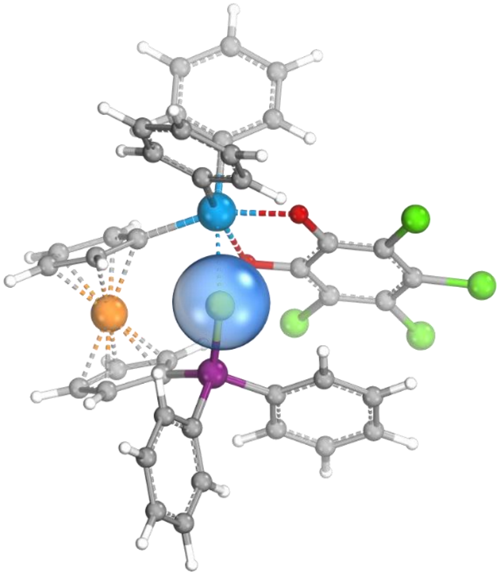

lp(S) [1.97(S)]

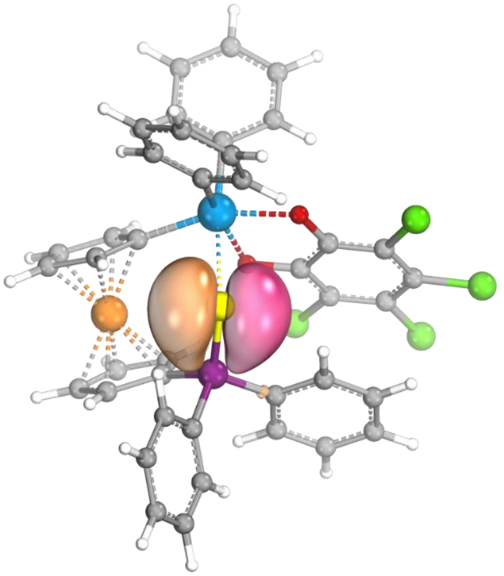

lp(S) → π*(P–C) [1.84(S)/0.08(P)]

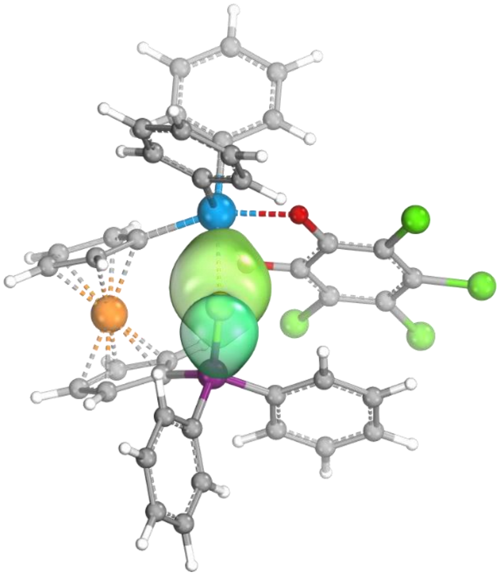

lp(S) → π*(P–C) [1.70(S)/0.17(Sb)0.05(P)]

Figure S27. Selected intrinsic bond orbitals (IBOs) of stiborane **6S**. Values in parentheses indicate the fraction of bonding electrons assigned to the individual atoms (lp = lone electron pair).

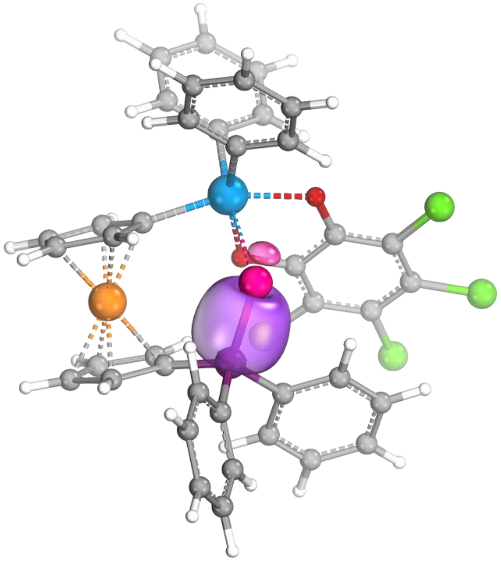

σ (P–Se) [1.01(Se)/0.98(P)]

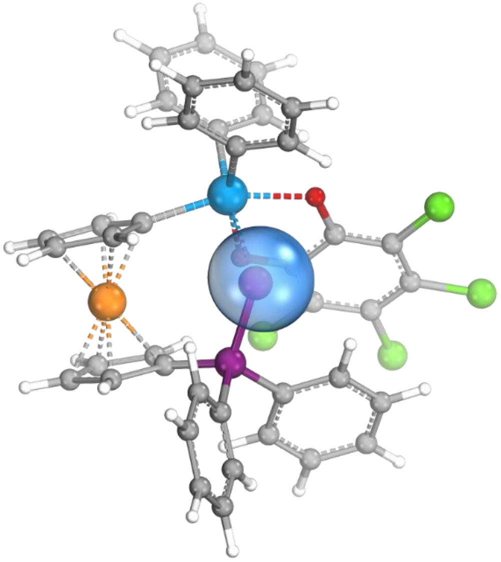

lp(Se) [1.97(Se)]

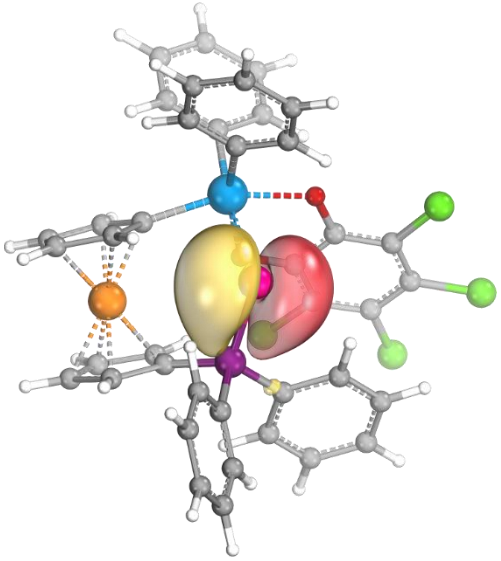

lp(Se) → π*(P–C) [1.84(Se)/0.08(P)]

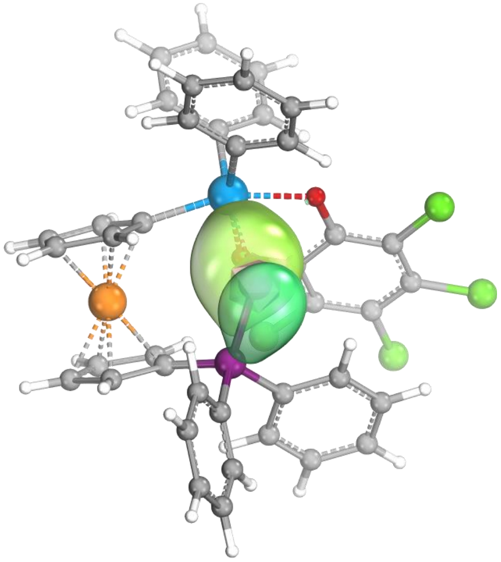

lp(Se) → π*(P–C) [1.70(Se)/0.17(Sb)0.05(P)]

Figure S28. Selected intrinsic bond orbitals (IBOs) of stiborane **6Se**. Values in parentheses indicate the fraction of bonding electrons assigned to the individual atoms (lp = lone electron pair).

Copies of the NMR spectra

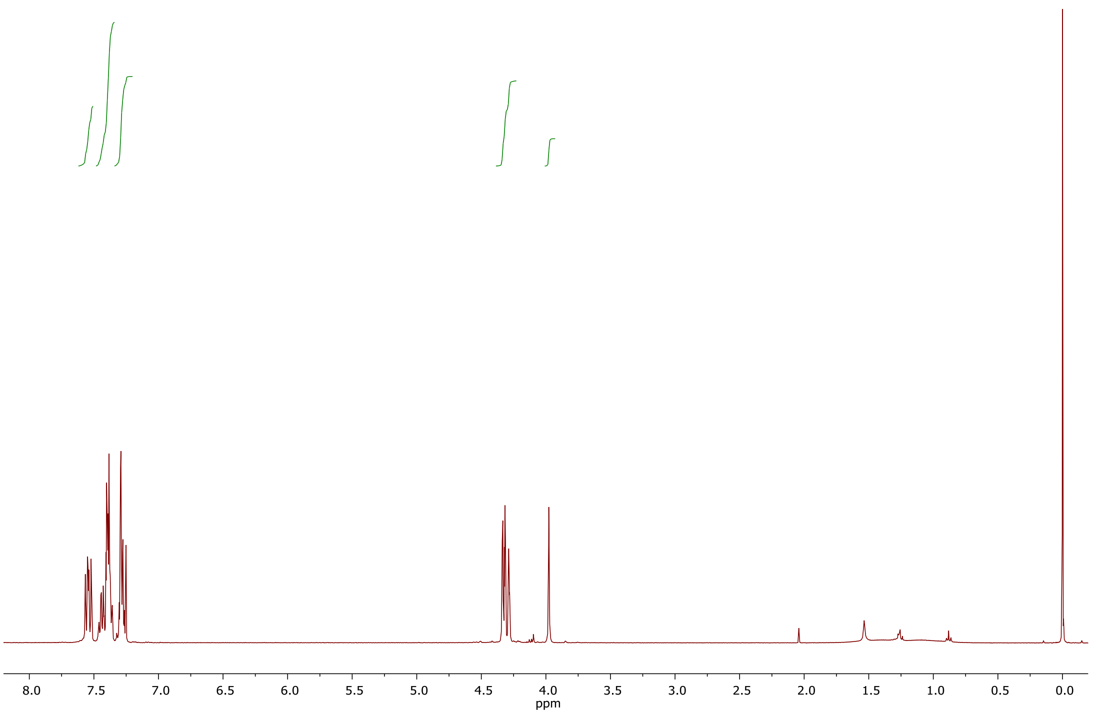

Figure S29. ^{1}H NMR spectrum (399.95 MHz, $CDCl_3$) of **1**·BH_3.

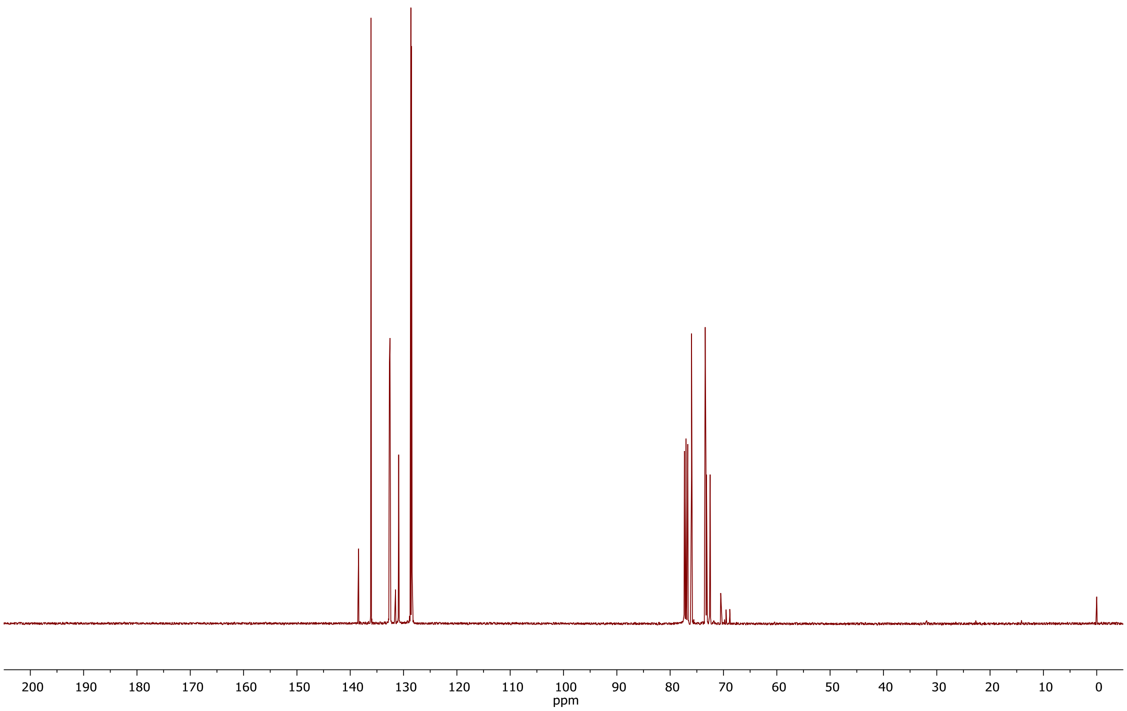

Figure S30. $^{13}C\{^{1}H\}$ NMR spectrum (100.58 MHz, $CDCl_3$) of **1**·BH_3.

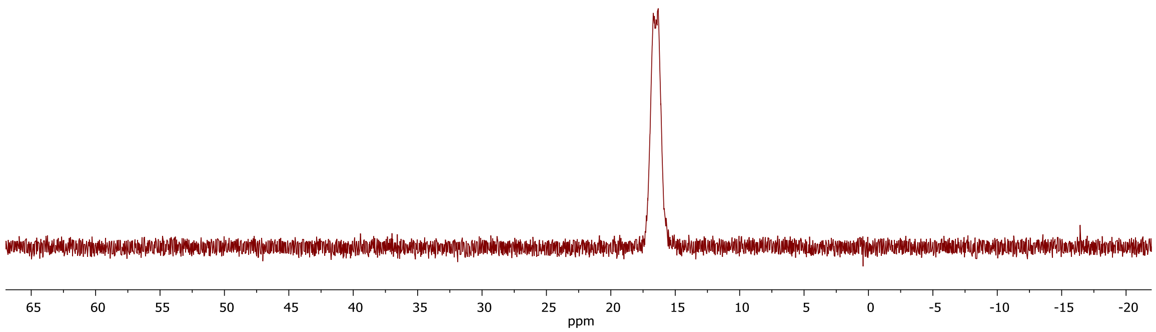

Figure S31. $^{31}P\{^{1}H\}$ NMR spectrum (161.90 MHz, $CDCl_3$) of **1**·BH_3.

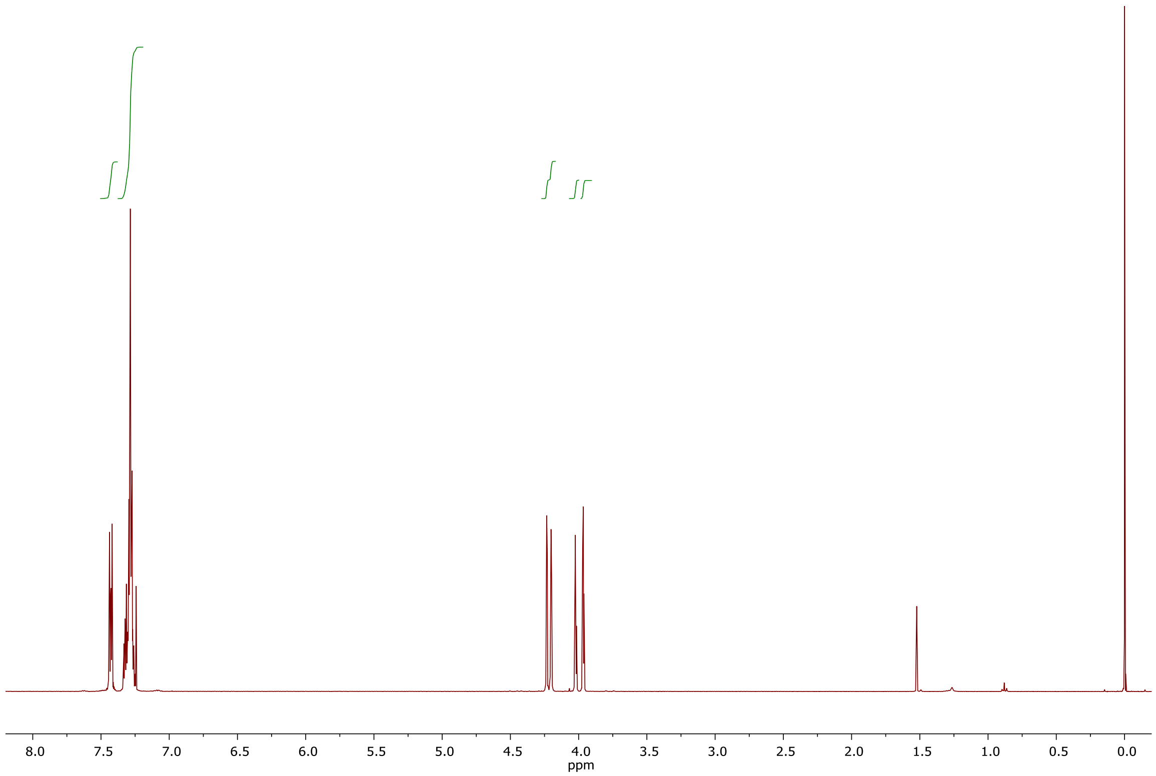

Figure S32. ^{1}H NMR spectrum (399.95 MHz, CD_2Cl_2) of **1**.

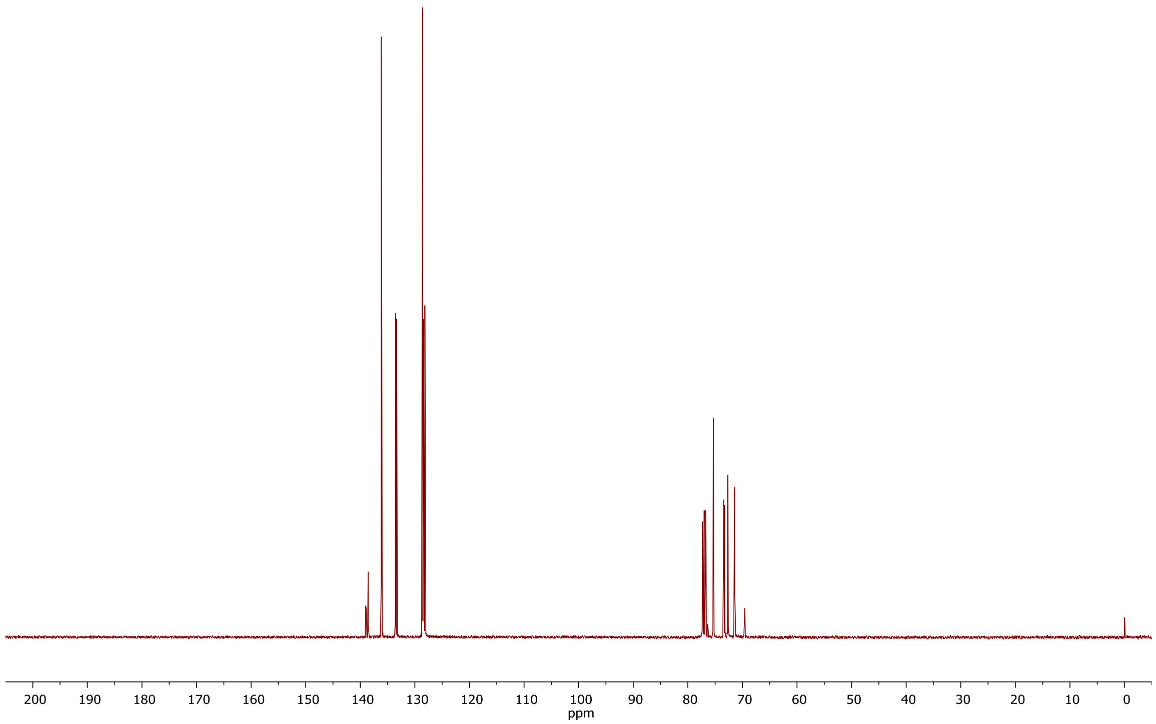

Figure S33. $^{13}C\{^{1}H\}$ NMR spectrum (100.58 MHz, $CDCl_3$) of **1**.

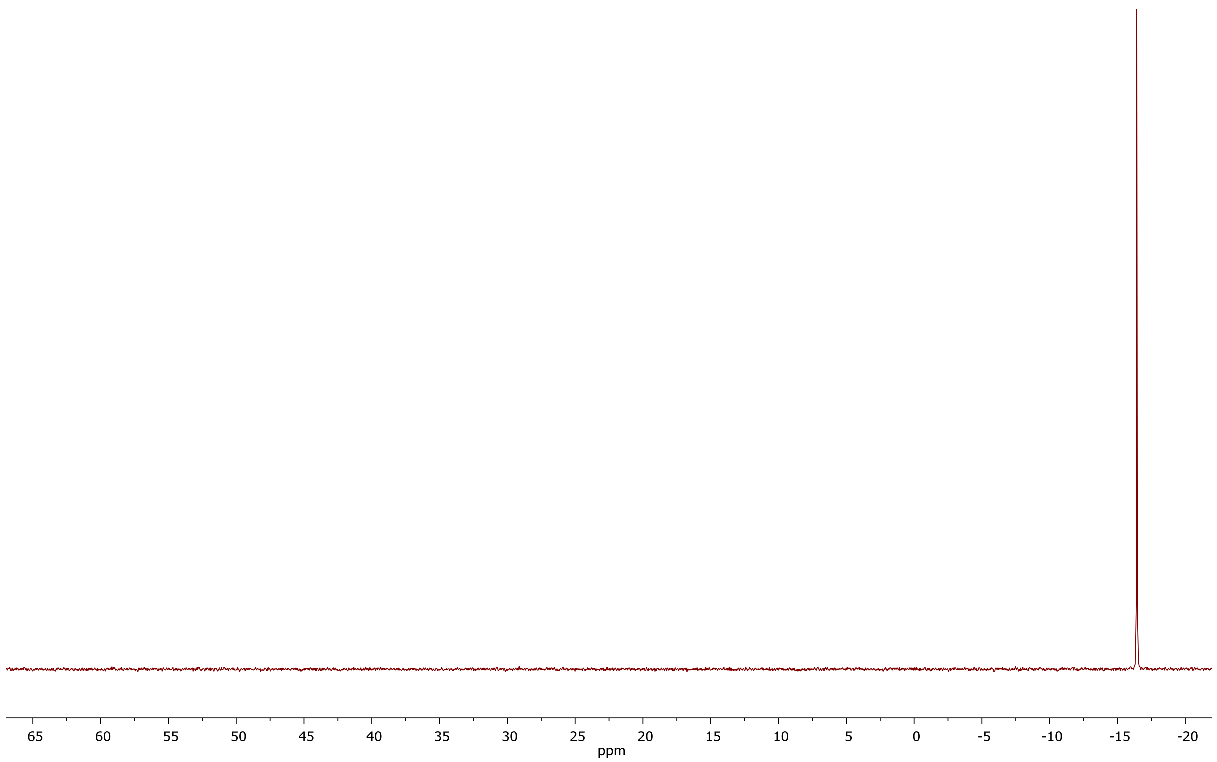

Figure S34. $^{31}P\{^1H\}$ NMR spectrum (161.97 MHz, CD_2Cl_2) of **1**.

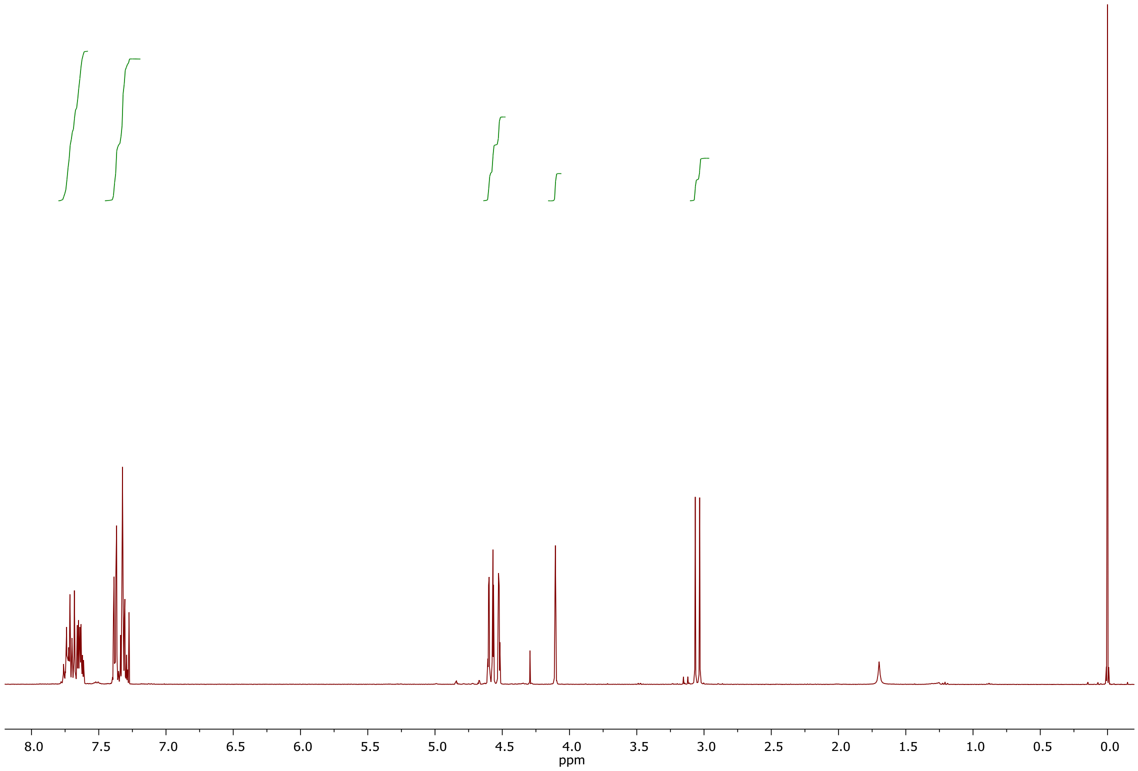

Figure S35. ^{1}H NMR spectrum (399.95 MHz, $CDCl_3$) of **1**·MeI.

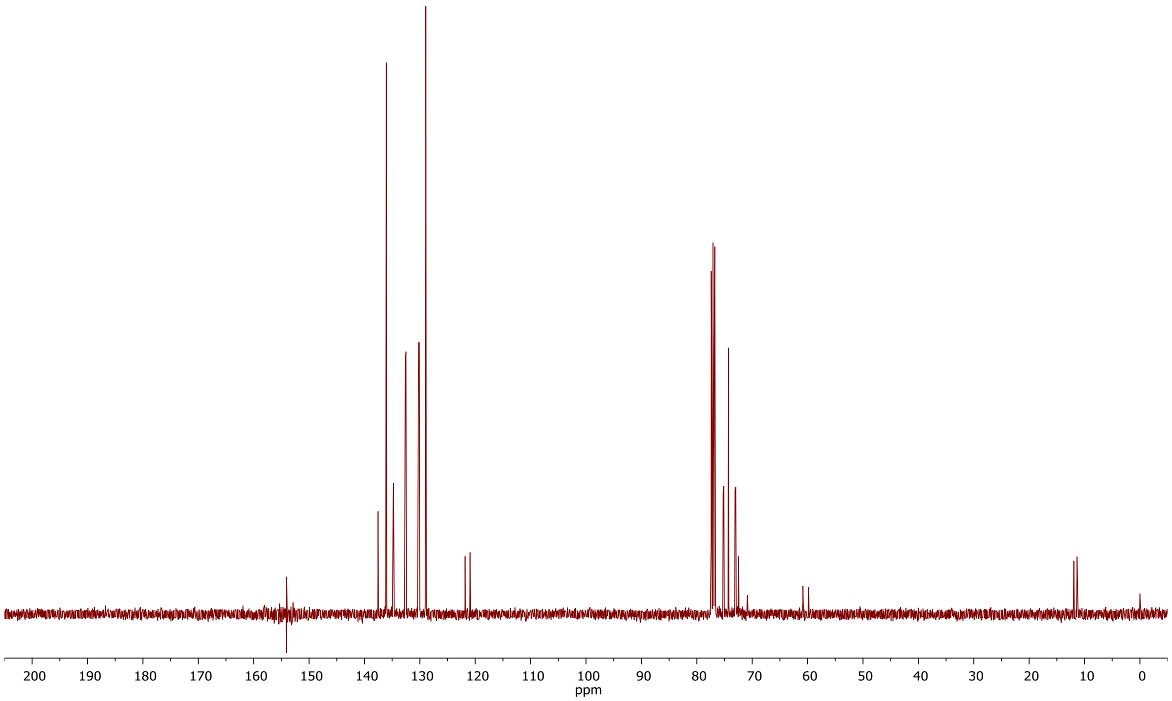

Figure S36. $^{13}C\{^{1}H\}$ NMR spectrum (100.58 MHz, $CDCl_3$) of **1**·MeI.

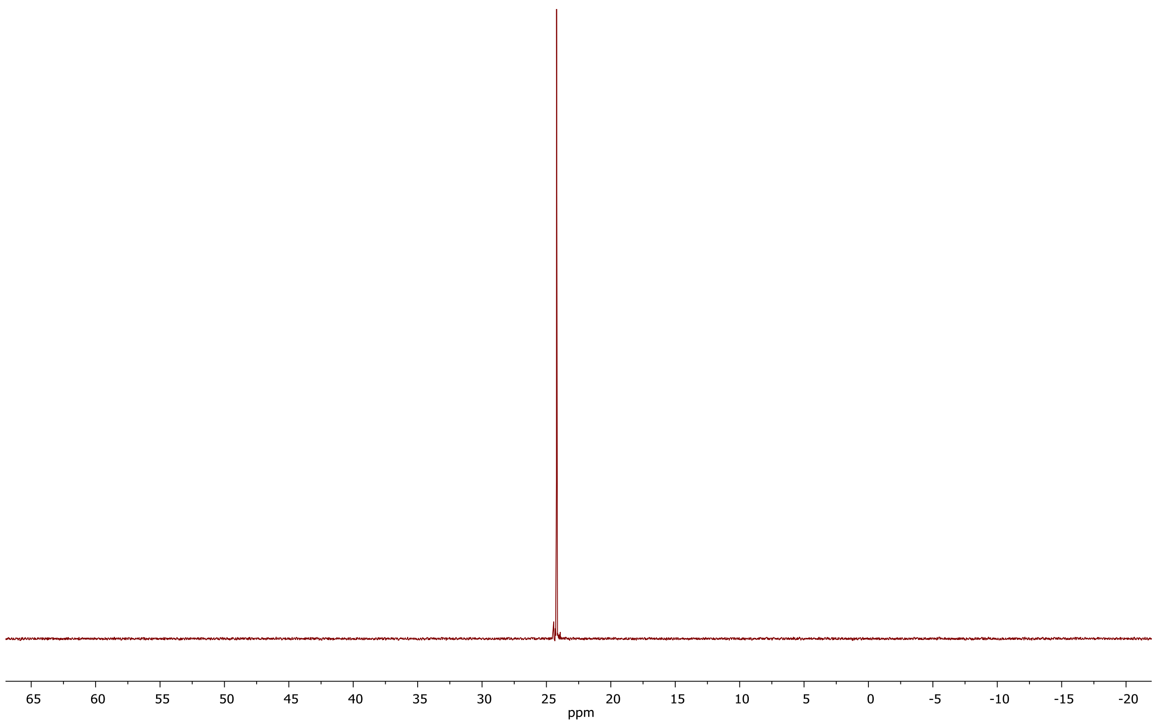

Figure S37. $^{31}P\{^{1}H\}$ NMR spectrum (161.90 MHz, $CDCl_3$) of **1**·MeI.

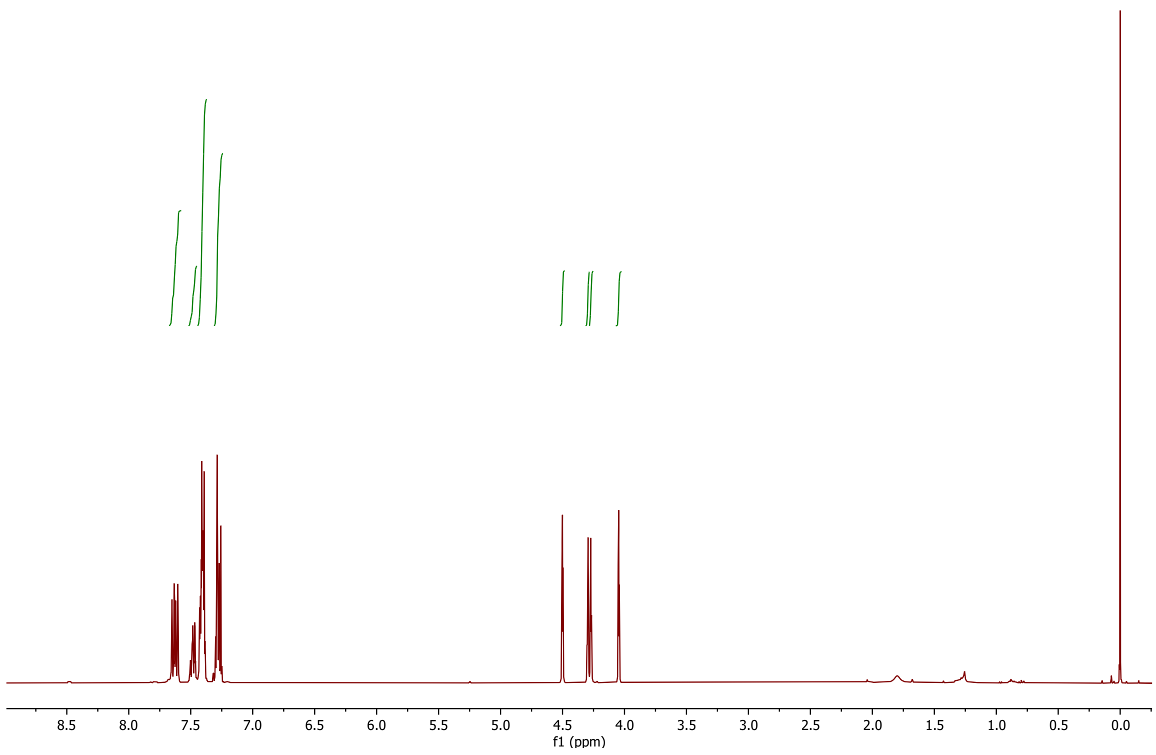

Figure S38. ^{1}H NMR spectrum (399.95 MHz, $CDCl_3$) of **1O**.

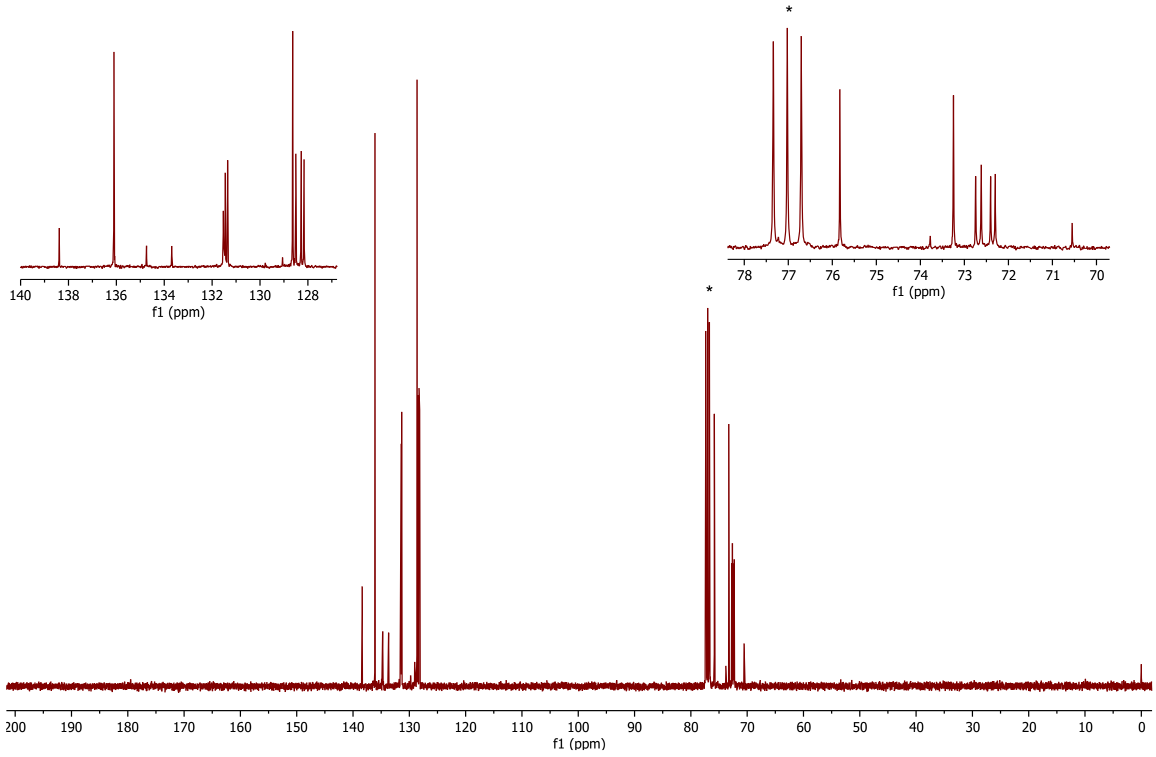

Figure S39. $^{13}C\{^{1}H\}$ NMR spectrum (100.58 MHz, $CDCl_3$) of **1O**.

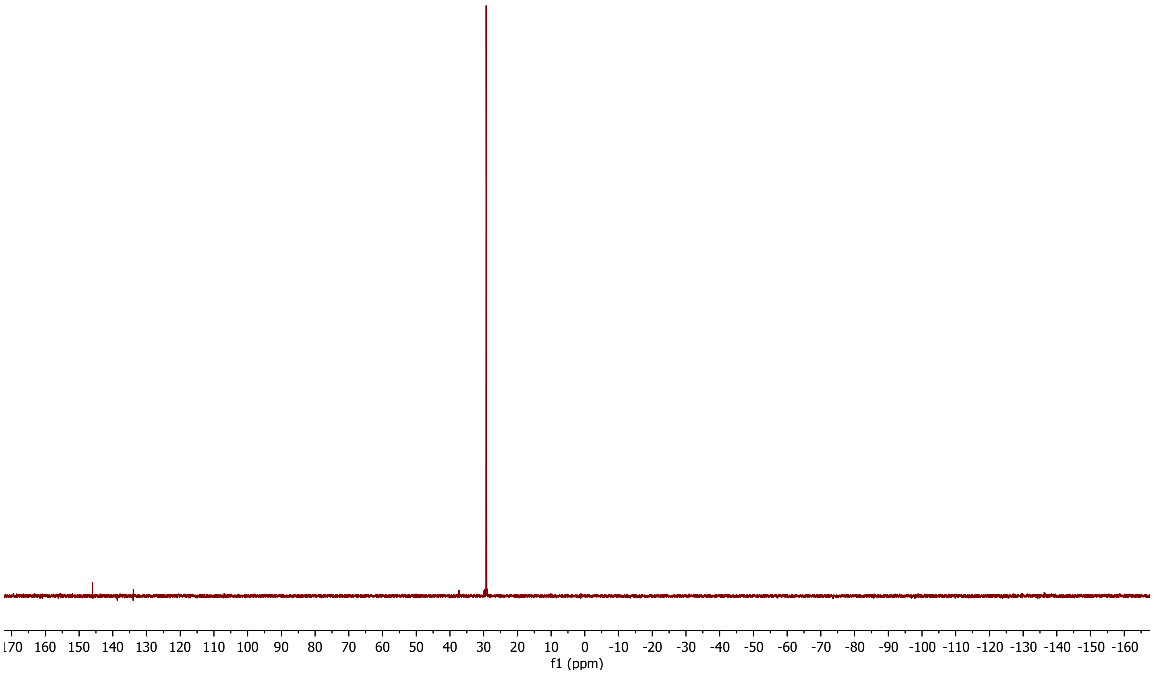

Figure S40. $^{31}P\{^{1}H\}$ NMR spectrum (161.90 MHz, $CDCl_3$) of **1O**.

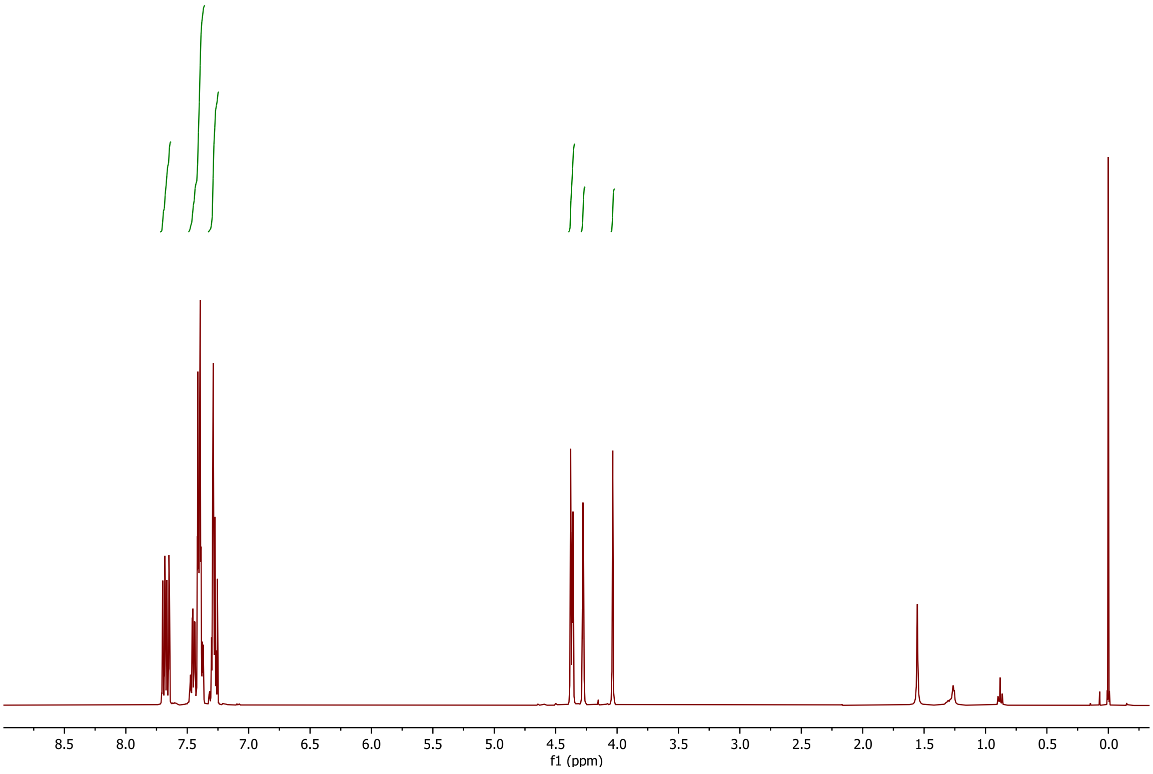

Figure S41. ^{1}H NMR spectrum (399.95 MHz, $CDCl_3$) of **1S**.

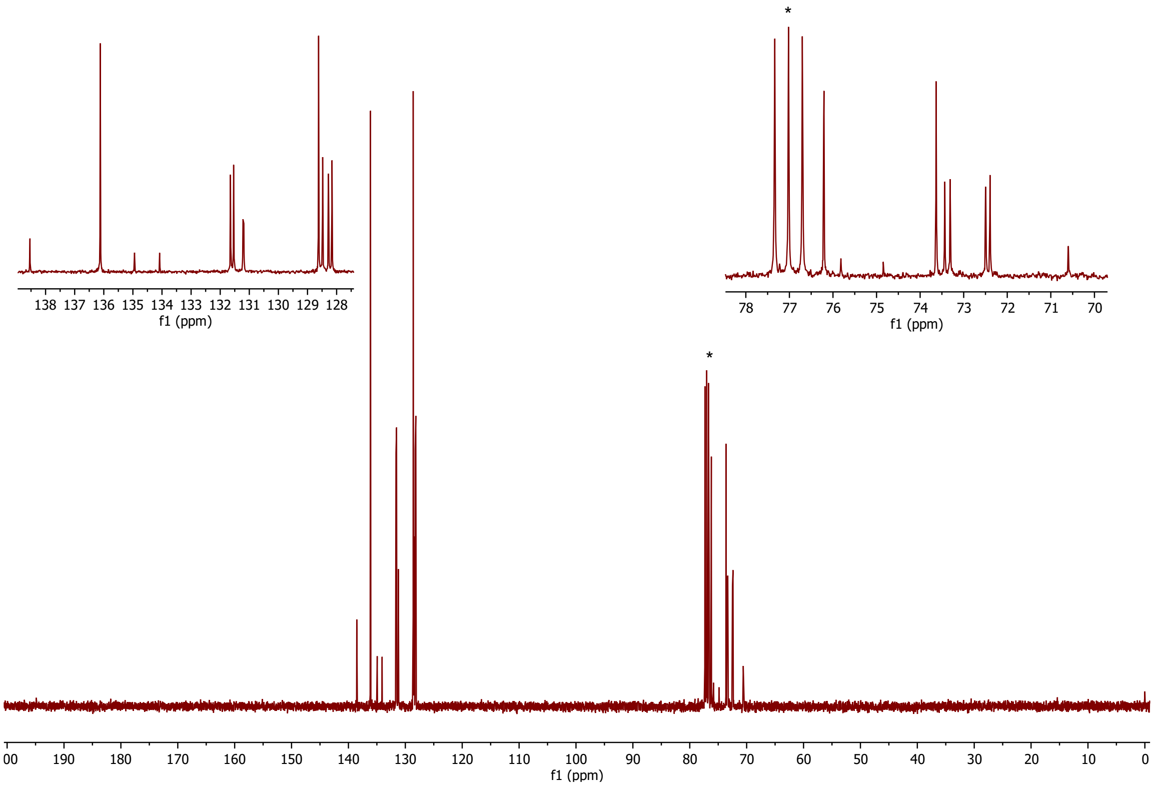

Figure S42. $^{13}C\{^{1}H\}$ NMR spectrum (100.58 MHz, $CDCl_3$) of **1S**.

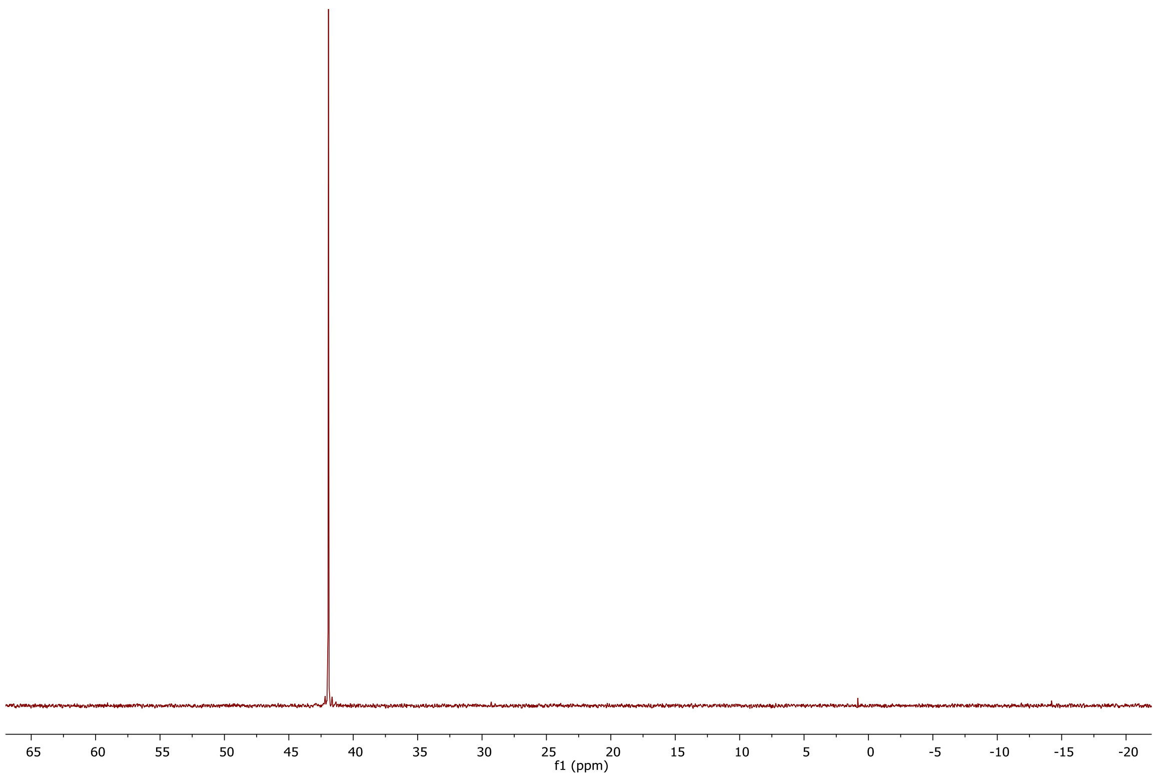

Figure S43. $^{31}P\{^1H\}$ NMR spectrum (161.90 MHz, $CDCl_3$) of **1S**.

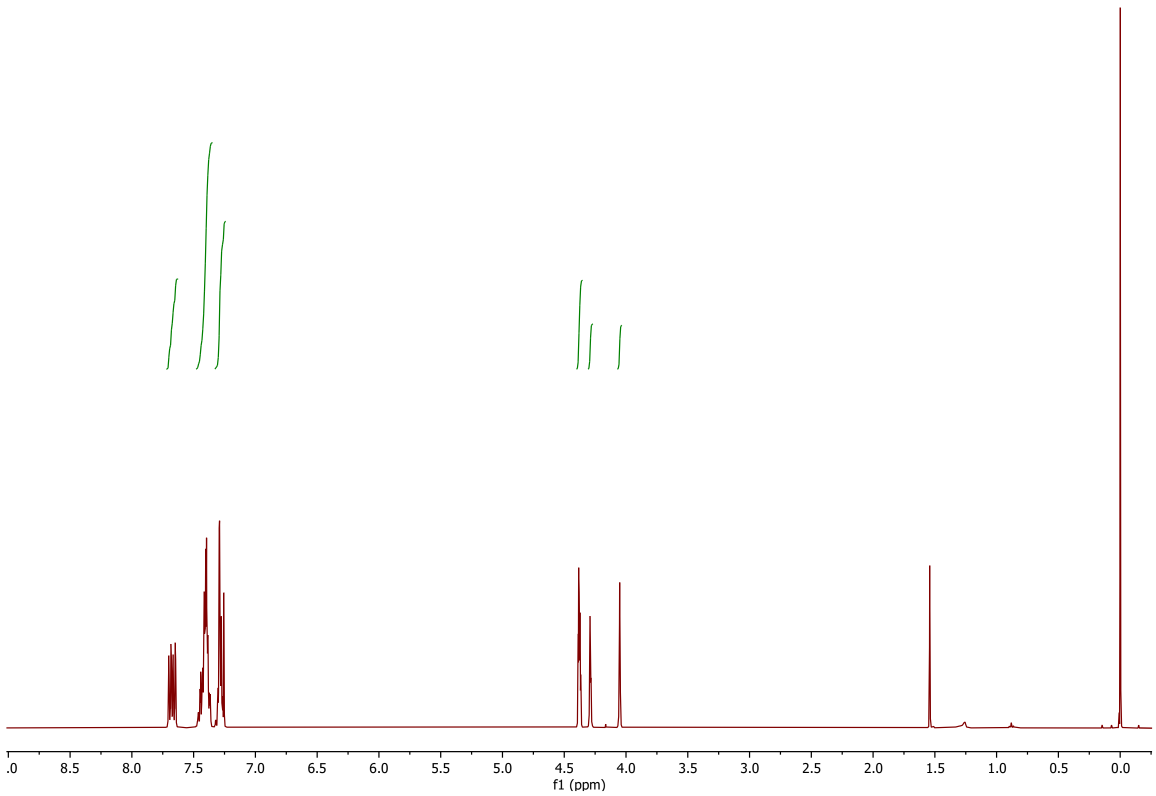

Figure S44. ^{1}H NMR spectrum (399.95 MHz, $CDCl_3$) of **1Se**.

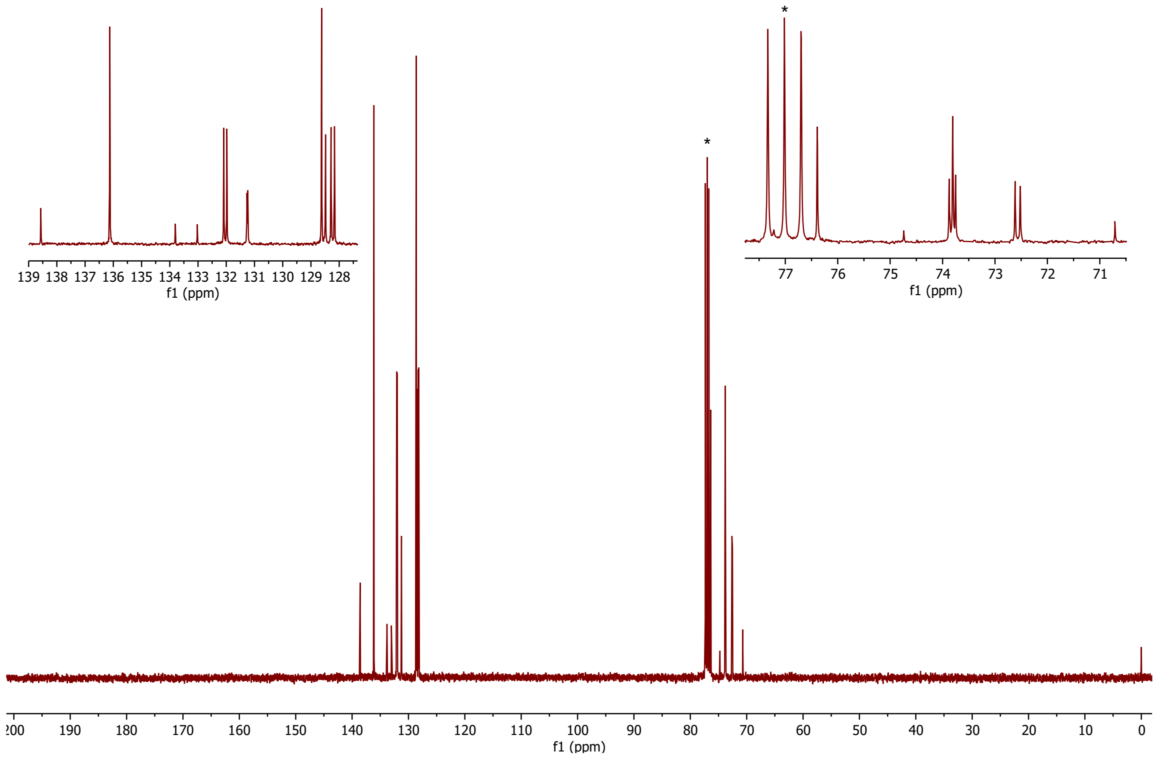

Figure S45. $^{13}C\{^{1}H\}$ NMR spectrum (100.58 MHz, $CDCl_3$) of **1Se**.

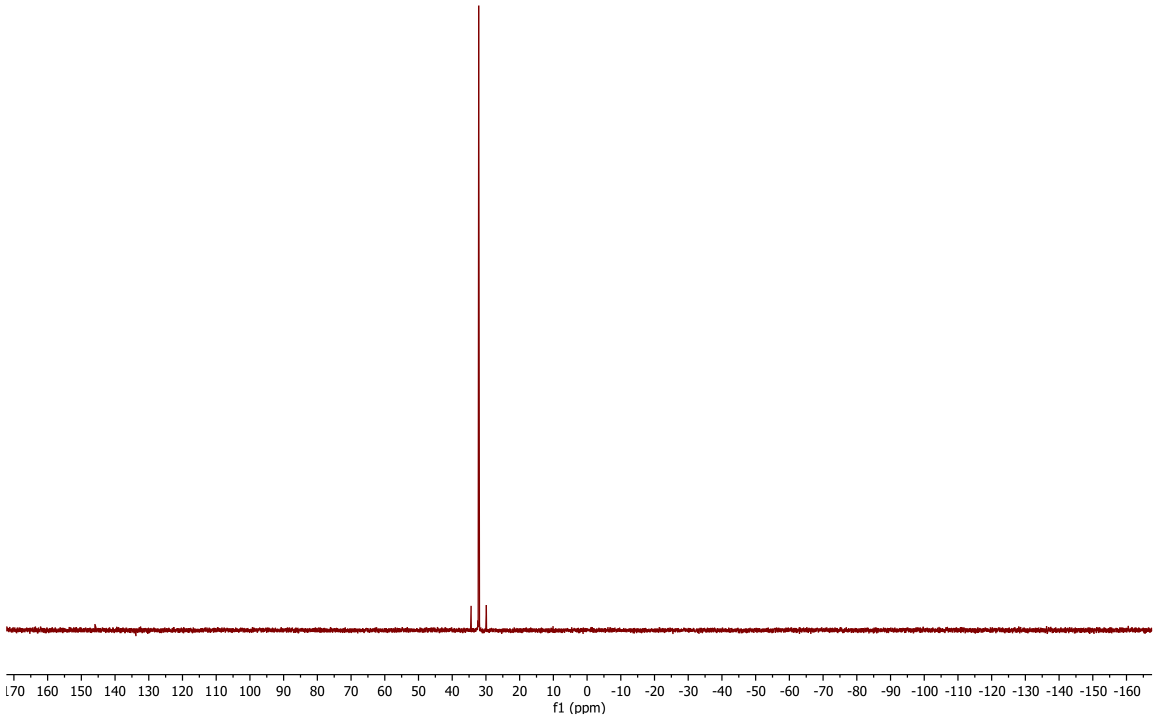

Figure S46. $^{31}P\{^{1}H\}$ NMR spectrum (161.90 MHz, $CDCl_3$) of **1Se**.

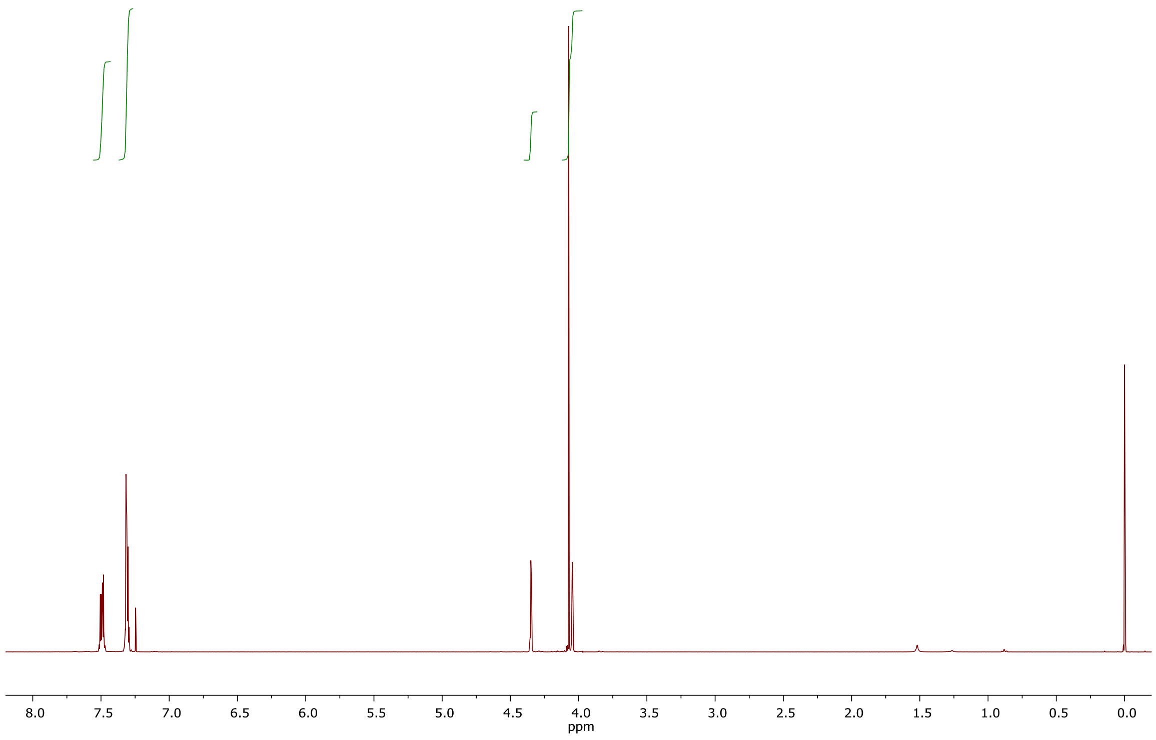

Figure S47. ^{1}H NMR spectrum (100.58 MHz, $CDCl_3$) of **4**.

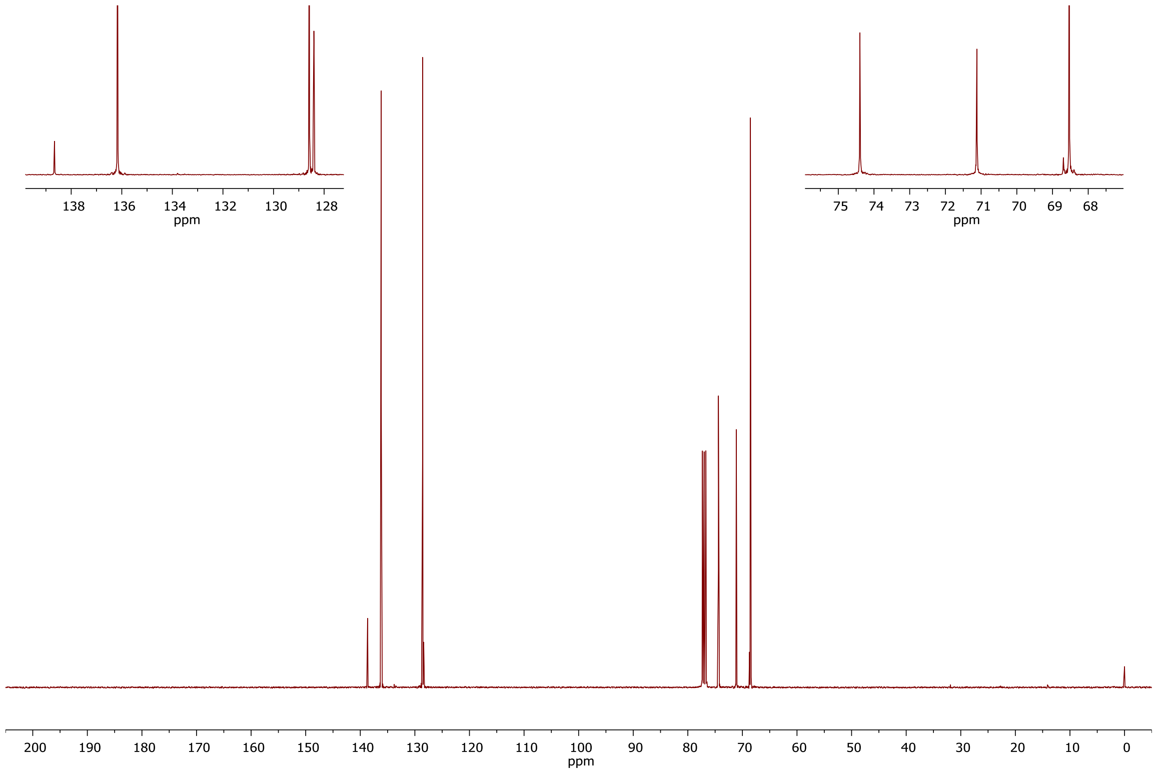

Figure S48. $^{13}C\{^{1}H\}$ NMR spectrum (100.58 MHz, $CDCl_3$) of **4**.

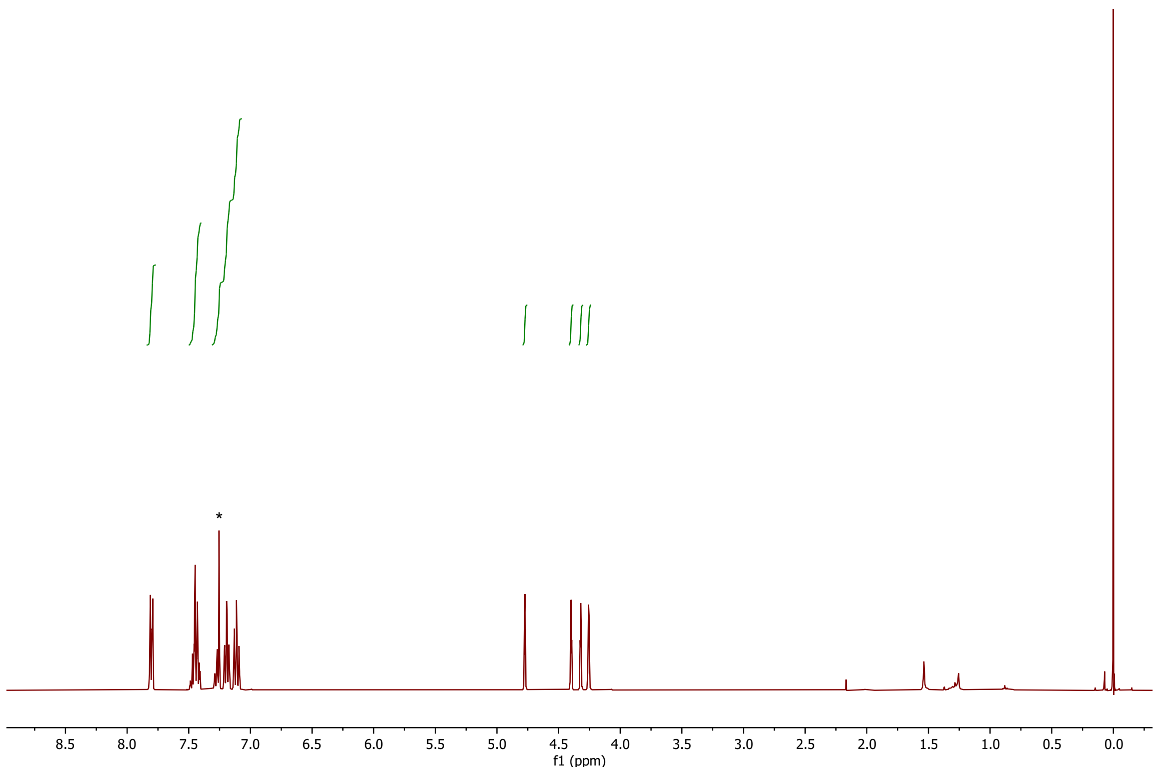

Figure S49. ^{1}H NMR spectrum (100.58 MHz, $CDCl_3$) of **6**.

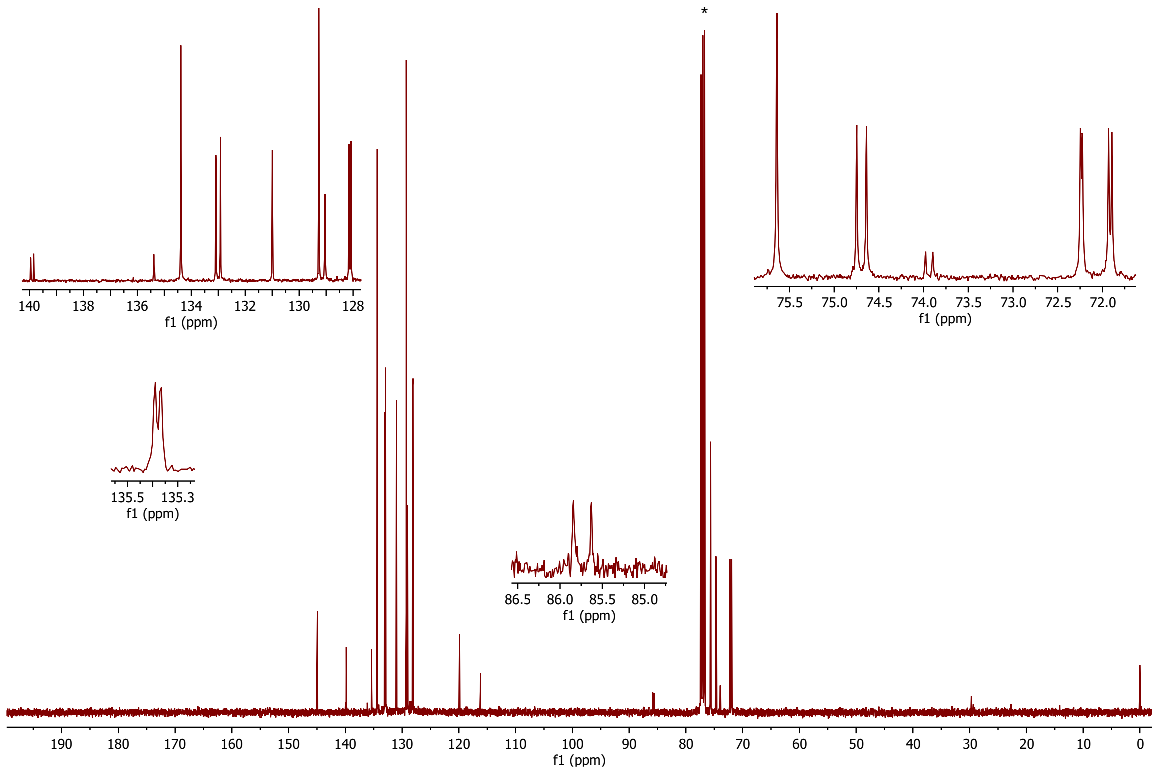

Figure S50. $^{13}C\{^{1}H\}$ NMR spectrum (100.58 MHz, $CDCl_3$) of **6**.

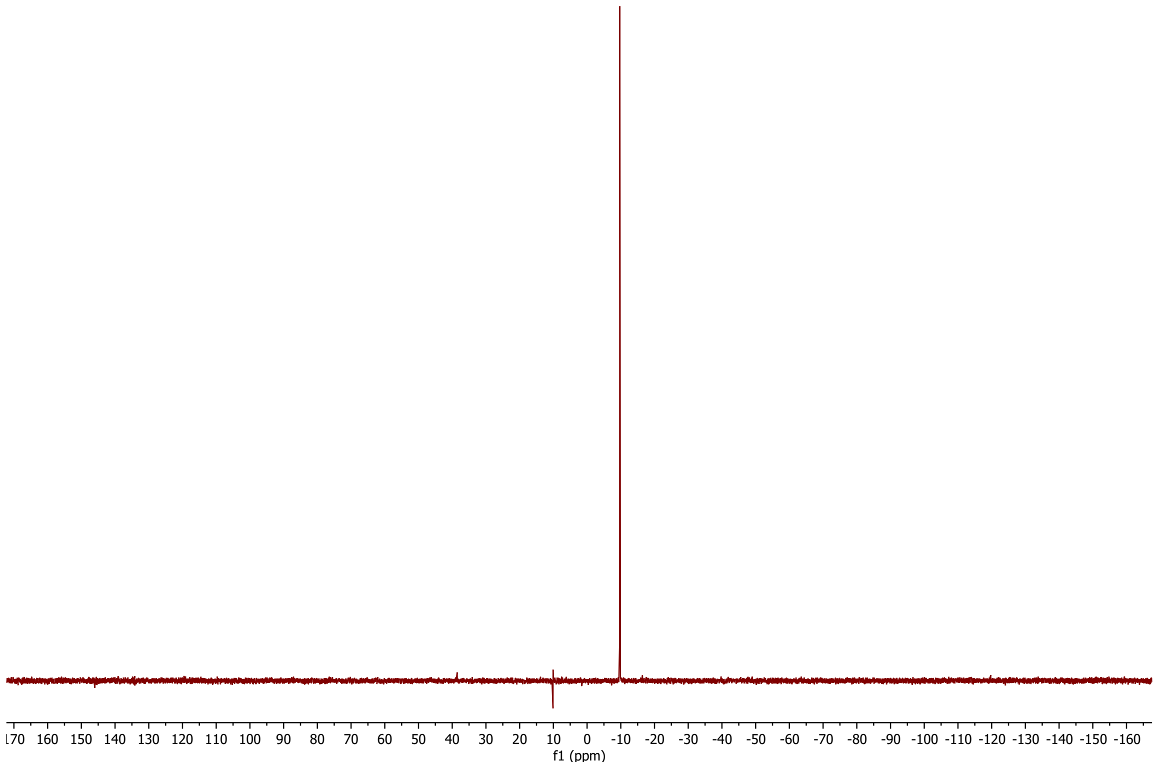

Figure S51. $^{31}P\{^{1}H\}$ NMR spectrum (161.97 MHz, CD_2Cl_2) of **6**.

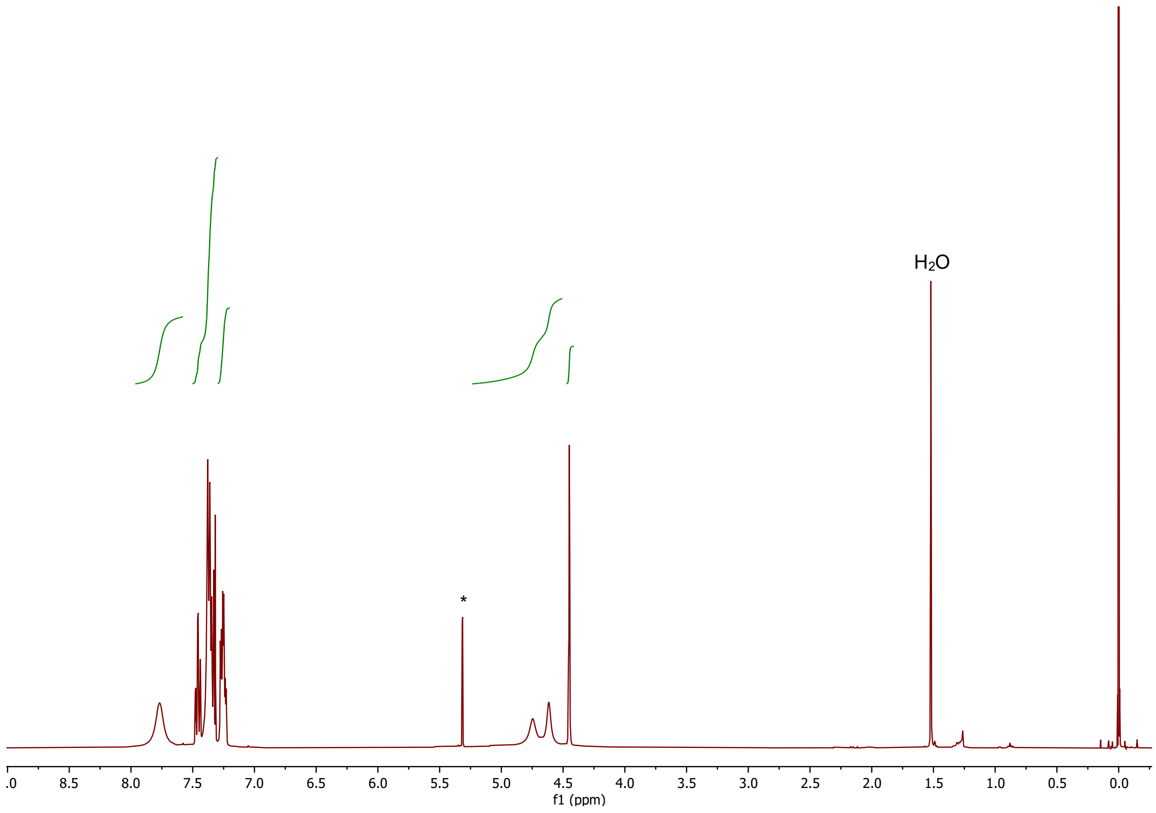

Figure S52. ^{1}H NMR spectrum (100.58 MHz, $CDCl_3$) of **6O**.

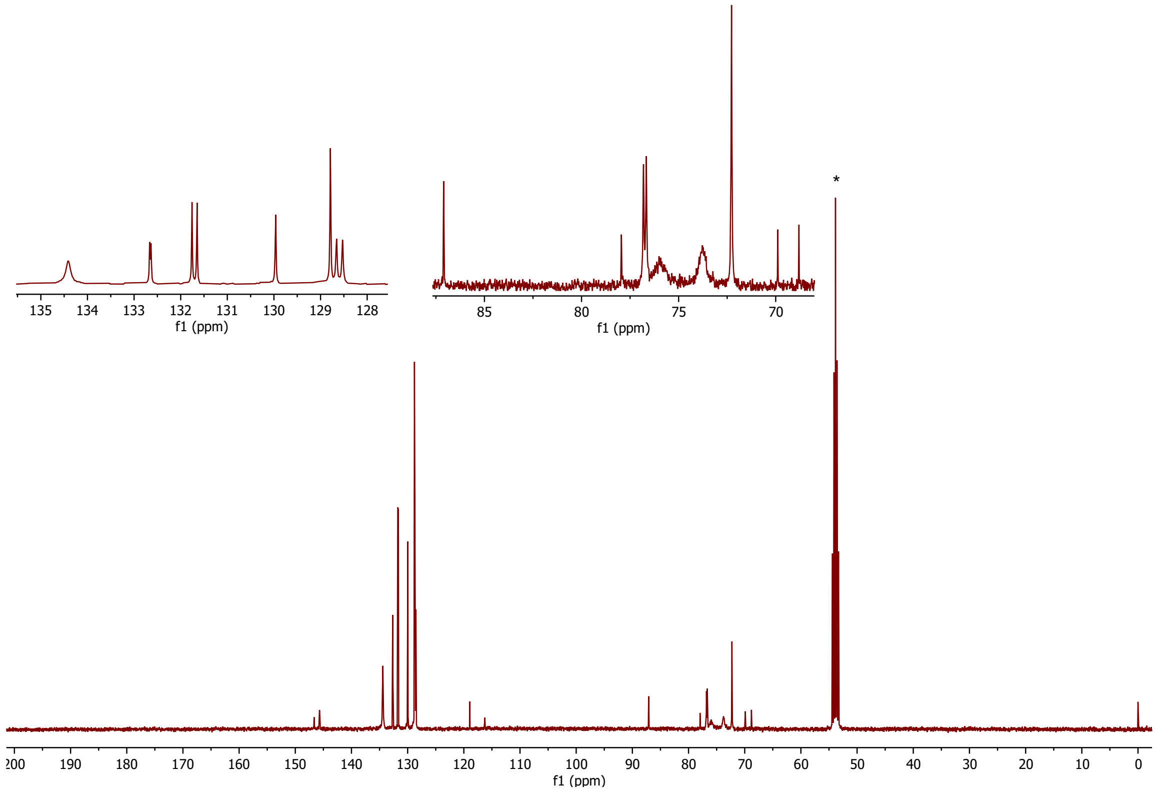

Figure S53. $^{13}C\{^{1}H\}$ NMR spectrum (100.58 MHz, $CDCl_3$) of **6O**.

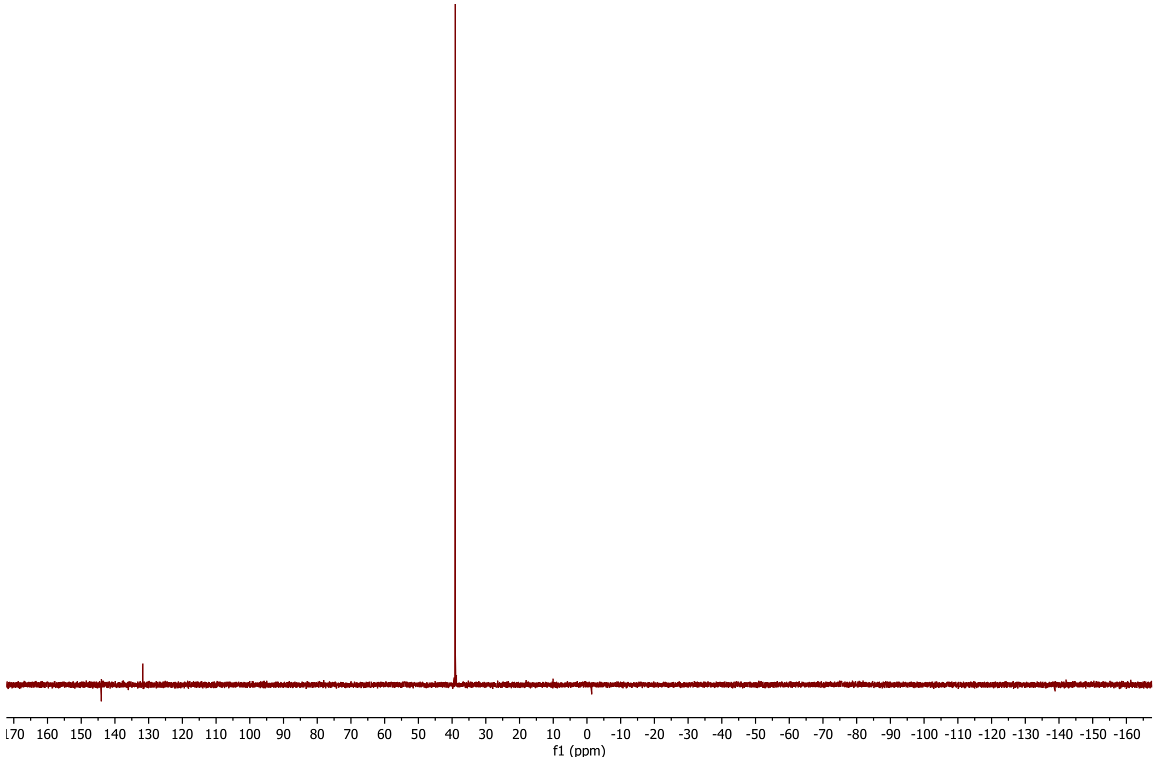

Figure S54. $^{31}P\{^{1}H\}$ NMR spectrum (161.97 MHz, CD_2Cl_2) of **6O**.

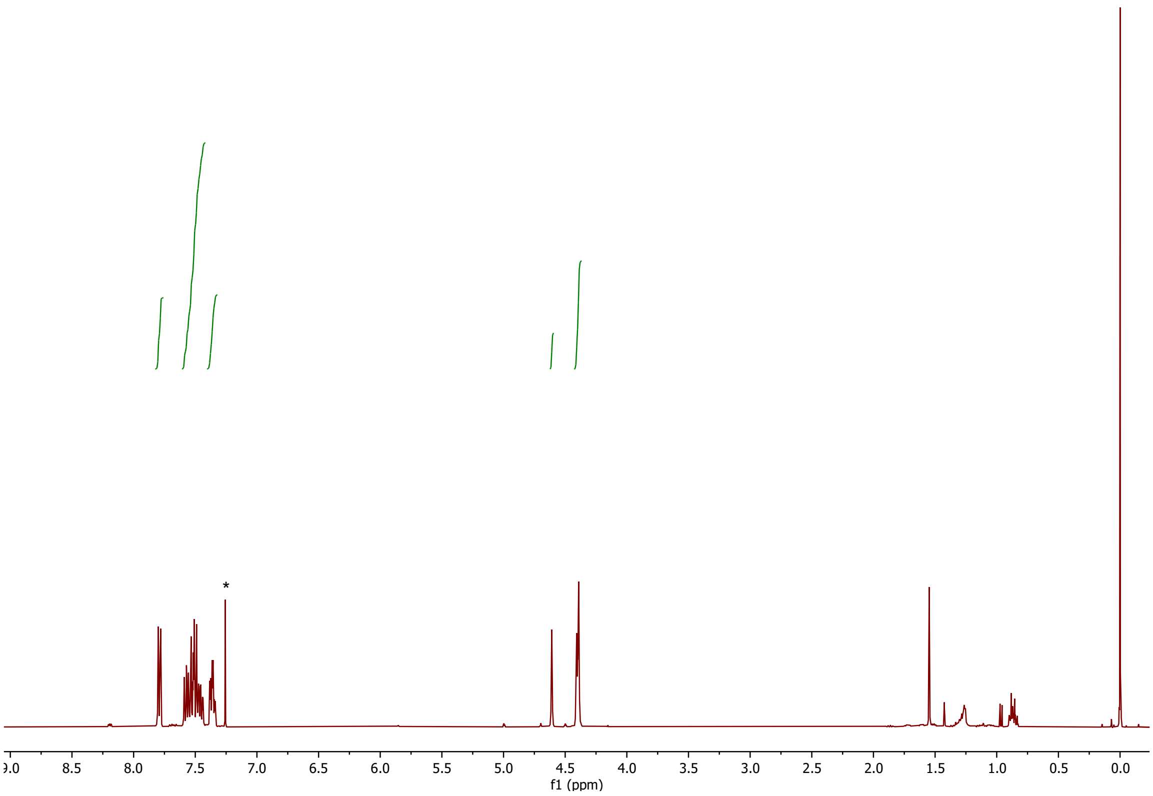

Figure S55. ^{1}H NMR spectrum (100.58 MHz, $CDCl_3$) of **6S**.

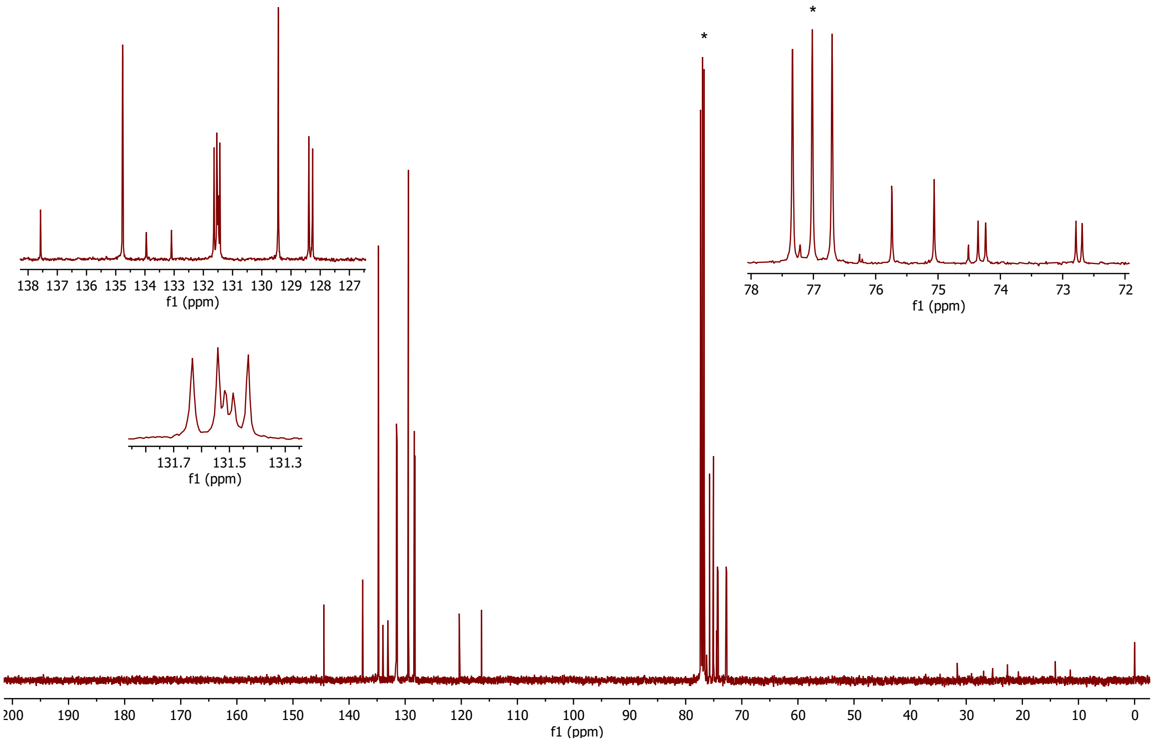

Figure S56. $^{13}C\{^{1}H\}$ NMR spectrum (100.58 MHz, $CDCl_3$) of **6S**.

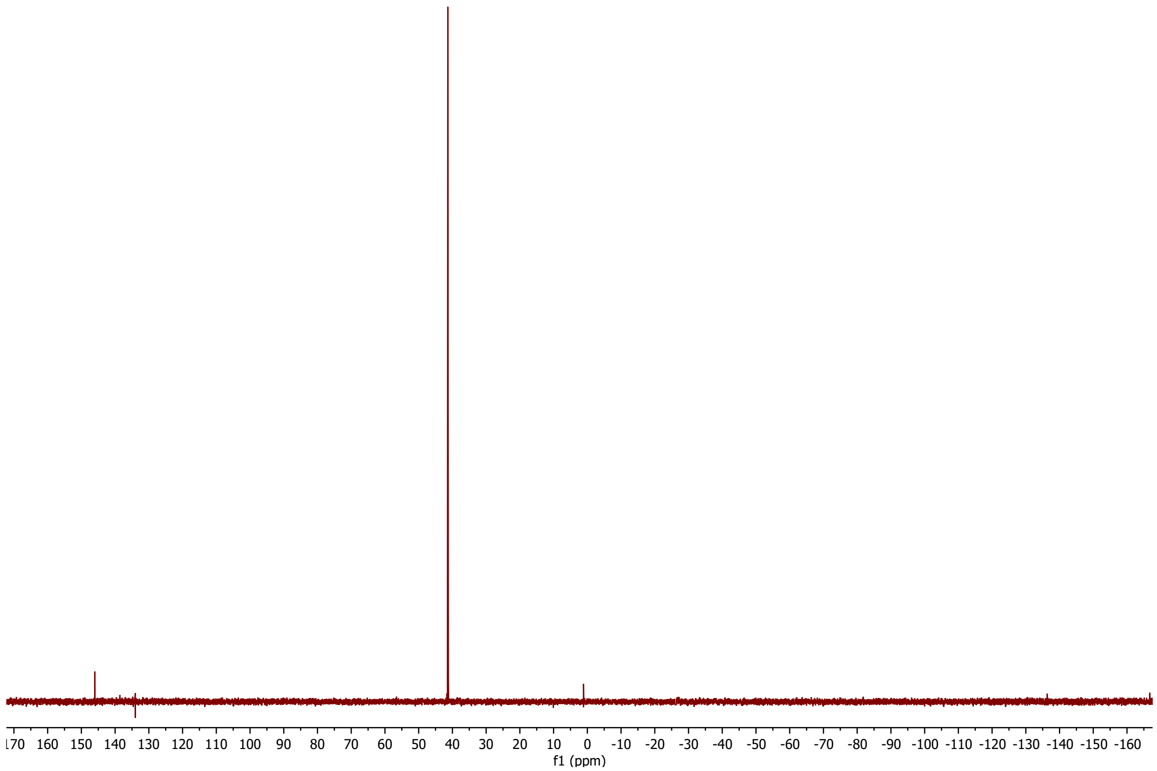

Figure S57. $^{31}P\{^{1}H\}$ NMR spectrum (161.97 MHz, CD_2Cl_2) of **6S**.

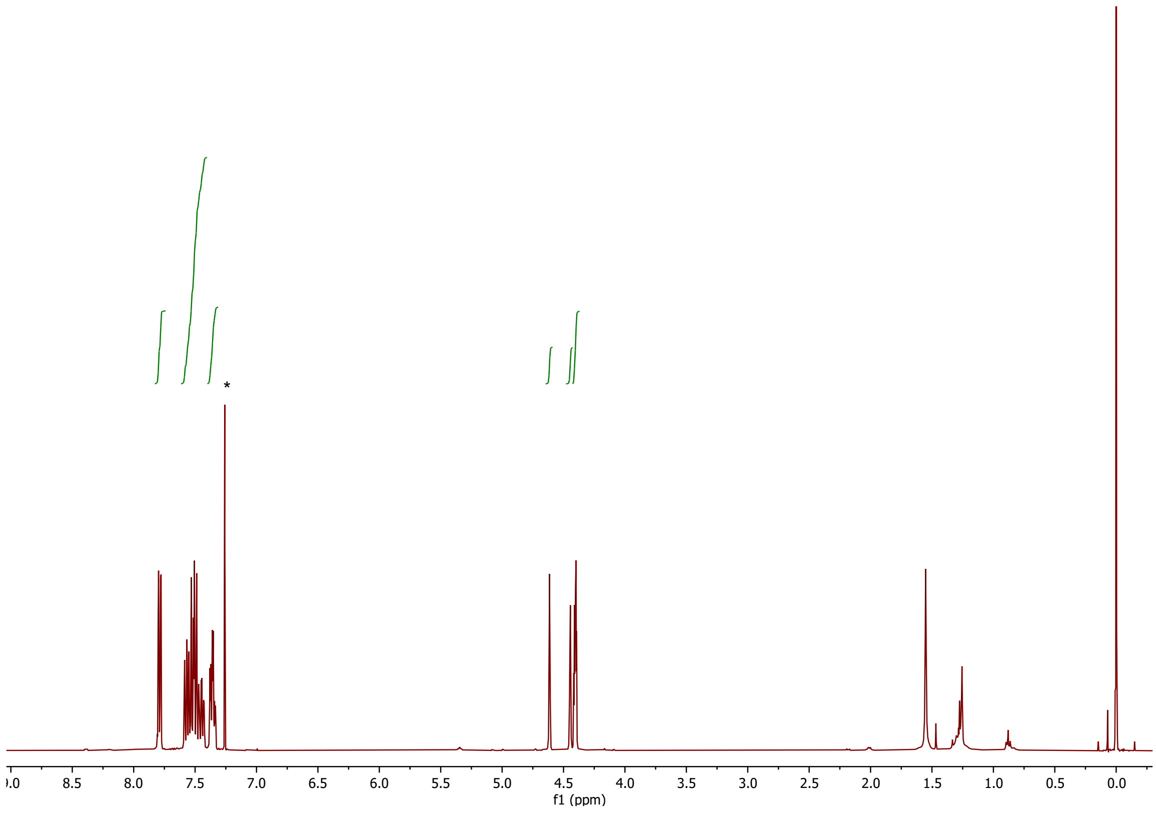

Figure S58. ^{1}H NMR spectrum (100.58 MHz, $CDCl_3$) of **6Se**.

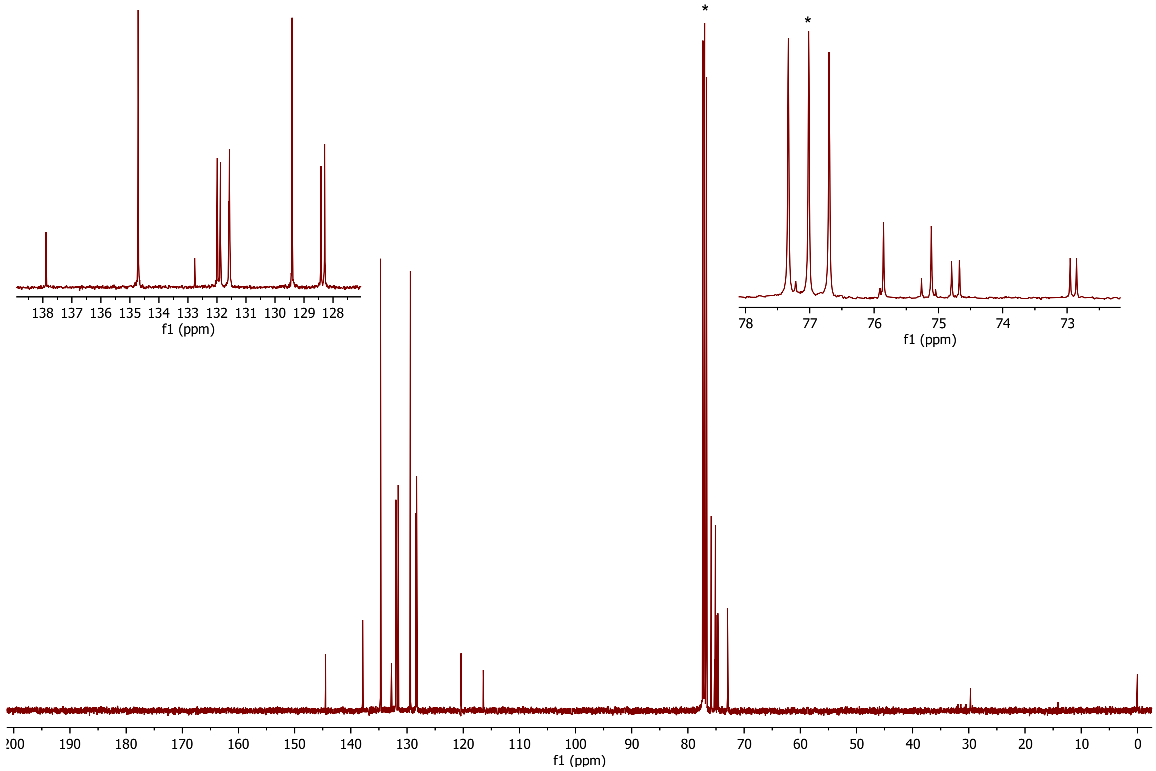

Figure S59. $^{13}C\{^{1}H\}$ NMR spectrum (100.58 MHz, $CDCl_3$) of **6Se**.

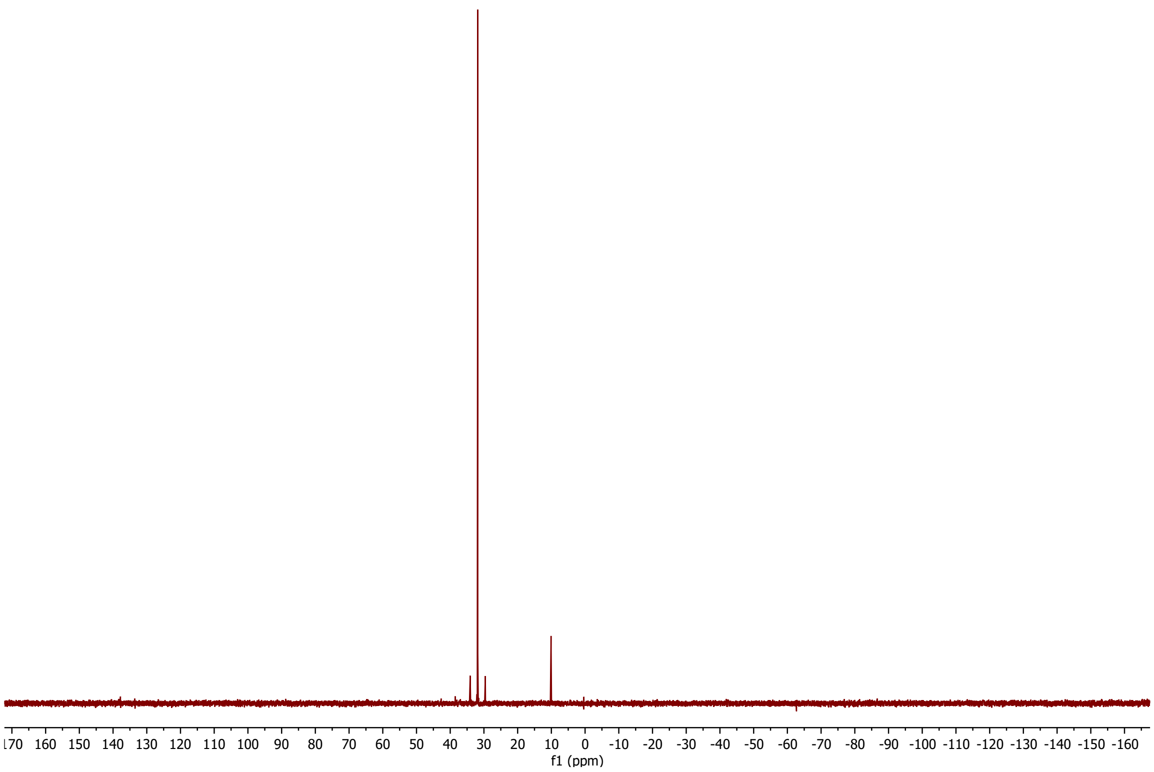

Figure S60. $^{31}P\{^1H\}$ NMR spectrum (161.97 MHz, CD_2Cl_2) of **6Se** (the signal at $\delta_P \approx 10$ is a spike)

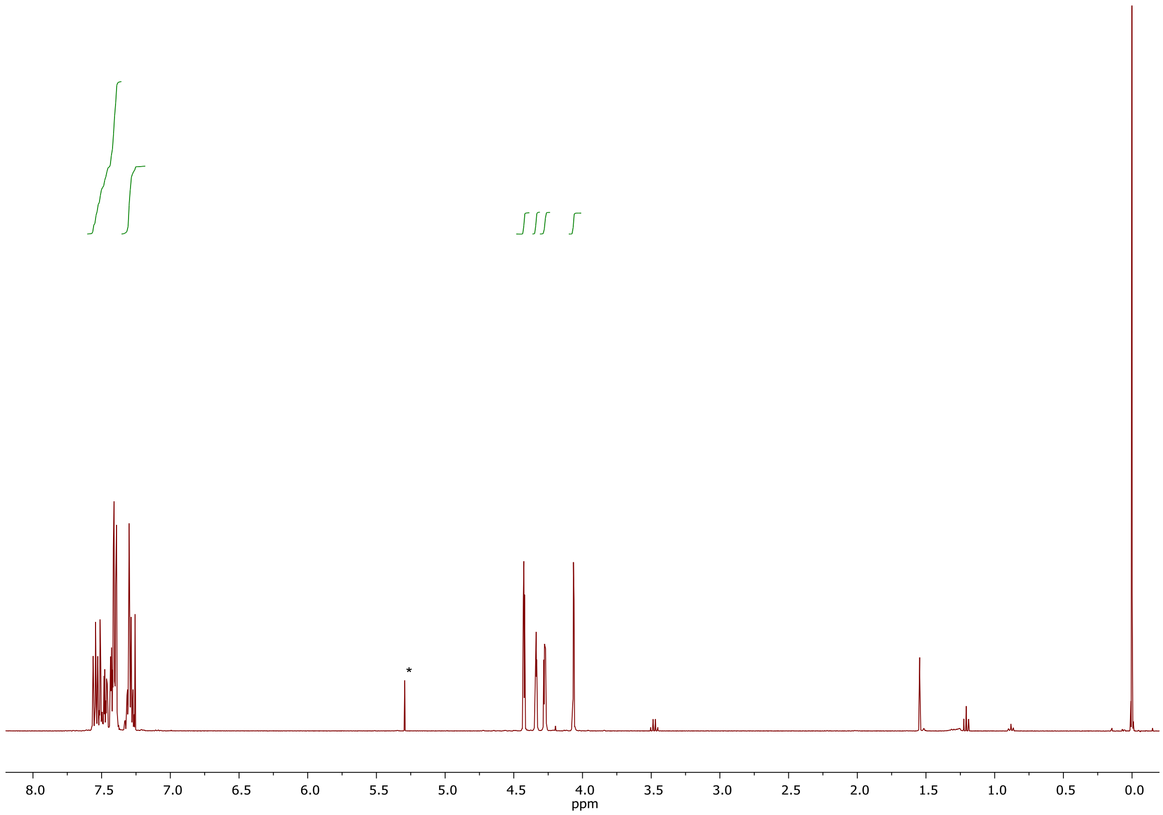

Figure S61. ^{1}H NMR spectrum (100.58 MHz, $CDCl_3$) of **7**.

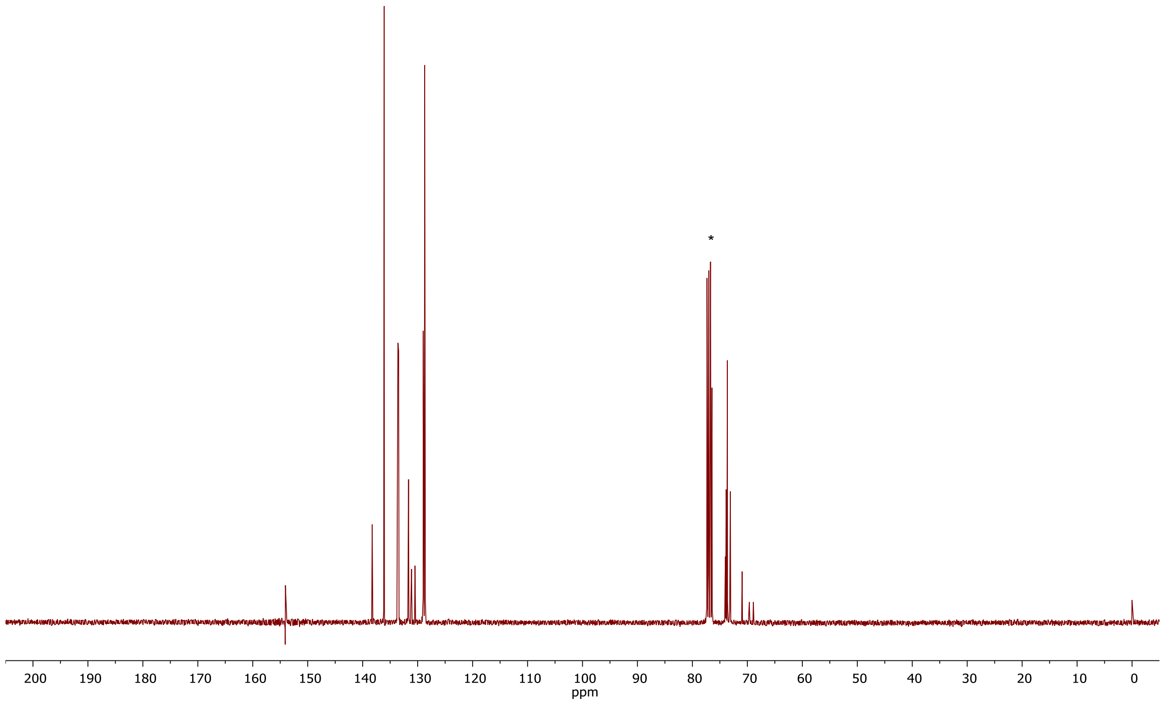

Figure S62. $^{13}C\{^{1}H\}$ NMR spectrum (100.58 MHz, $CDCl_3$) of **7**.

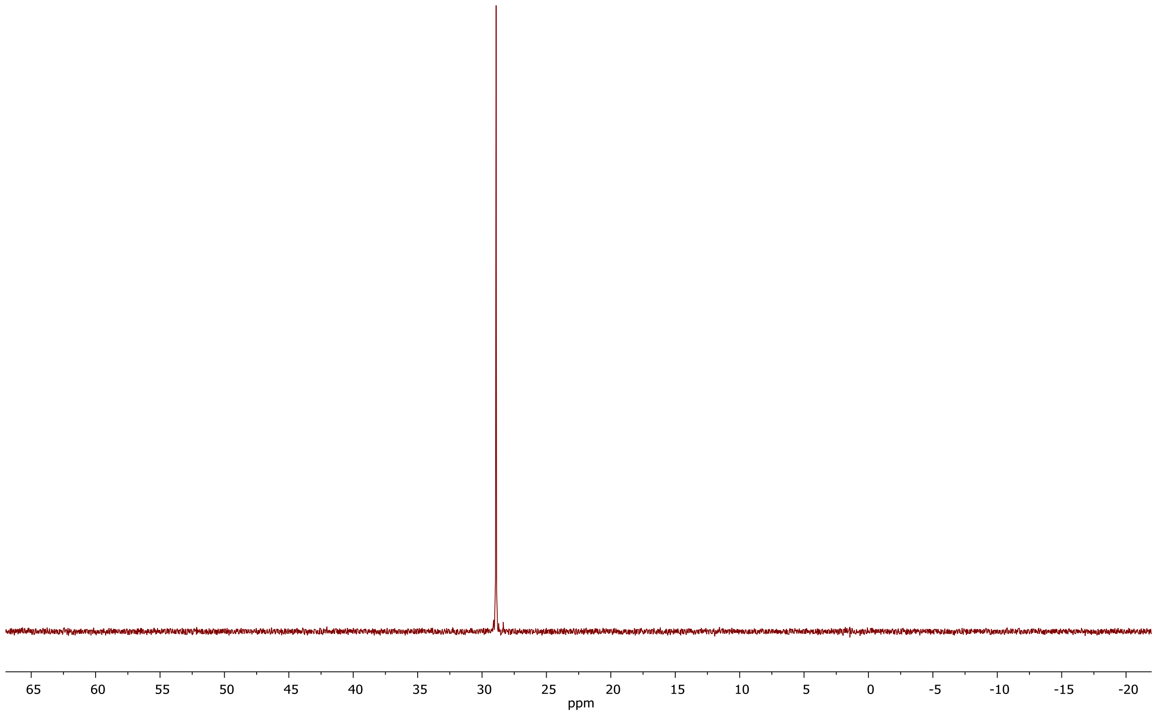

Figure S63. $^{31}P\{^1H\}$ NMR spectrum (161.97 MHz, CD_2Cl_2) of **7**.

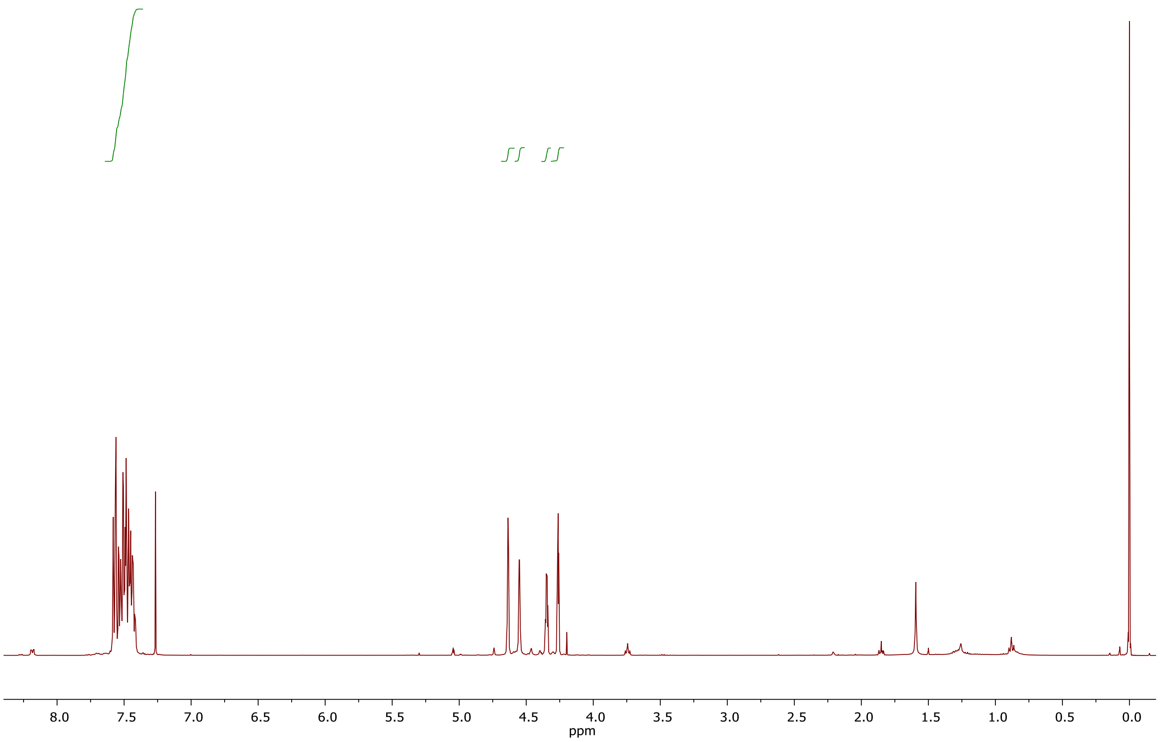

Figure S64. ^{1}H NMR spectrum (100.58 MHz, $CDCl_3$) of **8**.

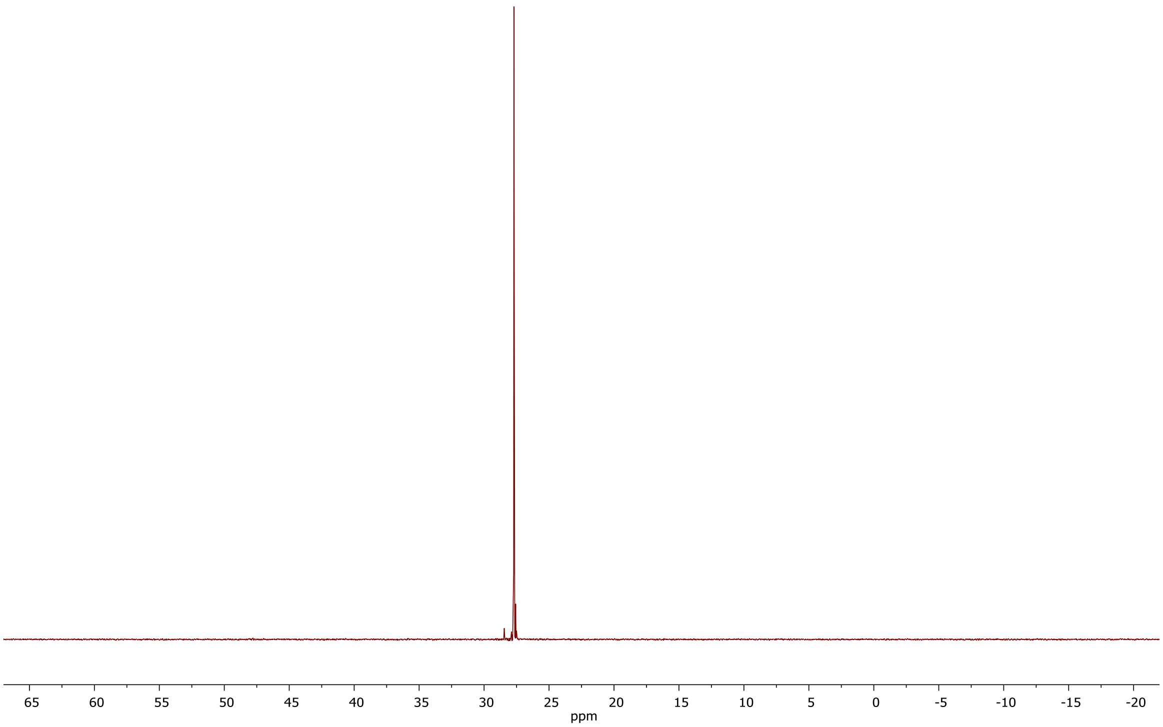

Figure S65. $^{31}P\{^{1}H\}$ NMR spectrum (161.97 MHz, CD_2Cl_2) of **8**.

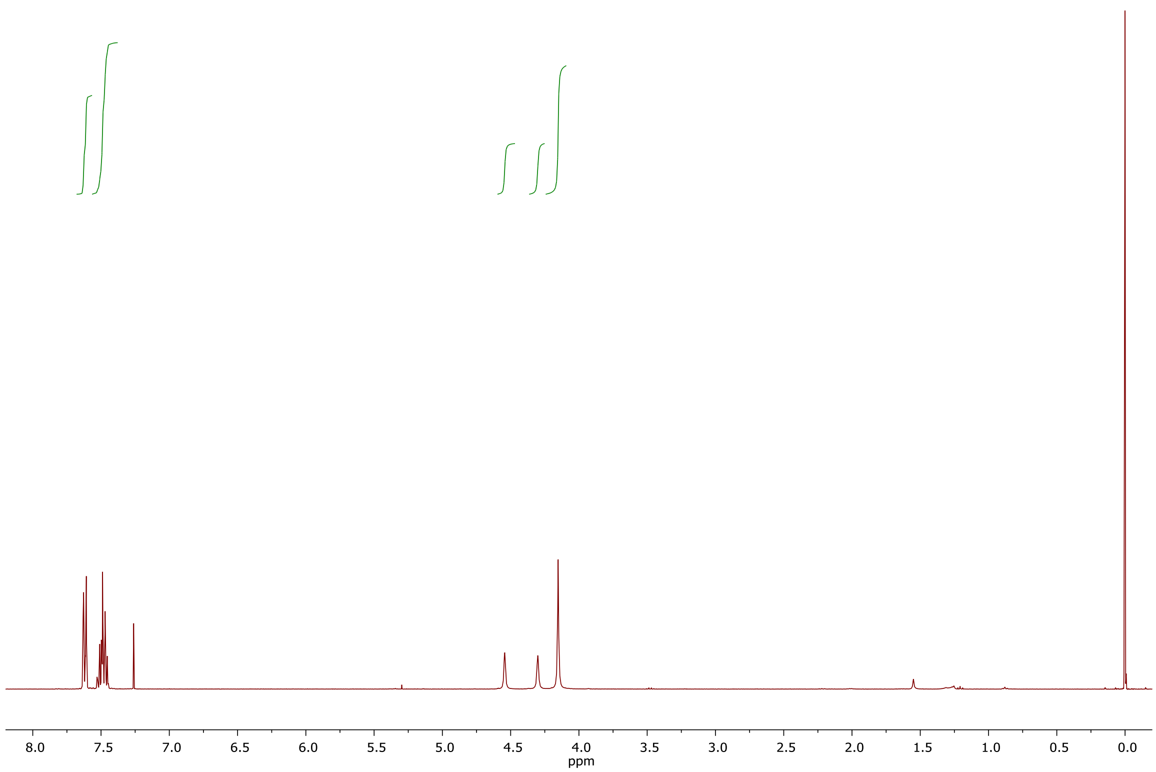

Figure S66. ^{1}H NMR spectrum (100.58 MHz, $CDCl_3$) of **10**.

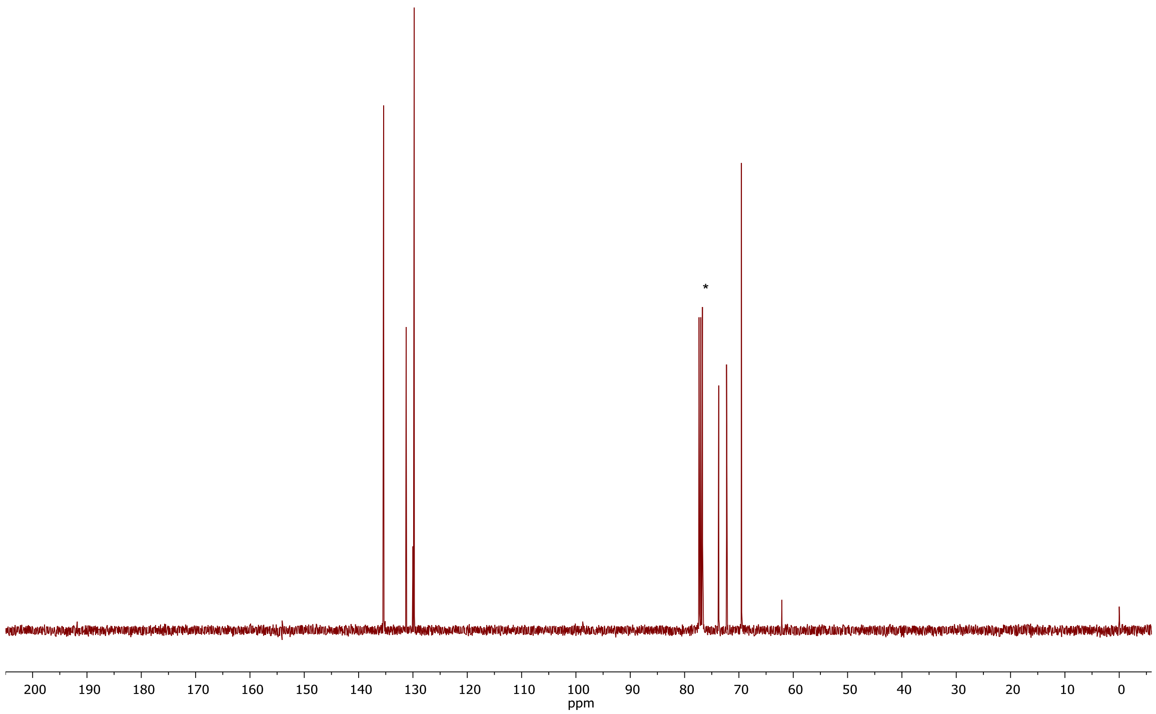

Figure S67. $^{13}C\{^{1}H\}$ NMR spectrum (100.58 MHz, $CDCl_3$) of **10**.

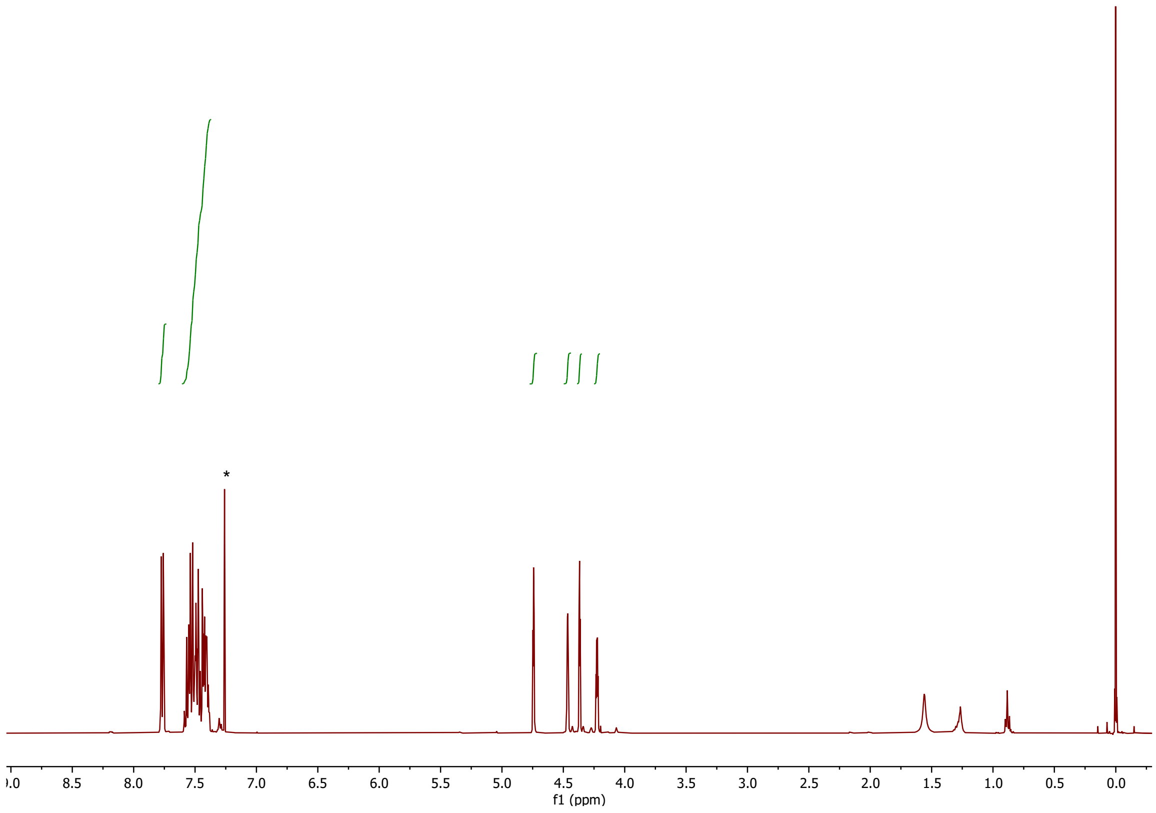

Figure S68. ^{1}H NMR spectrum (100.58 MHz, $CDCl_3$) of **11**.

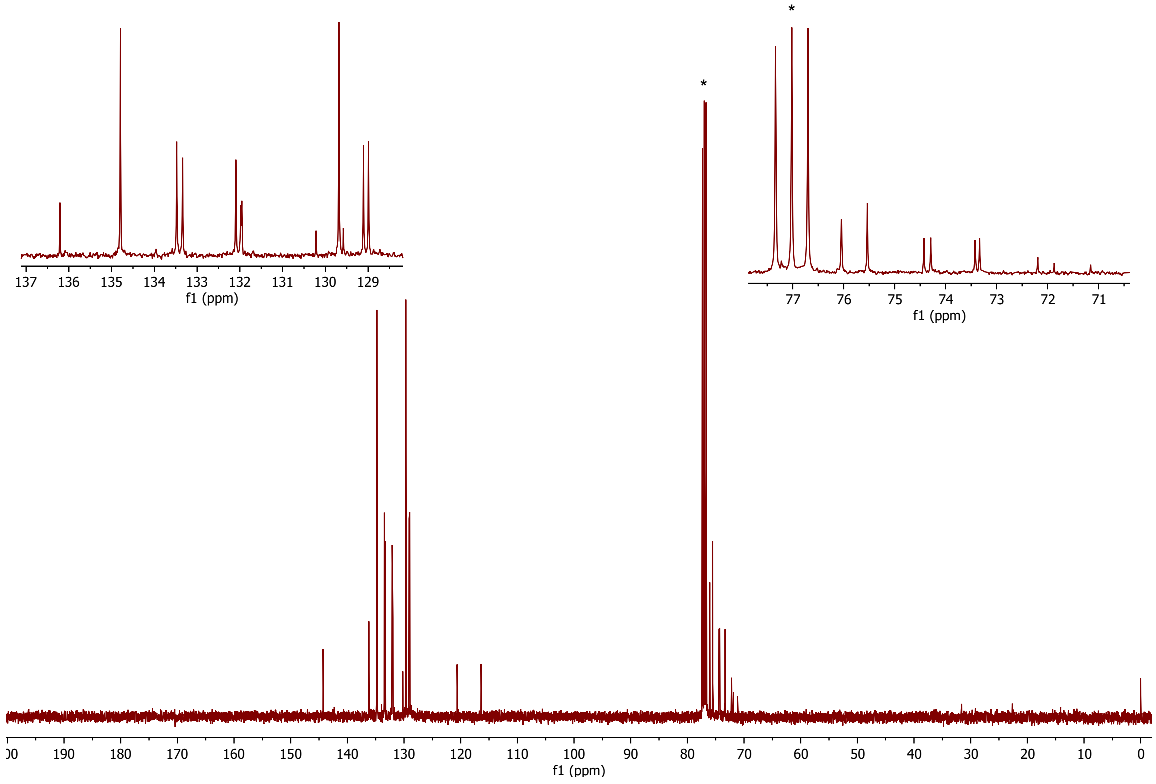

Figure S69. $^{13}C\{^{1}H\}$ NMR spectrum (100.58 MHz, $CDCl_3$) of **11**.

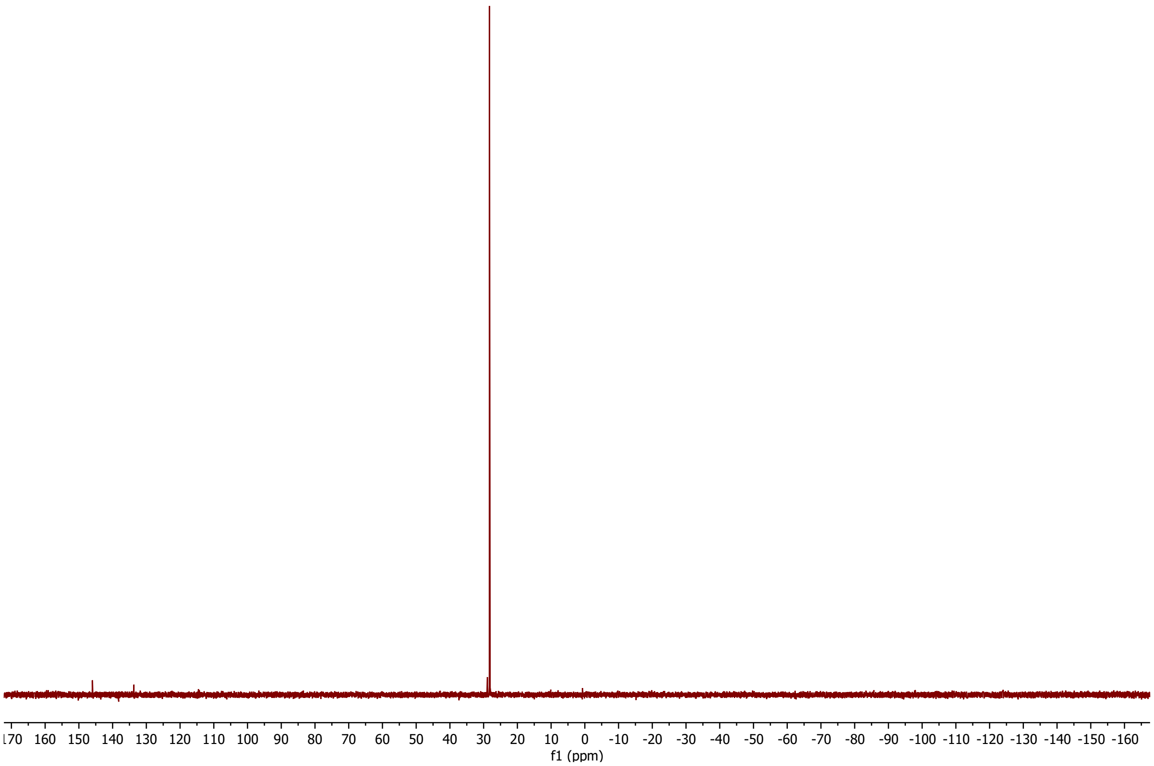

Figure S70. $^{31}P\{^{1}H\}$ NMR spectrum (161.97 MHz, CD_2Cl_2) of **11**.

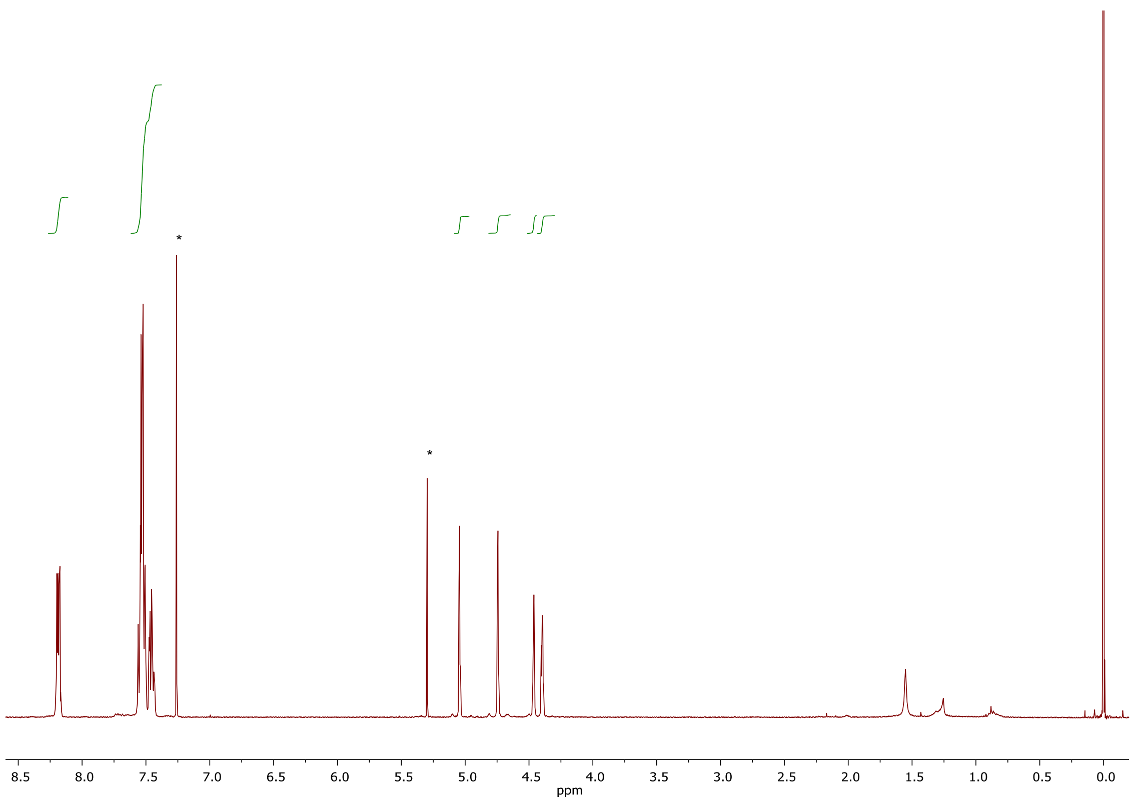

Figure S71. ^{1}H NMR spectrum (100.58 MHz, $CDCl_3$) of **12**.

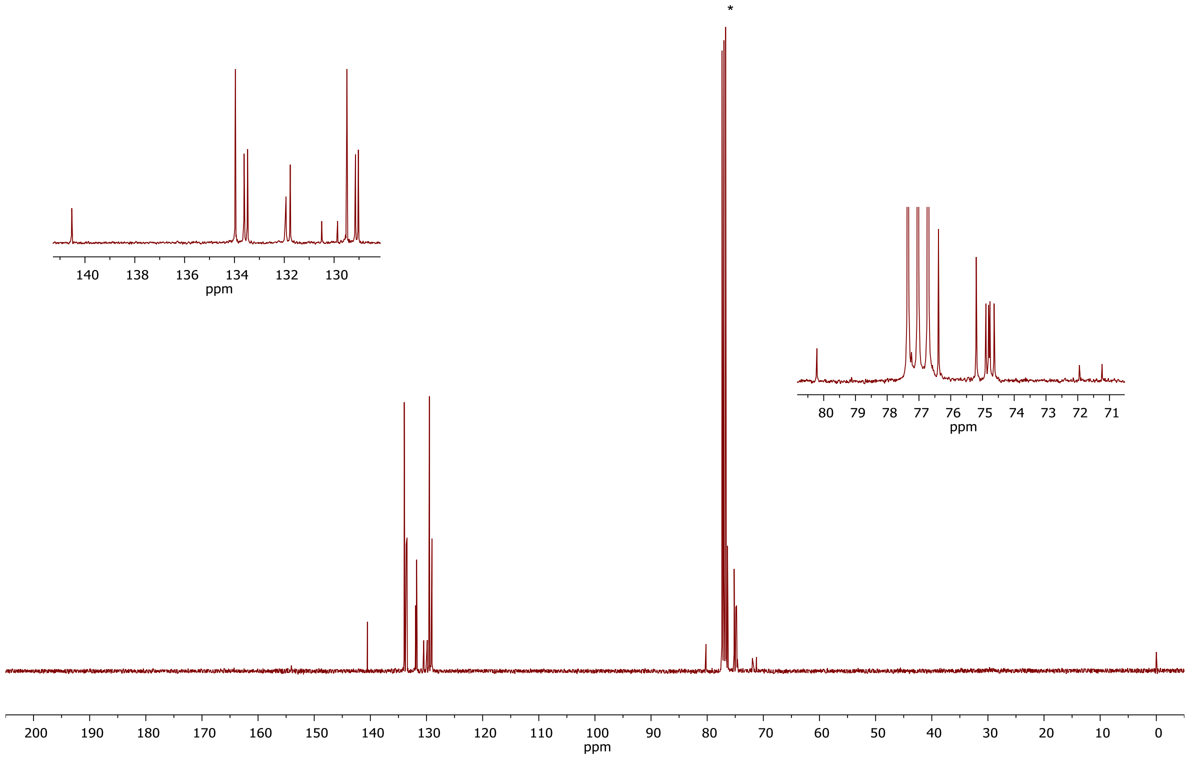

Figure S72. $^{13}C\{^{1}H\}$ NMR spectrum (100.58 MHz, $CDCl_3$) of **12**.

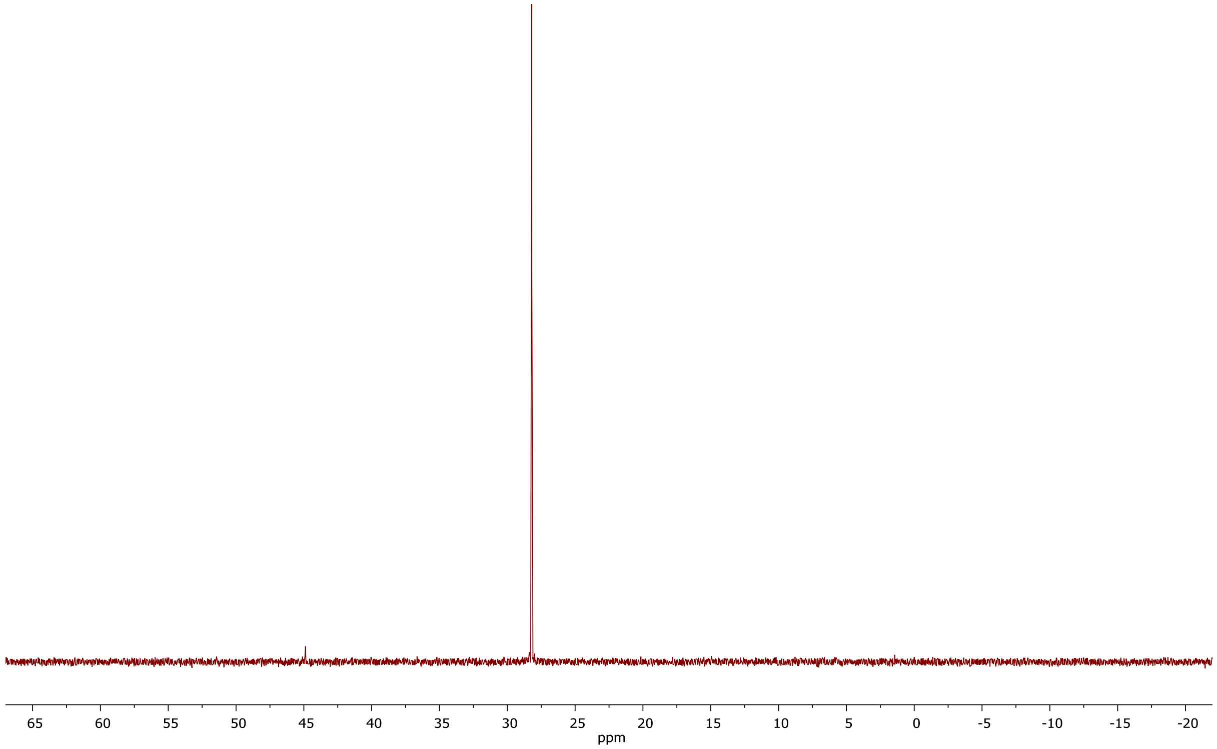

Figure S73. $^{31}P\{^{1}H\}$ NMR spectrum (161.97 MHz, CD_2Cl_2) of **12**.

References

1 Shriver, D. F.; Drezdzon, M. A. The Manipulation of Air-Sensitive Compounds, 2nd ed., J. Wiley & Sons, New York, **1986**.

2 Butler, I. R.; Davies, R. L. A Rapid Convenient Synthesis of Ferrocene-Based Triphos Analogue Ligands. *Synthesis* **1996**, 1350-1354.

3 Škoch, K.; Císařová, I.; Schulz, J.; Siemeling, U.; Štěpnička, P. Synthesis and characterization of 1′-(diphenylphosphino)-1-isocyanoferrocene, an organometallic ligand combining two different soft donor moieties, and its Group 11 metal complexes. *Dalton Trans.* **2017**, *46*, 10339-10354.

4 Chalmers, B. A.; Bühl, M.; Arachchige, K. S. A.; Slawin, A. M. Z.; Kilian, P. Structural, Spectroscopic and Computational Examination of the Dative Interaction in Constrained Phosphine–Stibines and Phosphine–Stiboranes. *Chem. Eur. J.* **2015**, *21*, 7520–7531.

5 a) Smalley, A. W. Ferrocenylphosphines by a revised procedure. *Org. Prep. Proced. Int.* **1978**, *10*, 195-196; b) Kübler, P.; Sundermeyer, J. Ferrocenyl-phosphonium ionic liquids−synthesis, characterisation and electrochemistry. *Dalton Trans.* **2014**, *43*, 3750–6766.

6 Rössler, K.; Rüffer, T.; Walfort, B.; Packheiser, R.; Holze, R.; Zharnikov, M.; Lang, H. Synthesis, characterization and electrochemical behavior of unsymmetric transition metal-terminated biphenyl ethynyl thiols. *J. Organomet. Chem.* **2007**, *692*, 1530-1545.

7 Škoch, K.; Císařová, I.; Štěpnička, P. Synthesis and Catalytic Use of Gold(I) Complexes Containing a Hemilabile Phosphanylferrocene Nitrile Donor. *Chem. Eur. J.* **2015**, *21*, 15998–16004.

8 Bárta, O.; Císařová, I.; Schulz, J.; Štěpnička, P. Assessing the influence of phosphine substituents on the catalytic properties of self-stabilised digold(I) complexes with supporting ferrocene phosphinonitrile ligands. *New J. Chem.* **2019**, *43*, 11258-11262.

9 Silverstein, R. M.; Webster, F. X.; Kiemle, D. J. Spectrometric Identification of Organic Compounds, 7th Ed., Wiley, New York, **2005**; chapter 3, p. 127 ff.

10 Barrière, F.; Geiger, W. E. Use of Weakly Coordinating Anions to Develop an Integrated Approach to the Tuning of $\Delta E_{1/2}$ Values by Medium Effects. *J. Am. Chem. Soc.* **2006**, *128*, 3980-3989.

11 Meng, X.; Kim, S. Tungsten and molybdenum catalyst-mediated cyclisation of *N*-propargyl amides. *Org. Biomol. Chem.* **2011**, *9*, 4429-4431.

12 Sheldrick, G. M. *SHELXT* – Integrated space-group and crystal-structure determination. *Acta Crystallogr., Sect. A: Found. Adv*. **2015**, *71*, 3-8.

13 Sheldrick, G. M. Crystal Structure Refinement with *SHELXL*. *Acta Crystallogr., Sect. C: Struct. Chem.* **2015**, *71*, 3-8.

14 Spek, A. L. PLATON SQUEEZE: a tool for the calculation of the disordered solvent contribution to the calculated structure factors. *Acta Crystallogr., Sect. C: Struct. Chem*. **2015**, *71*, 9-18.

15 a) Spek, A. L. Single-crystal structure validation with the program *PLATON*. *J. Appl. Crystallogr*. **2003**, *36*, 7-13; b) Spek, A. L. Structure validation in chemical crystallography. *Acta Crystallogr. D, Biol. Crystallogr.* **2009**, *65*, 148-155.

16 Frisch, M. J.; Trucks, G. W.; Schlegel, H. B.; Scuseria, G. E.; Robb, M. A.; Cheeseman, J. R.; Scalmani, G.; Barone, V.; Petersson, G. A.; Nakatsuji, H.; Li, X.; Caricato, M.; Marenich, A. V.; Bloino, J.; Janesko, B. G.; Gomperts, R.; Mennucci, B.; Hratchian, H. P.; Ortiz, J. V.; Izmaylov, A. F.; Sonnenberg, J. L.; Williams-Young, D.; Ding, F.; Lipparini, F.; Egidi, F.; Goings, J.; Peng, B.; Petrone, A.; Henderson, T.; Ranasinghe, D.; Zakrzewski, V. G.; Gao, J.; Rega, N.; Zheng, G.; Liang, W.; Hada, M.; Ehara, M.; Toyota, K.; Fukuda, R.; Hasegawa, J.; Ishida, M.; Nakajima, T.; Honda, Y.; Kitao, O.; Nakai, H.; Vreven, T.; Throssell, K.; Montgomery, J. A., Jr.; Peralta, J. E.; Ogliaro, F.; Bearpark, M. J.; Heyd, J. J.; Brothers, E. N.; Kudin, K. N.; Staroverov, V. N.; Keith, T. A.; Kobayashi, R.; Normand, J.; Raghavachari, K.; Rendell, A. P.; Burant, J. C.; Iyengar, S. S.; Tomasi, J.; Cossi, M.; Millam, J. M.; Klene, M.; Adamo, C.; Cammi, R.; Ochterski, J. W.; Martin, R. L.; Morokuma, K.; Farkas, O.; Foresman, J. B.; Fox, D. J. Gaussian 16, Revision C.01; Gaussian Inc.: Wallingford, CT, 2016.

17 a) Perdew, J. P.; Burke, K.; Ernzerhof, M. Generalized Gradient Approximation Made Simple. *Phys. Rev. Lett.* **1996**, *77*, 3865; b) Perdew, J. P.; Ernzerhof, M.; Burke, K. Rationale for Mixing Exact Exchange with Density Functional Approximations. *J. Chem. Phys.* **1996**, *105*, 9982-9985; c) Ernzerhof, M.; Scuseria, G. E. Assessment of the Perdew−Burke−Ernzerhof Exchange-Correlation Functional. *J. Chem. Phys.* **1999**, *110*, 5029-5036; d) Adamo, C.; Barone, V. Toward Reliable Density Functional Methods without Adjustable Parameters: The PBE0 model. *J. Chem. Phys.* **1999**, *110*, 6158-6170.

18 a) Dolg, M.; Wedig, U.; Stoll, H.; Preuss, H. Energy-adjusted Ab Initio Pseudopotentials for the First Row Transition Elements. *J. Chem. Phys.* **1987**, *86*, 866-872; b) Bergner, A.; Dolg, M.; Küchle, W.; Stoll, H.; Preuss, H. Ab Initio Energy-adjusted Pseudopotentials for Elements of Groups 13−17. *Mol. Phys.* **1993**, *80*, 1431-1441; c) Igel-Mann, G.; Stoll, H.; Preuss, H. Pseudopotentials for Main Group Elements (IIIa through VIIa). *Mol. Phys.* **1988**, *65*, 1321-1328.

19 a) Weigend, F.; Furche, F.; Ahlrichs, R. Gaussian Basis Sets of Quadruple Zeta Valence Quality for Atoms H−Kr. *J. Chem. Phys.* **2003**, *119*, 12753-12762; b) Weigend, F.; Ahlrichs, R. Balanced Basis

Sets of Split Valence, Triple Zeta Valence and Quadruple Zeta Valence Quality for H to Rn: Design and Assessment of Accuracy. *Phys. Chem. Chem. Phys.* **2005**, *7*, 3297-3305.

20 a) Becke, A. D.; Johnson, E. R. A density-functional model of the dispersion interaction. *J. Chem. Phys.* **2005**, *123*, 154101; b) Grimme, S.; Antony, J.; Ehrlich, S.; Krieg, H. A Consistent and Accurate Ab Initio Parametrization of Density Functional Dispersion Correction (DFT-D) for the 94 Elements H-Pu. *J. Chem. Phys.* **2010**, *132*, 154104; c) Grimme, S.; Ehrlich, S.; Goerigk, L. Effect of the Damping Function in Dispersion Corrected Density Functional Theory. *J. Comput. Chem.* **2011**, *32*, 1456-1465.

21 a) Tomasi, J.; Mennucci, B.; Cammi, R. Quantum Mechanical Continuum Solvation Models. *Chem. Rev.* **2005**, *105*, 2999-3094; b) Scalmani, G.; Frisch, M. J. Continuous surface charge polarizable continuum models of solvation. I. General formalism. *J. Chem. Phys.* **2010**, *132*, 114110.

22 Lidner, C.; Maryasin, B.; Richter, F.; Zipse, H. Methyl Cation Affinity (MCA) Values for Phosphanes. *J. Phys. Org. Chem.* **2010**, *23*, 1036-1042.

23 Erdmann, P.; Leitner, J.; Schwarz, J.; Greb, L. An Extensive Set of Accurate Fluoride Ion Affinities for p-Block Element Lewis Acids and Basic Design Principles for Strong Fluoride Ion Acceptors. *ChemPhysChem* **2020**, *21*, 987-994.

24 Zhao, Y.; Truhlar, D. G. Design of Density Functionals That Are Broadly Accurate for Thermochemistry, Thermochemical Kinetics, and Nonbonded Interactions. *J. Phys. Chem. A* **2005**, *109*, 5656-5667

25 Lu, T.; Chen, F. Calculation of Molecular Orbital Composition. *Acta Chim. Sin.* **2011**, *69*, 2393-2406.

26 Lu, T.; Chen, F. Multiwfn: A Multifunctional Wavefunction Analyzer. *J. Comput. Chem.* **2012**, *33*, 580-592.

27 a) Avogadro: An Open-Source Molecular Builder and Visualization Tool, version 1.2.0, http://avogadro.openmolecules.net/; b) Hanwell, M. D.; Curtis, D. E.; Lonie, D. C.; Vandermeersch, T.; Zurek, E.; Hutchison, G. R. Avogadro: an Advanced Semantic Chemical Editor, Visualization, and Analysis Platform. *J. Cheminf.* **2012**, *4*, 17.

28 a) G. Knizia, http://www.iboview.org; b) Knizia, G. Intrinsic Atomic Orbitals: An Unbiased Bridge between Quantum Theory and Chemical Concepts. *J. Chem. Theory Comput.* **2013**, *9*, 4834-4843; c) Knizia, G.; Klein, J. E. M. N. Electron Flow in Reaction Mechanisms–Revealed from First Principles. *Angew. Chem., Int. Ed.* **2015**, *54*, 5518-5522.
